# Regioselective Mercury(I)/Palladium(II)-Catalyzed Single-Step Approach for the Synthesis of Imines and 2-Substituted Indoles

**DOI:** 10.3390/molecules26134092

**Published:** 2021-07-05

**Authors:** Rsuini U. Gutiérrez, Mayra Hernández-Montes, Aarón Mendieta-Moctezuma, Francisco Delgado, Joaquín Tamariz

**Affiliations:** 1 Departamento de Química Orgánica, Escuela Nacional de Ciencias Biológicas, Instituto Politécnico Nacional, Prolongación de Carpio y Plan de Ayala s/n, Mexico City 11340, Mexico; rsuini.uri@gmail.com (R.U.G.); encb.mayra2020@gmail.com (M.H.-M.); jfdelgador@gmail.com (F.D.); 2 Centro de Investigación en Biotecnología Aplicada, Instituto Politécnico Nacional, Carretera Estatal Santa Inés Tecuexcomac-Tepetitla, Km 1.5, Tlaxcala 90700, Mexico; amendieta@ipn.mx

**Keywords:** mercury(I)-catalyzed alkyne hydroamination, imines, Pd-catalyzed oxidative cyclization, one-pot indole synthesis

## Abstract

An efficient synthesis of ketimines was achieved through a regioselective Hg(I)-catalyzed hydroamination of terminal acetylenes in the presence of anilines. The Pd(II)-catalyzed cyclization of these imines into the 2-substituted indoles was satisfactorily carried out by a C-H activation. In a single-step approach, a variety of 2-substituted indoles were also generated via a Hg(I)/Pd(II)-catalyzed, one-pot, two-step process, starting from anilines and terminal acetylenes. The arylacetylenes proved to be more effective than the alkyl derivatives.

## 1. Introduction

**Figure 1:**
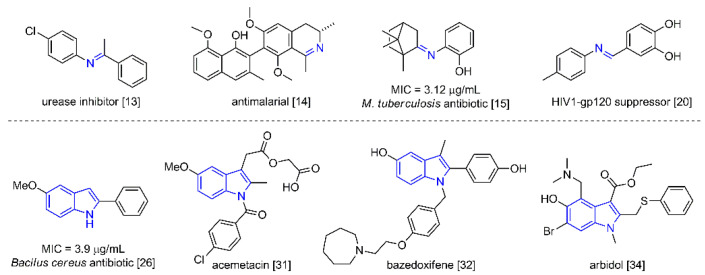
Imine- and indole-containing compounds with pharmacological activity.

Imines are one of the most versatile functional groups in synthesis. Numerous studies have focused on their preparation [1,2,3,4,5], chemical structure and tautomeric or transimination equilibrium [6,7,8], and use as a precursor for the construction of more complex structures [9,10,11,12]. Due to their versatility, they play an important role in medicinal chemistry, serving as urease and protease inhibitors [13] as well as antimalarial, antibacterial, and antifungal agents [14]. They also exhibit activity against *Mycobacterium tuberculosis* [15] and tumor cells [16,17,18,19], and act as suppressors of HIV1-gp120, related to the human immunodeficiency virus (HIV) [20] (Figure 1). Interestingly, imines are present in phytoplankton and shellfish as toxins [21].

Likewise, indoles are widely distributed in natural products [22,23,24]. They occupy a prominent place as pharmacologically active compounds [25,26,27,28], including commercial therapeutic chemicals and active pharmaceutical ingredients [29,30,31]. The U.S. Food and Drug Administration (FDA) has approved at least 43 indole-containing drugs [32,33]. For example, arbidol has shown efficient in vitro activity against the SARS-CoV-2 infections [34] and has been administered to infected patients [35,36,37] (Figure 1). Hence, the molecular structures of imines and indoles are of great value for medicinal and synthetic purposes.

Despite the variety of methods for the preparation of imines [1,2,3,4,5], their synthesis is mainly carried out with conventional procedures, such as the acid-catalyzed condensation of a carbonyl compound with a primary amine [1]. Among the relevant methodologies are the reduction of nitriles, oximes, and aliphatic nitro derivatives [38,39,40,41], the addition of an organometallic reagent to nitriles and amides [42,43], and the oxidation of alcohols and amines [44,45]. These methods are only practical, however, if there is an existing carbon–nitrogen formal bond in the substrate.

**Scheme 1:**
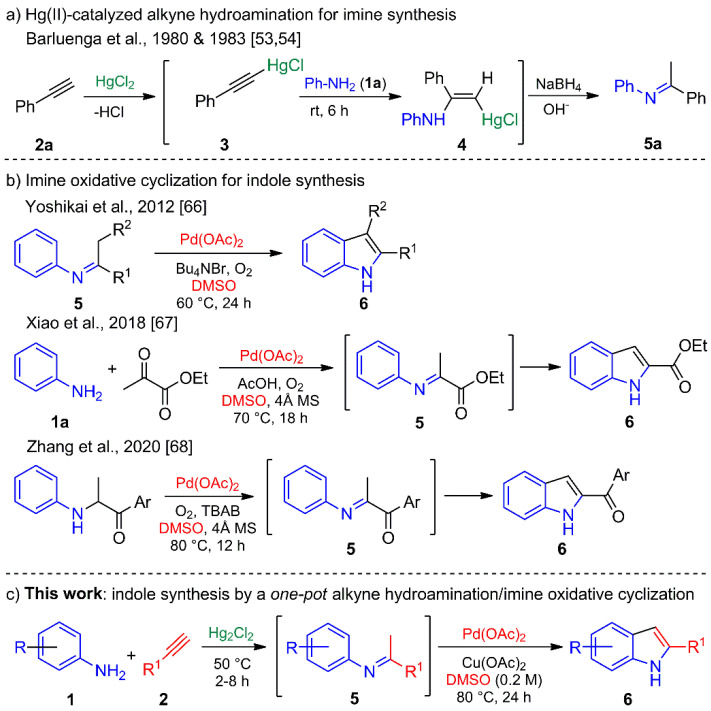
(**a**) Imine synthesis by a Hg(II)-promoted alkyne hydroamination. (**b**) Indole synthesis from imines derived by a condensation reaction and Pd(II)-catalyzed oxidative cyclization. (**c**) Pd(II)-catalyzed indole synthesis from imines derived from Hg(I)-catalyzed alkyne hydroamination.

Intermolecular imine is usually synthesized by condensation reactions with an azeotropic agent [1], a desiccant [46], or a water scavenger [47] to remove water. Alkyne hydroamination arose as an alternative, being an attractive and elegant pathway with complete atom economy [48,49,50,51]. Barluenga et al. described several catalytic and noncatalytic hydroamination methods with terminal acetylenes (e.g., **2a**) promoted by Tl(III) or Hg(II) salts [52]. They found that HgCl_2_ acts through a nucleophilic attack of the aniline (**1a**) on the internal acetylene carbon, firstly forming terminal acetylene-HgCl complex **3** followed by its possible conversion into the aminoalkenylmercurial(II) chloride intermediate **4**. The latter regenerates the Hg(II) catalyst by protonolysis to give the enamine, which is converted into the desired imine **5a** (Scheme 1a) [53,54].

In contrast, indoles have been elaborated by diverse protocols [55], such as the well-known Fischer [56,57], Julia [58], Bartoli [59], and Gassman [60] procedures, each of which include a sigmatropic rearrangement at a high temperature. Other efficient approaches have involved organometallic reagents, as with the syntheses of indoles described by Castro [61], Larock [62], Fagnou [63], and Söderberg [64,65]. Most of them demand the use of *ortho*-halogenated and *ortho*-vinylated N-protected anilines or nitrobenzenes to build the indole A ring. Recently, imine substrates have proven valuable for indole synthesis, as demonstrated by the groups of Yoshikai [66], Xiao [67], and Zhang [68] (Scheme 1b).

Our group has an ongoing interest in designing and carrying out the novel synthesis of heterocycles [69,70,71], and in particular of aza-heterocycles and indoles [72,73,74,75]. Accordingly, the aim of the present study was to assess the feasibility of preparing indoles in a one-pot process, involving the regioselective Hg(I)-catalyzed hydroamination of terminal alkynes to produce imines as intermediates, and their subsequent Pd(II)-catalyzed cross-coupling oxidative cyclization to afford the corresponding C-2-substituted indoles (Scheme 1c).

## 2. Results and Discussion

### 2.1. Synthesis of Imines **5a**–**n** by Hg(I)-catalyzed Hydroamination of Alkynes **2a**–**d** with Anilines **1a**–**k**

The properties of Hg(0) and Hg(II) salts have been examined [76], and these species have many chemical and industrial applications [77,78,79], including chemical transformations [80,81,82]. Curiously, the use of Hg(I) in organic synthesis is surprisingly rare and limited [83,84]. Our group has reported the inclusion of Hg_2_Cl_2_ as the catalyst for the solvent-free hydroamination of phenylacetylene (**2a**) with *m*-anisidine (**1e**) to furnish the corresponding imine **5e** in quantitative yield [85]. Following up on that effort, the scope of the method was herein explored by varying the structure of anilines (**1**) and acetylenes (**2**) to develop an efficient synthesis of imines (**5**). Unfortunately, when employing the previously described reaction conditions (room temperature, 24 h), the yields of the resulting imines depended on the anilines (Table 1). For example, with *p-*anisidine (**1i**) and **2a**, imine **5i** was furnished in a modest yield (61%, entry 2). The yield was improved by increasing the reaction time (82%, entry 3) or by raising the temperature to 45 ºC with a reaction time of 4 h (87%, entry 4).

Other parameters were evaluated, such as solvent, catalyst, and additive. Although solvent-free reaction conditions are always recommended for designing an optimal green chemistry methodology [86], three chlorinated solvents (chloroform, 1,2-dichloroethane, and methylene chloride), THF, and DMSO were assessed by heating at 60 ºC for 2–12 h. Whereas imine **5i** was obtained in fairly low yields (21–41%) with either the first or the third solvent, it was not detected with 1,2-dichloroethane, THF, and DMSO. When Ag_2_CO_3_ (Table 1, entry 5) and CuCl (not shown) were tested as the catalysts for **1e** and **2a**, the outcome was the recovery of the reactants but no trace of imine **5e**.

Imines, usually water-sensitive, are converted into ketones by hydrolysis. For example, acetophenone (**7a**) can be formed from imines **5e** or **5i**. Therefore, anhydrous lithium carbonate was employed as the additive, which greatly improved the yield (Table 1, entries 6 and 7). Of course, under these optimal reaction conditions, the presence of Hg_2_Cl_2_ was essential for the transformation to take place. Due to a feasible light-induced Hg–Hg bond disproportionation [87], the generation of radicals was contemplated. Consequently, hydroquinone was added as a radical quencher, which did not modify the yield (entry 8) and thus ruled out this possibility.

In agreement with the findings of Barluenga et al. [52,53,54], the process was also catalyzed with HgCl_2_ under the same reaction conditions, but a lower yield (78%) was observed (Table 1, entry 9). This suggests that Hg(I) and Hg(II) are both probably the catalytic species involved in the process. Considering the feasible generation of Hg(0) as a secondary product during the decomposition of Hg_2_Cl_2_ under an analogous reaction [53,54], the process was carried out in the presence of Hg(0), but no imine was found (entry 10). Hence, the participation of Hg(0) as a catalyst is unlikely. Moreover, there was no visual evidence of a dark-silver mirror residue in the Hg(I) trials. Actually, Hg(0) is a well-known poisoning catalyst in heterogeneous/homogeneous reactions [88].

To gain insights into the mechanism, **2a** was reacted with Hg_2_Cl_2_ to examine its possible conversion into acetophenone (**7a**) as an intermediate. After heating at 45 ºC for 1 h, however, only the recovery of **2a** was achieved. Taking into account the potential formation of intermediate **7a** in the middle of the reaction, a further trial was performed under the same reaction conditions (45 ºC for 1 h) and in the presence of **1i** and Hg_2_Cl_2_. The result was again the recovery of the staring material. Finally, after reacting a mixture of **1i** and Hg_2_Cl_2_ at 45 ºC for 1 h, no N-Hg complex was detected.

Once the reaction conditions were optimized (Table 1, entry 7), evaluation was performed for a series of anilines (**1a**–**k**) bearing substituents with diverse electron-demand at the three positions of the benzene ring, utilizing arylalkynes **2a**–**d** as the terminal acetylenes (Table 2). In general, neither the electron-demand of the substituents nor their position in the benzene ring of the aniline showed any effect on the efficacy of the process. The only exception was the use of the halogenated anilines **1f** and **1k**, which gave rise to the corresponding imines in modest yields (Table 2, entries 6 and 11).

The high yields afforded by the unsubstituted phenylacetylene (**2a**) were also obtained with the terminal *para-*substituted phenylacetylenes **2b–d** (Table 2, entries 9 and 12–14), supporting the idea that Hg(I) easily interacts with the triple bond regardless of the electron-demand of its substituent. Furthermore, the reaction was successful with terminal alkyl- and some aryl-acetylenes, including 4-cyanophenylacetylene (**2c**), propylacetylene (**2e**), cyclopropylacetylene (**2f**), and cyclohexylacetylene (**2g**), judging by the ^1^H NMR and MS/GC analyses of the crude mixtures. Nevertheless, the corresponding imines **5m** (as a mixture of **5m**/*p*-cyanoacetophenone (**7b**) (95:5)) and **5o**–**q** were not stable enough to be isolated under the extraction conditions. Evidence of their formation was found unambiguously by the synthesis of the indoles (see Scheme 3).

Reactivity significantly decreased with the di-substitution of the triple bond. For instance, neither methylpropylacetylene (**2h**) nor phenylpropylacetylene (**2i**) reacted with *p*-anisidine (**1i**), even when the mixture was heated to 80 ºC for 53 h, probably because both electronic and steric effects impede the addition of the aniline or do not allow for the formation of the σ-Hg(I)-acetylene complex (see below).

**Figure 2:**
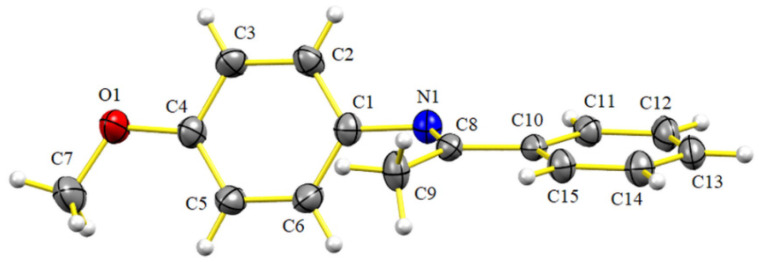
Structure of **5i**, as determined by single X-ray diffraction crystallography (ellipsoids at the 30% probability level).

All the imines prepared by this method were characterized by IR and NMR (1D and 2D), and in some cases, HRMS. The latter technique was performed in case of finding a discrepancy between the data reported in the literature and the melting point or physical state of the compound in the current effort. Imine **5i** was crystallized in order to be subjected to X-ray crystallographic analysis (Figure 2), which demonstrated the expected *E* configuration of the double bond. However, the *N-*anisyl ring was not coplanar to the imine double bond, instead adopting a quasi-orthogonal conformation (torsion angle, C(8)-N(1)-C(1)-C(2) = 115.31(17), see Supplementary Materials), which indicates that the π-system is not conjugated. In contrast, the phenyl ring attached to the imine double bond displayed a planar conformation (torsion angle, C(11)-C(10)-C(8)-N(1) = –4.7(2), see Supplementary Materials).

### 2.2. One-Pot Synthesis of Indoles **6a**–**p** by Subsequent Hg(I)- and Pd(II)-Catalyzed Conversion of Anilines **1a**–**g** and **1i**–**k** with Alkynes **2a**–**g**

**Scheme 2:**
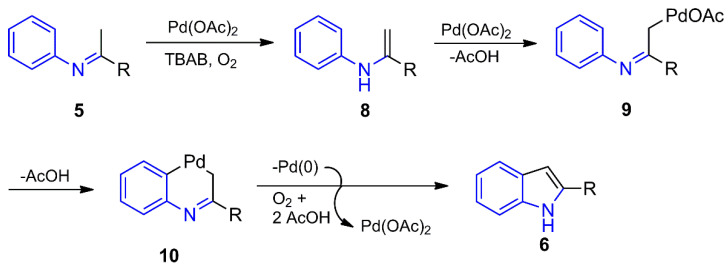
Indole synthesis from imines through a Pd(II)-catalyzed oxidative cross-coupling.

The Pd(II)-catalyzed conversion of imines/enamines into indoles has been performed by fairly similar methods [89,90,91], previously forming imine **5** or generating it in situ as the intermediate [66,67,68] (Scheme 1b). In all the approaches, the mechanism seems to start from the tautomerization of imine **5** to enamine **8**, which undergoes a dehydrogenative reaction by the metal to furnish the σ-Pd(II) complex **9** (Scheme 2). An aryl C–H activation affords palladacycle **10**, bridging the aryl and the methylene moieties. By accomplishing the reductive elimination step, the internal oxidative cross-coupling reaction of **10** promotes the formation of the C-3/C-3a sigma bond of the corresponding indole **6** and Pd(0). The latter is oxidized back to Pd(II) by the oxidizing agent to renew the catalytic cycle. This aerobic oxidative cyclization reaction is only modestly efficient and regioselective in a one-pot reaction beginning from the aniline and the ketone [67]. With an excess of Cu(OAc)_2_, on the other hand, the reaction produces a couple of indoles in moderate yields (41–55%) [66].

Considering the feasibility of the oxidative cyclization reaction from imines **5** to indoles **6**, a one-pot reaction was explored that started from aniline **1i** and phenylacetylene (**2a**) and progressed by a sequential Hg(I)/Pd(II)-catalyzed process. Diverse reagents were tested under aerobic conditions to find the proper oxidizing agent (Table 3). The reaction conditions were standardized and carried out in two steps. The first step consisted of applying the optimal reaction conditions found for the preparation of imines **5**, thus fixing the temperature and reaction time at 40 ºC and 2 h. The second step involved the addition of Pd(OAc)_2_ along with the oxidizing agent in DMSO as the solvent, and then stirring at 60 ºC for 4.5 h.

The synergic action of Cu(OAc)_2_ and oxygen turned out to be the best oxidizing agent (Table 3, entries 8 and 9), although no significant conversion was shown when reacting each separately (entries 1 and 2). The addition of 0.2 mol equivalents of Pd(OAc)_2_ was critical for improving the yield (entry 9). Interestingly, mixing all the catalysts with the oxidant and solvent (DMSO) under similar reaction conditions and as a single step did not provide any trace of the imine or the indole. A possible explanation is that the Hg(I) catalyst was inactivated by DMSO. Indeed, the first step was always inefficient with the use of this solvent (Table 1).

**Scheme 3:**
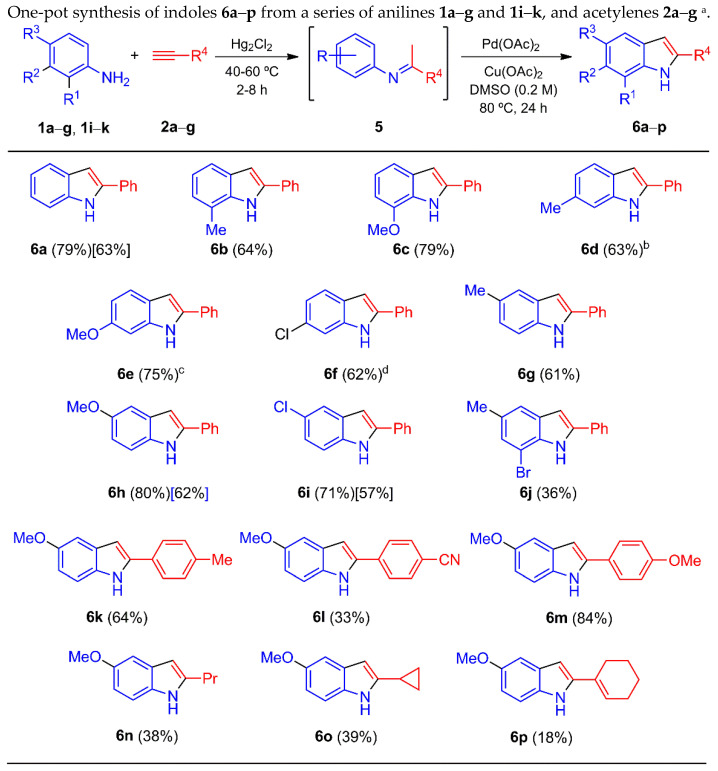
^a^ Yields in square brackets were obtained by conversion of imines **5a** and **5i**–**j** into indoles **6a** and **6h**–**i** (see Experimental Section). ^b^ Minor isomer 4-methyl-2-phenyl-1*H*-indole (**6d’**) was detected (12% ratio) by GC/MS of the crude mixture, but could not be isolated. ^c^ Minor isomer 4-methoxy-2-phenyl-1*H*-indole (**6e’**) was detected (7% ratio) by GC/MS of the crude mixture, but could not be isolated. ^d^ Minor isomer 4-chloro-2-phenyl-1*H*-indole (**6f’**) was detected (19% ratio) by GC/MS of the crude mixture, but could not be isolated.

With the aim of optimizing the process, the additive was changed to K_2_CO_3_ and Cs_2_CO_3_ rather than Li_2_CO_3_, but without success (not shown in Table 3). Hence, the conversion depicted in Table 3 was carried out with or without the addition of Li_2_CO_3_ in the second step. The addition of an acid instead of Li_2_CO_3_ (entries 10–12) caused a non-significant decrease in the yield. A similar outcome was found without the presence of oxygen (entry 13). However, when both the additive and oxygen were removed from the whole process, the yield sharply increased (entry 14). The reaction temperatures and times with the best efficiency were determined, and the optimized method for the elaboration of **6h** was applied to the entire series, mixing anilines **1a**–**g** and **1i**–**k** with acetylenes **2a**–**g**, which gave the corresponding indoles **6a**–**p** (Scheme 3). Most of the 2-arylindoles were obtained in good yields, regardless of the position of the substituent in the benzene ring.

On the other hand, the indoles produced in low yields showed a certain correlation with the imines afforded in fairly modest yields (Table 2). For example, the in-situ formation of imine **5k** delivered indole **6j** in a low yield. The unstable and non-isolated imines **5m** and **5o**–**q**, generated by acetylenes **2c** and **2e**–**g** respectively, provided low yields of their corresponding indoles **6l** and **6n**–**p**. It is likely that the low yield of indole **6l** was due to the low stability of the imine under the two-step reaction conditions, since the corresponding *p*-cyanoacetophenone (**7b**) was isolated in 20% yield. The reaction of aniline **1h** and phenylacetylene (**2a**) resulted in a complex mixture of products. With the use of *meta-*substituted anilines **1d**–**f** with **2a**, a mixture of regioisomers **6d**–**f**/**6d’**–**f’** was found, respectively. The 6-substituted indoles **6d**–**f** were the major isomers, and the 4-substituted indoles **6d’**–**f’** the minor ones. The latter could not be isolated (see caption, Scheme 3).

Analogous second-step reaction conditions (Pd(OAc)_2_/Cu(OAc)_2_, DMSO, 80 ºC, 24 h) were evaluated for the preparation of indoles **6a** and **6h**–**i** from imines **5a** and **5i**–**j**, leading to lower yields than those obtained from the one-pot procedure (Scheme 3, square brackets). This suggests that a possible Hg/Pd transmetalation reaction during the one-pot process may foster the necessary catalytic activity to promote the oxidative cyclization of the in-situ formed imine and provide the indole. Actually, there are many examples of satisfactory palladium-catalyzed cross-coupling reactions involving a transmetalation process with organomercury substrates [80,92,93].

**Figure 3:**
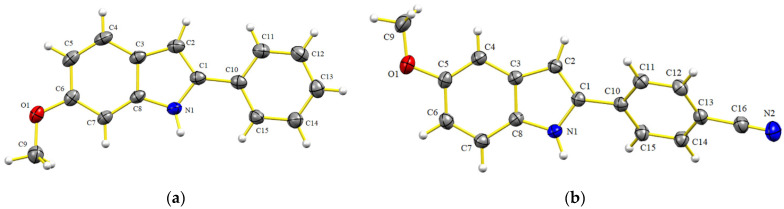
Structures of **6e** (**a**) and **6l** (**b**) as determined by single X-ray diffraction crystallography (ellipsoids at the 30% probability level).

The series of indoles **6a**–**p** was characterized by IR, ^1^H and ^13^C NMR (the signals were attributed by HMQC and HMBC experiments), and HRMS. Indoles **6e** and **6l** were isolated as colorless and pale reddish crystals respectively, and their structures were determined by single X-ray diffraction crystallography (Figure 3). Regarding **6e**, the C-2-substituted benzene ring adopted a coplanar conformation in relation to the heterocycle (torsion angle: N(1)-C(1)-C(10)-C(15) = 1.8(3), see Supplementary Materials). A larger torsion angle can be appreciated for **6l** (torsion angle: C(15)-C(10)-C(1)-N(1) = 32.41(18), see Supplementary Materials). Thus, it is likely that the π-system of the aromatic rings in **6e** is stabilized by conjugation, despite the possible van der Waals repulsions triggered by the eclipsed conformation of the *ortho* C-H protons (C2-H and C11-H). Meanwhile, the coplanarity between the aromatic rings is lost for **6l**, leading to the rotation of the sigma bond C1–C10. This is probably due to the presence of the cyano group, which compensates for the loss of stability caused by the conjugation of the aromatic rings. The latter loss is due to the instability caused by such *ortho* C-H and N-H repulsions.

### 2.3. Mechanism of the Formation of Indoles **6a**–**p**

**Scheme 4:**
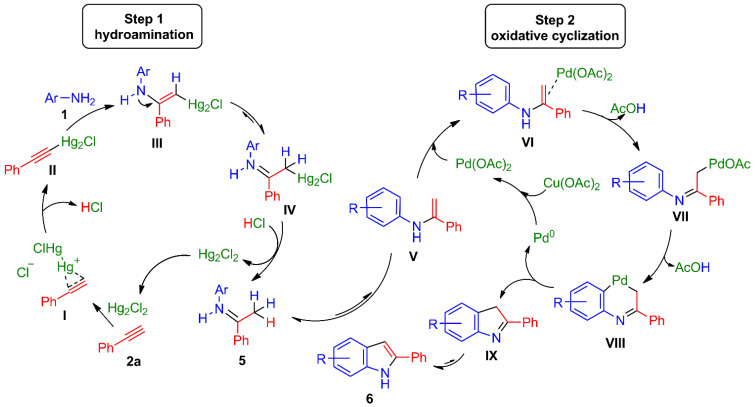
Possible consecutive Hg(I)/Pd(II) catalytic cycles for the one-pot synthesis of indoles **6**. Firstly, hydroamination provides imines **5** (Step 1), and then oxidative cyclization (Step 2) furnishes the indole frame.

Taking into account the aforementioned results as well as the role played by the catalysts through each step [54,66,67,68] in the one-pot process for **2a**, two consecutive catalytic cycles can be suggested (Scheme 4). For the first event, the triple bond of **2a** would be activated by a π-Hg(I) mercurinium cation complex **I**, and then probably stabilized by the formation of the alkynylmercury(I) **II** [83]. The addition of aniline **1** to either of the two possible complexes (**I** or **II**) would generate enamino-vinylmercury(I) **III**, followed by tautomerization to obtain imine **IV**. The protonolysis of the latter [80] would afford imine **5** along with the regeneration of catalyst Hg_2_Cl_2_ to initiate a new catalytic cycle.

The second step would start with the tautomerization of imine **5** to enamine **V**, leading to the π-Pd(II) complex **VI** and the subsequent elimination of AcOH to deliver σ-Pd(II) complex **VII** (Scheme 4) [66,67,68]. Intramolecular aryl C–H activation would furnish palladacycle **VIII**, which would undergo a coupling reaction to give rise to the new C3-C3a sigma bond of heterocycle **IX**. The latter process would be associated with the reductive elimination of Pd(II) to form Pd(0), which would again be oxidized by the action of Cu(OAc)_2_. A final aromatization of **IX** would complete the synthesis of indoles **6**.

Considering the possibility that an Hg/Pd transmetalation reaction takes place to improve the one-pot process with respect to the conversion of imines **5** to indoles **6**, a plausible alternative reaction mechanism can be postulated. The Pd(II)-catalyzed transmetalation reaction may occur either with the vinylmercurial complex **III** [92,93] or the alkylmercurial complex **IV** to directly produce the σ-Pd(II) complex **VII**, which implies a shortcut capable of improving the process and avoiding the in-situ formation of imines **5**.

Since modest efficiency of the mixture of the catalyst and oxidant agent Pd(OAc)_2_/Cu(OAc)_2_ was achieved for the one-pot conversion of anilines and ketones to indoles in only two cases [66], the Hg(I)-free reaction of aniline **1i** with **2a** in the presence of the Pd(OAc)_2_/Cu(OAc)_2_ couple was also tested. However, no indole was found [51]. Indeed, the alkyne was consumed only to generate 1,4-diphenylbuta-1,3-diyne, being the well-known, palladium-catalyzed, self-coupling product of terminal alkynes [94].

Given that Hg(II) was an active catalyst for promoting the formation of imine **5i** (Table 1, entry 9), analogous alkynyl- and vinyl-mercury(II) (i.e., species **I**–**III**) could not be discarded as a competitive complex species [54]. Consequently, an assay was carried out with an HgCl_2_-catalyzed reaction of aniline **1i** with **2a**, followed by the Pd(OAc)_2_/Cu(OAc)_2_ couple-catalyzed cyclization in DMSO, which led to indole **6h** in low yield (12%). This indicates that the mechanism of the reaction described in Scheme 4 is mainly driven by Hg(I) intermediates (i.e., species **I**–**IV**).

## 3. Materials and Methods

### 3.1. General

Melting points were determined on a Krüss KSP 1N (KRÜSS GmbH, Hamburg, Germany) capillary melting point apparatus. IR spectra were recorded on Perkin-Elmer 2000 (PerkinElmer, Waltham, MA, USA) and Bruker Vertex 70 (ATR-FT) (Bruker Corporation, Billerica, MA, USA) spectrophotometers. ^1^H and ^13^C NMR spectra were captured on Varian Mercury (300 MHz) (Varian, Inc., Palo Alto, CA, USA), Varian VNMR (500 MHz) (Varian, Inc., Palo Alto, CA, USA), and Bruker 600 AVANCE III (600 MHz) (Bruker Corporation, Billerica, MA, USA) instruments, with CDCl_3_ as the solvent and TMS as the internal standard. Signal assignments were based on 2D NMR spectra (HMQC, HMBC, and/or ROESY). Mass spectra (MS) were recorded on Thermo Polaris Q-Trace GC Ultra (Finnigan Co., Waltham, MA, USA) and Hewlett-Packard 5971 A (Hewlett Packard Co., Houston, TX, USA) spectrometers. High-resolution mass spectra (HRMS) were obtained (in electron impact mode) on a Jeol JSM-GCMateII (JEOL, Ltd., Tokyo, Japan) spectrometer. X-Ray crystallographic measurements were collected on an Oxford XcaliburS diffractometer (Rigaku Co., Tokyo, Japan). Analytical thin-layer chromatography was performed with 0.25 plates coated with E. Merck (Darmstadt, Germany) silica gel 60 F254 and visualized with a short/long-wavelength UV lamp. Flash column chromatography was conducted over Natland International Co. (Morrisville, NC, USA) silica gel (230–400 and 230–400 mesh). All air moisture-sensitive reactions were conducted under N_2_ in oven-dried glassware. DMSO was distilled over CaH_2_, and Li_2_CO_3_ and K_2_CO_3_ were dried overnight at 250 °C prior to use. All other reagents were utilized without further purification. Warning: The hazardous wastes of mercury-containing silica gel and reaction residues were treated with sulfur and placed in high-density plastic containers for disposal, then handled by an authorized hazardous waste company. 

### 3.2. General Method A for the Preparation of Imines **5a**–**n**

To a round-bottomed flask (at room temperature and in the dark), the corresponding arylacetylene **2a**–**d** (1.0 mol equiv.), Li_2_CO_3_ (0.20 mol equiv.), the respective aniline **1a**–**k** (1.05 mol equiv.), and mercury(I) chloride (0.05 mol equiv.) were successively added. The mixture was vigorously stirred at 60 ºC for 2–8 h. The organic layer was extracted with EtOAc (3 × 5 mL) and dried with Na_2_SO_4_. The solvent was removed under vacuum and the residue was purified by column chromatography over silica gel treated with Et_3_N (10% w/w) (40 g/g crude, hexane/EtOAc, 98:2) to afford imines **5a**–**n**.

### 3.3. General Method B for the Preparation of Indoles **6a–p**

To a round-bottomed flask (at room temperature and in the dark), the corresponding alkyl and arylacetylenes **2a**–**g** (1.0 mol equiv.), the respective aniline **1a**–**g** and **1i**–**k** (1.05 mol equiv.), and mercury(I) chloride (0.05 mol equiv.) were successively added. The mixture was vigorously stirred at 40–60 ºC for 2–8 h, and anhydrous DMSO (0.2 M), Cu(OAc)_2_ (1.5 mol equiv.), and Pd(OAc)_2_ (0.20 mol equiv.) were successively added. The mixture was stirred at 80 ºC for 24 h, filtered over celite, and washed with EtOAc (4 × 20 mL). The organic layer was washed with brine (40 mL) and dried with Na_2_SO_4_. The solvent was removed under vacuum and the residue was purified by column chromatography over silica gel (30 g/g crude, hexane/EtOAc, 98:2) to provide indoles **6a**–**p**.

### 3.4. General Method C for the Preparation of Indoles **6a** and **6h–i** Starting from Imines **5a** and **5i–j**

In a round-bottomed flask (at room temperature), the respective imine **5a** and **5i**–**j**, anhydrous DMSO (0.2 M), Cu(OAc)_2_ (1.5 mol equiv.), and Pd(OAc)_2_ (0.20 mol equiv.) were mixed and stirred at 80 ºC for 24 h. The crude reaction was filtered over celite and washed with EtOAc (4 × 20 mL). The organic layer was washed with brine (40 mL) and dried with Na_2_SO_4_. The solvent was removed under vacuum and the residue was purified by column chromatography over silica gel (50 g/g crude, hexane/EtOAc, 98:2) to obtain indoles **6a** and **6h**–**i**.

### 3.5. Preparation and Characterization of Imines **5a–n**

#### 3.5.1. (*E*)-*N*-(1-Phenylethylidene)aniline (**5a**)

Following general method A, a mixture of **2a** (0.094 g, 0.92 mmol), Li_2_CO_3_ (0.014 g, 0.19 mmol), **1a** (0.090 g, 0.97 mmol), and Hg_2_Cl_2_ (0.022 g, 0.047 mmol) was reacted for 4 h to furnish **5a** (0.159 g, 88%) as a yellow solid. *Rf* 0.72 (hexane/EtOAc, 7:3); mp 35–36 °C (Lit. 37 °C [10]; 42–43 °C [95]). IR (film): ῡ 3058, 3027, 1638, 1593, 1482, 1447, 1366, 1287, 1214, 1072, 1026, 783, 762, 730, 694 cm^−1^. ^1^H NMR (500 MHz, CDCl_3_): δ 2.23 (s, 3H, *CH_3_*-2’), 6.78–6.82 (m, 2H, H-2), 7.06–7.11 (m, 1H, H-4), 7.32–7.38 (m, 2H, H-3), 7.41–7.49 (m, 3H, H-3”, H-4”), 7.95–7.80 (m, 2H, H-2”). ^13^C NMR (125 MHz, CDCl_3_): δ 17.4 (*CH_3_*-2’), 119.3 (C-2), 123.2 (C-4), 127.1 (C-2”), 128.3 (C-3”), 128.9 (C-3), 130.4 (C-4”), 139.5 (C-1”), 151.7 (C-1), 165.4 (C-1′). HRMS (EI): *m/z* [M^+^] calcd for C_14_H_13_N: 195.1048; found: 195.1046.

#### 3.5.2. (*E*)-2-Methyl-*N*-(1-phenylethylidene)aniline (**5b**)

Following general method A, a mixture of **2a** (0.101 g, 0.99 mmol), Li_2_CO_3_ (0.015 g, 0.20 mmol), **1b** (0.111 g, 1.04 mmol), and Hg_2_Cl_2_ (0.023 g, 0.049 mmol) was reacted for 4 h to give **5b** (0.176 g, 85%) as a yellow solid. *Rf* 0.77 (hexane/EtOAc, 7:3); mp 56–58 °C (Lit. 61 °C [10]). IR (film): ῡ 3063, 3016, 1637, 1597, 1578, 1482, 1447, 1366, 1287, 1221, 1114, 1026, 787, 765, 737, 692 cm^−1^. ^1^H NMR (600 MHz, CDCl_3_): δ 2.10 (s, 3H, *CH_3_*Ar), 2.16 (s, 3H, *CH_3_*-2’), 6.65 (br d, *J* = 7.8 Hz, 2H, H-6), 7.00 (br dd, *J* = 7.8, 7.2 Hz, 1H, H-4), 7.17 (br dd, *J* = 7.8, 7.2 Hz, 1H, H-5), 7.21 (br d, *J* = 7.2 Hz, 1H, H-3), 7.43–7.49 (m, 3H, H-3”, H-4”), 7.99–8.02 (m, 2H, H-2”). ^13^C NMR (150 MHz, CDCl_3_): δ 17.4 (*CH_3_*-2’), 17.8 (*CH_3_*Ar), 118.4 (C-6), 123.2 (C-4), 126.3 (C-5), 127.1 (C-2, C-2”), 128.4 (C-3”), 130.3 (C-3), 130.4 (C-4”), 139.4 (C-1”), 150.3 (C-1), 164.9 (C-1’).

#### 3.5.3. (*E*)-2-Methoxy-*N*-(1-phenylethylidene)aniline (**5c**)

Following general method A, a mixture of **2a** (0.107 g, 1.05 mmol), Li_2_CO_3_ (0.015 g, 0.20 mmol), **1c** (0.135 g, 1.10 mmol), and Hg_2_Cl_2_ (0.025 g, 0.053 mmol) was reacted for 4 h to afford **5c** (0.205 g, 87%) as a yellow oil. *Rf* 0.70 (hexane/EtOAc, 7:3). IR (film): ῡ 3059, 3001, 2957, 2834, 1684, 1635, 1590, 1489, 1448, 1366, 1242, 1208, 1180, 1115, 1046, 1026, 765, 746, 693 cm^−1^. ^1^H NMR (500 MHz, CDCl_3_): δ 2.18 (s, 3H, *CH_3_*-2’), 3.79 (s, 3H, *CH_3_*O), 6.78 (dd, *J* = 7.5, 1.7 Hz, 2H, H-6), 6.94 (dd, *J* = 7.5, 1.5 Hz, 1H, H-3), 6.96 (td, *J* = 7.5, 1.5 Hz, 1H, H-5), 7.08 (td, *J* = 7.5, 1.7 Hz, 1H, H-4), 7.42–7.48 (m, 3H, H-3”, H-4”), 7.97–8.03 (m, 2H, H-2”). ^13^C NMR (125 MHz, CDCl_3_): δ 17.8 (*CH_3_*-2’), 55.6 (*CH_3_*O), 111.5 (C-3), 120.5 (C-5), 120.8 (C-6), 124.1 (C-4), 127.3 (C-2”), 128.3 (C-3”), 130.4 (C-4”), 139.3 (C-1”), 140.5 (C-1), 148.9 (C-2), 167.1 (C-1’) [96].

#### 3.5.4. (*E*)-3-Methyl-*N*-(1-phenylethylidene)aniline (**5d**)

Following general method A, a mixture of **2a** (0.096 g, 0.94 mmol), Li_2_CO_3_ (0.014 g, 0.19 mmol), **1d** (0.106 g, 0.99 mmol), and Hg_2_Cl_2_ (0.022 g, 0.047 mmol) was reacted for 4 h to give **5d** (0.163 g, 83%) as a yellow oil. *Rf* 0.67 (hexane/EtOAc, 7:3); (Lit. mp 34 °C [10]). IR (film): ῡ 3059, 3001, 2958, 2834, 1684, 1634, 1594, 1485, 1447, 1367, 1313, 1288, 1261, 1193, 1148, 1045, 913, 852, 784, 765, 694 cm^−1^. ^1^H NMR (500 MHz, CDCl_3_): δ 2.23 (s, 3H, *CH_3_*-2’), 2.35 (s, 3H, *CH_3_*Ar), 6.60 (dm, *J* = 7.5 Hz, 1H, H-6), 6.61–6.64 (m, 1H, H-2), 6.90 (dm, *J* = 7.5 Hz, 1H, H-4), 7.23 (t, *J* = 7.5 Hz, 1H, H-5), 7.41–7.48 (m, 3H, H-3”, H-4”), 7.94–7.98 (m, 2H, H-2”). ^13^C NMR (125 MHz, CDCl_3_): δ 17.3 (*CH_3_*-2’), 21.5 (*CH_3_*Ar), 116.3 (C-6), 120.0 (C-2), 123.9 (C-4), 127.1 (C-2”), 128.3 (C-3”), 128.8 (C-5), 130.4 (C-4”), 138.7 (C-3), 139.5 (C-1”), 151.7 (C-1), 165.2 (C-1’). HRMS (EI): *m/z* [M^+^] calcd for C_15_H_15_N: 209.1204; found: 209.1205.

#### 3.5.5. (*E*)-3-Methoxy-*N*-(1-phenylethylidene)aniline (**5e**)

Following general method A, a mixture of **2a** (0.096 g, 0.94 mmol), Li_2_CO_3_ (0.014 g, 0.19 mmol), **1e** (0.122 g, 0.99 mmol), and Hg_2_Cl_2_ (0.022 g, 0.047 mmol) was reacted for 4 h to form **5e** (0.178 g, 84%) as a yellow oil. *Rf* 0.70 (hexane/EtOAc, 7:3). IR (film): ῡ 3452, 3369, 2929, 2837, 1602, 1496, 1464, 1292, 1206, 1158, 1038, 835, 764, 688. ^1^H NMR (500 MHz, CDCl_3_): δ 2.25 (s, 3H, *CH_3_*-2’), 3.81 (s, 3H, *CH_3_*O), 6.35–6.40 (m, 2H, H-2, H-6), 6.64 (ddd, *J* = 8.0, 2.0, 1.0 Hz, 1H, H-4), 7.25 (t, *J* = 8.0 Hz, 1H, H-5), 7.42–7.49 (m, 3H, H-3”, H-4”), 7.95–7.98 (m, 2H, H-2”). ^13^C NMR (125 MHz, CDCl_3_): δ 17.4 (*CH_3_*-2’), 55.2 (*CH_3_*O), 105.0 (C-2), 108.8 (C-4), 111.7 (C-6), 127.1 (C-2”), 128.3 (C-3”), 129.8 (C-5), 130.5 (C-4”), 139.4 (C-1”), 153.1 (C-1), 160.3 (C-3), 165.5 (C-1’) [97].

#### 3.5.6. (*E*)-3-Chloro-*N*-(1-phenylethylidene)aniline (**5f**)

Following general method A, a mixture of **2a** (0.111 g, 1.09 mmol), Li_2_CO_3_ (0.016 g, 0.22 mmol), **1f** (0.145 g, 1.14 mmol), and Hg_2_Cl_2_ (0.026 g, 0.055 mmol) was reacted for 4 h to provide **5f** (0.132 g, 53%) as a brown oil. *Rf* 0.76 (hexane/EtOAc, 7:3). IR (film): ῡ 3060, 2920, 1635, 1588, 1470, 1448, 1367, 1288, 1212, 1072, 864, 788, 763, 692 cm^−1^. ^1^H NMR (600 MHz, CDCl_3_): δ 2.25 (s, 3H, *CH_3_-*2’), 6.68 (dm, *J* = 8.1 Hz, 1H, H-6), 6.81 (t, *J* = 1.8 Hz, 1H, H-2), 7.07 (dm, *J* = 8.1 Hz, 1H, H-4), 7.27 (t, *J* = 8.1 Hz, 1H, H-5), 7.43–7.51 (m, 3H, H-3”, H-4”), 7.94–7.97 (m, 2H, H-2”). ^13^C NMR (150 MHz, CDCl_3_): δ 17.9 (*CH_3_*-2’), 118.0 (C-6), 119.8 (C-2), 123.5 (C-4), 127.5 (C-2”), 128.7 (C-3”), 130.4 (C-5), 131.1 (C-4”), 134.8 (C-3), 139.3 (C-1”), 152.2 (C-1), 166.6 (C-1’). HRMS (EI): *m/z* [M^+^] calcd for C_14_H_12_N: 229.0658; found: 209.0652 [13].

#### 3.5.7. (*E*)-4-Methyl-*N*-(1-phenylethylidene)aniline (**5g**)

Following general method A, a mixture of **2a** (0.096, 0.94 mmol), Li_2_CO_3_ (0.014 g, 0.19 mmol), **1g** (0.105 g, 0.98 mmol), and Hg_2_Cl_2_ (0.022 g, 0.047 mmol) was reacted for 2 h to afford **5g** (0.179 g, 91%) as a yellow oil. *Rf* 0.76 (hexane/EtOAc, 7:3); (Lit. 62 °C [10]). IR (film): ῡ 3449, 3363, 3023, 2920, 1683, 1633, 1516, 1505, 1447, 1364, 1288, 1266, 1219, 1026, 842, 827, 763, 692 cm^−^^1^. ^1^H NMR (600 MHz, CDCl_3_): δ 2.24 (s, 3H, *CH_3_*-2’), 2.35 (s, 3H, *CH_3_*Ar), 6.70 (d, *J* = 7.8 Hz, 2H, H-2), 7.16 (d, *J* = 7.8 Hz, 2H, H-3), 7.41–7.49 (m, 3H, H-3”, H-4”), 7.94–7.99 (m, 2H, H-2”). ^13^C NMR (150 MHz, CDCl_3_): δ 17.3 (*CH_3_*

-2’), 20.9 (*CH_3_*Ar), 119.4 (C-2), 127.1 (C-2”), 128.3 (C-3”), 129.5 (C-3), 130.3 (C-4”), 132.6 (C-4), 139.7 (C-1”), 149.0 (C-1), 165.5 (C-1’). HRMS (EI): *m/z* [M^+^] calcd for C_15_H_15_N: 209.1204; found: 209.1210 [51].

#### 3.5.8. (*E*)-4-(*N*,*N*-dimethylamino)-*N*-(1-phenylethylidene)aniline (**5h**)

Following general method A, a mixture of **2a** (0.099 g, 0.97 mmol), Li_2_CO_3_ (0.014 g, 0.19 mmol), **1h** (0.139 g, 1.02 mmol) and Hg_2_Cl_2_ (0.023 g, 0.049 mmol) was reacted for 2 h to obtain **5h** (0.158 g, 68%) as a brown solid. *Rf* 0.53 (hexane/EtOAc, 7:3); mp 90–92 °C. IR (film): ῡ 2918, 2803, 1622, 1510, 1446, 1364, 1222, 830, 760, 690 cm^−1^. ^1^H NMR (500 MHz, CDCl_3_): δ 2.28 (s, 3H, *CH_3_*-2’), 2.95 (s, 6H, (*CH_3_*)_2_N), 6.75–6.82 (m, 4H, H-2, H-3), 7.41–7.45 (m, 3H, H-3”, H4”), 7.95–7.97 (m, 2H, H-2”). ^13^C NMR (125 MHz, CDCl_3_): δ 17.3 (*CH_3_*-2’), 41.2 ((*CH_3_*)_2_N), 113.5 (C-3), 121.0 (C-2), 127.0 (C-2”), 128.3 (C-3”), 130.1 (C-4”), 140.1 (C-1”), 141.7 (C-1), 147.4 (C-4), 165.0 (C-1’). HRMS (EI): *m/z* [M^+^] calcd for C_16_H_18_N_2_: 238.1470; found: 238.1471 [98].

#### 3.5.9. (*E*)-4-Methoxy-*N*-(1-phenylethylidene)aniline (**5i**)

Following general method A, a mixture of **2a** (0.125 g, 1.23 mmol), Li_2_CO_3_ (0.018 g, 0.24 mmol), **1i** (0.158 g, 1.28 mmol), and Hg_2_Cl_2_ (0.029 g, 0.06 mmol) was reacted for 2 h to furnish **5i** (0.252 g, 91%) as a pale yellow crystalline solid. *Rf* 0.70 (hexane/EtOAc, 7:3); mp 93–94 °C (Lit. 86 °C [10,95]; Lit. 85–86 °C [50]). IR (KBr): ῡ 2954, 2929, 1616, 1576, 1510, 1443, 1365, 1284, 1240, 1210, 1032, 846, 771, 752, 699 cm^−1^. ^1^H NMR (600 MHz, CDCl_3_): δ 2.26 (s, 3H, *CH_3_*-2’), 3.82 (s, 3H, *CH_3_*O), 6.73–6.79 (m, 2H, H-2), 6.89–6.94 (m, 2H, H-3), 7.41–7.49 (m, 3H, H-3”, H-4”), 7.95–7.98 (m, 2H, H-2”). ^13^C NMR (150 MHz, CDCl_3_): δ 17.3 (C-2’), 55.5 (*CH_3_*O), 114.2 (C-3), 120.7 (C-2), 127.1 (C-2”), 128.3 (C-3”), 130.3 (C-4”), 139.8 (C-1”), 144.8 (C-1), 155.9 (C-4), 165.7 (C-1’). HRMS (EI): *m/z* [M^+^] calcd for C_15_H_15_NO: 225.1154; found: 225.1164 [51,99].

#### 3.5.10. (*E*)-4-Chloro-*N*-(1-phenylethylidene)aniline (**5j**)

Following general method A, a mixture of **2a** (0.119 g, 1.17 mmol), Li_2_CO_3_ (0.017 g, 0.23 mmol), **1j** (0.155 g, 1.22 mmol), and Hg_2_Cl_2_ (0.027 g, 0.057 mmol) was reacted for 2 h to give **5j** (0.209 g, 78%) as a yellow solid. *Rf* 0.76 (hexane/EtOAc, 7:3); mp 68–70 °C (Lit. 73–74 °C [99]). IR (film): ῡ 3061, 2955, 2854, 1634, 1579, 1483, 1447, 1367, 1288, 1214, 1091, 1010, 844, 762, 692, 674 cm^−1^. ^1^H NMR (500 MHz, CDCl_3_): δ 2.23 (s, 3H, *CH_3_*-2’), 6.71–6.76 (m, 2H, H-2), 7.28–7.33 (m, 2H, H-3), 7.42–7.50 (m, 3H, H-3”, H-4”), 7.93–7.98 (m, 2H, H-2”). ^13^C NMR (125 MHz, CDCl_3_): δ 17.4 (*CH_3_*-2’), 120.8 (C-2), 127.2 (C-2”), 128.4 (C-3”), 128.5 (C-4), 129.0 (C-3), 130.7 (C-4”), 139.2 (C-1”), 150.2 (C-1), 166.2 (C-1’) [13,51].

#### 3.5.11. (*E*)-2-Bromo-4-methoxy-*N*-(1-phenylethylidene)aniline (**5k**)

Following general method A, a mixture of **2a** (0.093 g, 0.91 mmol), Li_2_CO_3_ (0.013 g, 0.182 mmol), **1k** (0.177 g, 0.95 mmol), and Hg_2_Cl_2_ (0.021 g, 0.044 mmol) was reacted for 8 h to produce **5k** (0.110 g, 42%) as a yellow oil. *Rf* 0.73 (hexane/EtOAc, 7:3). IR (film): ῡ 3057, 3026, 2921, 1645, 1634, 1578, 1480, 1447, 1366, 1293, 1221, 1044, 865, 828, 761, 692 cm^−1^. ^1^H NMR (500 MHz, CDCl_3_): δ 2.19 (s, 3H, *CH_3_*-2’), 2.33 (s, 3H, *CH_3_*Ar), 6.68 (d, *J* = 8.0 Hz, 1H, H-6), 7.09 (dd, *J* = 8.0, 2.0 Hz, 1H, H-5), 6.43 (d, *J* = 2.0 Hz, 1H, H-3), 7.44–7.50 (m, 3H, H-3”, H-4”), 7.98–8.02 (m, 2H, H-2’). ^13^C NMR (125 MHz, CDCl_3_): δ 18.0 (*CH_3_*-2’), 20.5 (*CH_3_*Ar), 113.5 (C-2), 120.1 (C-6), 127.4 (C-2”), 128.4 (C-3”), 128.7 (C-5), 130.7 (C-4”), 133.1 (C-3), 134.2 (C-4), 139.1 (C-1”), 147.4 (C-1), 167.6 (C-1’). HRMS (EI): *m/z* [M^+^] calcd for C_15_H_14_BrN: 287.0310; found: 287.0318.

#### 3.5.12. (*E*)-4-Methoxy-*N*-(1-(*p*-tolyl)ethylidene)aniline (**5l**)

Following general method A, a mixture of **2b** (0.118 g, 1.02 mmol), Li_2_CO_3_ (0.015 g, 0.20 mmol), **1i** (0.132 g, 1.07 mmol), and Hg_2_Cl_2_ (0.024 g, 0.051 mmol) was reacted for 2 h to generate **5l** (0.221 g, 91%) as a yellow solid. *Rf* 0.73 (hexane/EtOAc, 7:3); mp 78–80 °C (Lit. 83 °C [10]). IR (film): ῡ 2996, 2954, 2930, 2835, 1621, 1576, 1505, 1445, 1366, 1286, 1242, 1211, 1181, 1032, 846, 761, 699 cm^−1^. ^1^H NMR (500 MHz, CDCl_3_): δ 2.22 (s, 3H, *CH_3_*-2’), 2.40 (s, 3H, *CH_3_*Ar), 3.81 (s, 3H, *CH_3_*O), 6.72–6.76 (m, 2H, H-2), 6.88–6.92 (m, 2H, H-3), 7.22–7.25 (m, 2H, H-3”), 7.84–7.87 (m, 2H, H-2”). ^13^C NMR (125 MHz, CDCl_3_): δ 17.2 (*CH_3_*-2’), 21.3 (*CH_3_*Ar), 55.5 (*CH_3_*O), 114.2 (C-3), 120.8 (C-2), 127.1 (C-2”), 129.0 (C-3”), 137.1 (C-1”), 140.5 (C-4”), 145.0 (C-1), 155.8 (C-4), 165.5 (C-1’) [100].

#### 3.5.13. (*E*)-4-(1-((4-methoxyphenyl)imino)ethyl)benzonitrile (**5m**)

Following general method A, a mixture of **2c** (0.083 g, 0.65 mmol), Li_2_CO_3_ (0.010 g, 0.13 mmol), **1i** (0.084 g, 0.68 mmol), and Hg_2_Cl_2_ (0.014 g, 0.03 mmol) was reacted for 2 h, resulting in an inseparable mixture of **5m**/**7b** (95:5) as an instable brown solid. The yield of compound **5m** (0.147 g, 90%) was estimated by ^1^H NMR and GC/MS. *Rf* 0.48 (hexano/AcOEt, 7:3). ^1^H NMR (500 MHz, CDCl_3_): δ 2.28 (s, 3H, *CH_3_*-2’), 3.83 (s, 3H, *CH_3_*O), 6.73–6.78 (m, 2H, H-2), 6.91–6.95 (m, 2H, H-3), 7.72–7.75 (m, 2H, H-3”), 8.04–8.09 (m, 2H, H-2”). Signals attributed to **7b**: 2.65 (s, *CH_3_*-2’), 3.74 (s, *CH_3_*O), 7.74–7.80 (m, H-3”) [100].

#### 3.5.14. (*E*)-4-Methoxy-*N*-(1-(4-methoxyphenyl)ethylidene)aniline (**5n**)

Following general method A, a mixture of **2d** (0.095 g, 0.72 mmol), Li_2_CO_3_ (0.011 g, 0.15 mmol), **1i** (0.093 g, 0.76 mmol), and Hg_2_Cl_2_ (0.017 g, 0.036 mmol) was reacted for 2 h to form **5n** (0.152 g, 83%) as a pale yellow solid. *Rf* 0.57 (hexane/EtOAc, 7:3); mp 130–132 °C (Lit. 132–134 °C [95]). IR (film): ῡ 2968, 2837, 1626, 1601, 1502, 1379, 1286, 1258, 1239, 1207, 1172, 1028, 833 cm^−1^. ^1^H NMR (600 MHz, CDCl_3_): δ 2.22 (s, 3H, *CH_3_*-2’), 3.82 (s, 3H, *CH_3_*O-4), 3.86 (s, 3H, *CH_3_*O-4”), 6.72–6.76 (m, 2H, H-2), 6.88–6.92 (m, 2H, H-3), 6.93–6.96 (m, 2H, H-3”), 7.91–7.95 (m, 2H, H-2”). ^13^C NMR (150 MHz, CDCl_3_): δ 17.1 (C-2’), 55.4 (*CH_3_*O-C4”), 55.5 (*CH_3_*O-C4), 113.6 (C-3”), 114.2 (C-3), 120.9 (C-2), 128.7 (C-2”), 132.5 (C-1”), 145.0 (C-1), 155.8 (C-4), 161.4 (C4”), 164.8 (C-1’) [99,100].

### 3.6. Preparation and Characterization of Indoles **6a–p**

#### 3.6.1. 2-Phenyl-1H-indole (**6a**)

Following general method B, a mixture of **2a** (0.080 g, 0.79 mmol), **1a** (0.077 g, 0.83 mmol), and Hg_2_Cl_2_ (0.019 g, 0.04 mmol) was stirred at 60 °C for 4 h. It was then reacted with DMSO (3.9 mL), Cu(OAc)_2_ (0.215 g, 1.18 mmol), and Pd(OAc)_2_ (0.035 g, 0.16 mmol) to yield **6a** (0.120 g, 79%) as a brown solid. *Rf* 0.77 (hexane/EtOAc, 7:3).

Following general method C, a mixture of **5a** (0.088 g, 0.45 mmol), DMSO (2.2 mL), Cu(OAc)_2_ (0.123 g, 0.68 mmol), and Pd(OAc)_2_ (0.020 g, 0.09 mmol) was reacted to yield **6a** (0.054 g, 63%) as a pale brownish solid. *Rf* 0.77 (hexane/EtOAc, 7:3); mp 189–190 °C (Lit. 189–190 ºC [66], 188–189 ºC [101], 187–188 ºC [102]). IR (ATR): ῡ 3439, 2919, 2853, 1455, 1230, 794, 770, 742, 690 cm^−1^. ^1^H NMR (600 MHz, CDCl_3_): δ 6.83 (dd, *J* = 2.1, 0.9 Hz, 1H, H-3), 7.12 (td, *J* = 7.8, 0.6 Hz, 1H, H-5), 7.19 (td, *J* = 7.8, 1.2 Hz, 1H, H-6), 7.30–7.33 (m, 1H, H-4’), 7.39 (dd, *J* = 7.8, 0.9 Hz, 1H, H-7), 7.41–7.46 (m, 2H, H-3’), 7.63 (dd, *J* = 7.8, 0.9 Hz, 1H, H-4), 7.64–7.67 (m, 2H, H-2’), 8.32 (br s, 1H, N*H*). ^13^C NMR (150 MHz, CDCl_3_): δ 100.0 (C-3), 110.9 (C-7), 120.3 (C-5), 120.7 (C-4), 122.4 (C-6), 125.2 (C-2’), 127.7 (C-4’), 129.0 (C-3’), 129.3 (C-3a), 132.4 (C-1’), 136.8 (C-7a), 137.9 (C-2).

#### 3.6.2. 7-Methyl-2-phenyl-1H-indole (**6b**) 

Following general method B, a mixture of **2a** (0.077 g, 0.57 mmol), **1b** (0.085 g, 0.79 mmol), and Hg_2_Cl_2_ (0.018 g, 0.04 mmol) was stirred at 60 °C for 4 h. It was then reacted with DMSO (3.8 mL), Cu(OAc)_2_ (0.205 g, 1.13 mmol), and Pd(OAc)_2_ (0.034 g, 0.15 mmol) to provide **6b** (0.10 g, 64%) as a brown solid. *Rf* 0.67 (hexane/EtOAc, 7:3); mp 114–116 °C (Lit. 112–118 °C [103]). IR (ATR): ῡ 3450, 1601, 1486, 1449, 1335, 1330, 1299, 804, 770, 737, 690 cm^−1^. ^1^H NMR (500 MHz, CDCl_3_): δ 2.52 (s, 3H, *CH_3_*Ar), 6.81 (d, *J* = 2.5 Hz, 1H, H-3), 6.98 (dm, *J* = 7.5 Hz, 1H, H-6), 7.04 (t, *J* = 7.5 Hz, 1H, H-5), 7.30 (tm, *J* = 7.5 Hz, 1H, H-4’), 7.40–7.44 (m, 2H, H-3’), 7.47 (dm, *J* = 7.5 Hz, 1H, H-4), 7.64–7.68 (m, 2H, H-2’), 8.15 (br s, 1H, N*H*). ^13^C NMR (125 MHz, CDCl_3_): δ 16.7 (*CH_3_*Ar), 100.6 (C-3), 118.4 (C-4), 120.0 (C-7), 120.4 (C-5), 122.9 (C-6), 125.2 (C-2’), 127.6 (C-4’), 128.8 (C-3a), 129.0 (C-3’), 132.5 (C-1’), 136.4 (C-7a), 137.6 (C-2).

#### 3.6.3. 7-Methoxy-2-phenyl-1H-indole (**6c**)

Following general method B, a mixture of **2a** (0.086 g, 0.84 mmol), **1c** (0.110 g, 0.89 mmol), and Hg_2_Cl_2_ (0.020 g, 0.04 mmol) was stirred at 60 °C for 4 h. It was then reacted with DMSO (4.2 mL), Cu(OAc)_2_ (0.230 g, 1.27 mmol), and Pd(OAc)_2_ (0.038 g, 0.17 mmol) to furnish **6c** (0.149 g, 79%) as an amber solid. *Rf* 0.67 (hexane/EtOAc, 7:3); mp 63–64 °C (Lit. 94–95 ºC [66]). IR (ATR): ῡ 3429, 1608, 1581, 1505, 1485, 1453, 1401, 1332, 1316, 1258, 1225, 1097, 798, 772, 753, 730 cm^−1^. ^1^H NMR (600 MHz, CDCl_3_): δ 4.00 (s, 3H, *CH_3_*O), 6.66 (d, *J* = 7.8 Hz, 1H, H-6), 6.81 (d, *J* = 2.4, 1H, H-3), 7.04 (t, *J* = 7.8 Hz, 1H, H-5), 7.24 (d, *J* = 7.8 Hz, 1H, H-4), 7.31–7.34 (m, 1H, H-4’), 7.42–7.46 (m, 2H, H-3’), 7.68–7.70 (m, 2H, H-2’), 8.56 (br s, 1H, N*H*). ^13^C NMR (150 MHz, CDCl_3_): δ 55.3 (*CH_3_*O), 100.2 (C-3), 102.1 (C-6), 113.3 (C-4), 120.5 (C-5), 125.1 (C-2’), 127.3 (C-7a), 127.6 (C-4’), 129.0 (C-3’), 130.4 (C-3a), 132.4 (C-1’), 137.5 (C-2), 145.9 (C-7). HRMS (EI): *m/z* [M^+^] calcd for C_15_H_13_NO: 223.0997; found: 223.0994.

#### 3.6.4. 6-Methyl-2-phenyl-1H-indole (**6d**). 4-Methyl-2-phenyl-1H-indole (**6d’**)

Following general method B, a mixture of **2a** (0.076 g, 0.75 mmol), **1d** (0.084 g, 0.79 mmol), and Hg_2_Cl_2_ (0.019 g, 0.04 mmol) was stirred at 60 °C for 4 h. It was then reacted with DMSO (3.7 mL), Cu(OAc)_2_ (0.204 g, 1.12 mmol), and Pd(OAc)_2_ (0.034 g, 0.15 mmol) to afford a mixture of **6d**/**6d’** (88:12), which was separated to provide **6d** (0.097 g, 63%) as a pale yellow solid. *Rf* 0.70 (hexane/EtOAc, 7:3); mp 186–187 °C (Lit. 189–190 °C [102], 190–192 °C [103]). IR (ATR): ῡ 3429, 1454, 1384, 1350, 1232, 814, 760, 741, 687 cm^−1^. ^1^H NMR (600 MHz, CDCl_3_): δ 2.47 (s, 3H, *CH_3_*Ar), 6.78 (dd, *J* = 2.4, 0.9 Hz, 1H, H-3), 6.96 (dd, *J* = 8.4, 0.9 Hz, 1H, H-5), 7.20 (d, *J* = 0.9 Hz, 1H, H-7), 7.29–7.32 (m, 1H, H-4’), 7.41–7.45 (m, 2H, H-3’), 7.51 (d, *J* = 8.4, 1H, H-4), 7.63–7.66 (m, 2H, H-2’), 8.21 (br s, 1H, N*H*). ^13^C NMR (150 MHz, CDCl_3_): δ 21.8 (*CH_3_*Ar), 99.8 (C-3), 110.8 (C-7), 120.3 (C-4), 122.0 (C-5), 125.0 (C-2’), 127.1 (C-3a), 127.5 (C-4’), 129.0 (C-3’), 132.3 (C-6), 132.6 (C-1’), 137.2 (C-7a), 137.3 (C-2). Data for **6d**: MS (70 eV): *m/z* 207 (M^+^, 100), 206 (60), 178 (5), 130 (7), 77 (18), 44 (15). Data for **6d’**: MS (70 eV): *m/z* 207 (M^+^, 10), 177 (13), 169 (11), 143 (7), 119 (14), 77 (30), 44 (100).

#### 3.6.5. 6-Methoxy-2-phenyl-1H-indole (**6e**). 4-Methoxy-2-phenyl-1*H*-indole (**6e’**)

Following general method B, a mixture of **2a** (0.085 g, 0.83 mmol), **1e** (0.107 g, 0.87 mmol), and Hg_2_Cl_2_ (0.019 g, 0.04 mmol) was stirred at 60 °C for 4 h. It was then reacted with DMSO (4.4 mL), Cu(OAc)_2_ (0.225 g, 1.24 mmol), and Pd(OAc)_2_ (0.038 g, 0.17 mmol), leading to a mixture of **6e**/**6e’** (93:7) [66], which was separated by column chromatography over silica gel (30 g/g crude, hexane/EtOAc, 98:2) to give **6e** (0.138 g, 75%) as a brown solid. *Rf* 0.60 (hexane/EtOAc, 7:3); mp 160–161 °C (Lit. 177–178 °C [66]; 173–176 °C [102]). IR (ATR): ῡ 3396, 1625, 1446, 1257, 1200, 1158, 1016, 823, 756 cm^−1^. ^1^H NMR (600 MHz, CDCl_3_): δ 3.86 (s, 3H, *CH_3_*O), 6.76 (d, *J* = 1.8, 1H, H-3), 6.80 (dd, *J* = 8.4, 2.4 Hz, 1H, H-5), 6.90 (d, *J* = 2.4, 1H, H-7), 7.27–7.31 (m, 1H, H-4’), 7.41–7.44 (m, 2H, H-3’), 7.50 (d, *J* = 8.4, 1H, H-4), 7.60–7.63 (m, 2H, H-2’), 8.24 (br s, 1H, N*H*). ^13^C NMR (150 MHz, CDCl_3_): δ 55.6 (*CH_3_*O), 94.4 (C-7), 99.8, (C-3), 110.2 (C-5), 121.3 (C-4), 123.5 (C-3a), 124.7 (C-2’), 127.2 (C-4’), 129.0 (C-3’), 132.5 (C-1’), 136.8 (C-2), 137.6 (C-7a), 156.7 (C-6). Data for **6e**: MS (70 eV): *m/z* 223 (M^+^, 77), 208 (100), 180 (23), 152 (17), 77 (3). Data for **6e’**: MS (70 eV): *m/z* 223 (M^+^, 100), 208 (87), 180 (17), 127 (15), 89 (23), 77 (13). HRMS (EI): *m/z* [M^+^] calcd for C_15_H_13_NO: 223.0997; found: 223.0991.

#### 3.6.6. 6-Chloro-2-phenyl-1H-indole (**6f**). 4-Chloro-2-phenyl-1*H*-indole (**6f’**) 

Following general method B, a mixture of **2a** (0.079 g, 0.77 mmol), **1f** (0.103 g, 0.81 mmol), and Hg_2_Cl_2_ (0.019 g, 0.04 mmol) was stirred at 60 °C for 4 h. It was then reacted with DMSO (4.3 mL), Cu(OAc)_2_ (0.211 g, 1.16 mmol), and Pd(OAc)_2_ (0.034 g, 0.15 mmol), resulting in a mixture of **6f**/**6f’** (81:19), which was separated by column chromatography over silica gel (30 g/g crude, hexane/EtOAc, 98:2) to obtain **6f** (0.109 g, 62%) as a yellow solid. *Rf* 0.70 (hexane/EtOAc, 7:3); mp 181–182 °C (Lit. 171–173 °C [104]; 182–183 °C [105]). IR (ATR): ῡ 3429, 1453, 1310, 1067, 876, 805, 758, 736, 688 cm^−1^. ^1^H NMR (500 MHz, CDCl_3_): δ 6.79 (d, *J* = 2.0 Hz, 1H, H-3), 7.09 (dd, *J* = 8.5, 1.5 Hz, 1H, H-5), 7.34 (br t, *J* = 7.5 Hz, 1H, H-4’), 7.39 (d, *J* = 1.5 Hz, 1H, H-7), 7.45 (t, *J* = 7.5 Hz, 2H, H-3’), 7.52 (d, *J* = 8.5 Hz, 1H, H-4), 7.65 (d, *J* = 7.5 Hz, 2H, H-2’), 8.32 (br s, 1H, N*H*). ^13^C NMR (125 MHz, CDCl_3_): δ 100.0 (C-3), 110.8 (C-7), 121.0 (C-5), 121.5 (C-4), 125.2 (C-2’), 127.8 (C-3a), 128.0 (C-4’), 128.1 (C-6), 129.1 (C-3’), 131.9 (C-1’), 137.1 (C-7a), 138.6 (C-2). Data for **6f**: MS (70 eV): *m/z* 229 (M^+^+2, 37), 227 (M^+^, 100), 190 (8), 165 (23), 114 (5), 89 (13), 77 (3). Data for **6f’**: MS (70 eV): *m/z* 229 (M^+^+2, 30), 227 (M^+^, 100), 190 (8), 165 (17), 89 (15).

#### 3.6.7. 5-Methyl-2-phenyl-1*H*-indole (**6g**)

Following general method B, a mixture of **2a** (0.083 g, 0.81 mmol), **1g** (0.092 g, 0.86 mmol), and Hg_2_Cl_2_ (0.019 g, 0.04 mmol) was stirred at 60 °C for 2 h. It was then reacted with DMSO (4.1 mL), Cu(OAc)_2_ (0.222 g, 1.22 mmol), and Pd(OAc)_2_ (0.036 g, 0.16 mmol) to produce **6g** (0.103 g, 61%) as a pale-yellow solid. *Rf* 0.70 (hexane/EtOAc, 7:3); mp 214–215 °C (Lit. 216–218 °C [66]; 211–213 °C [102]; 218–220 °C [103]). IR (ATR): ῡ 3405, 1603, 1449, 1406, 1299, 1202, 1079, 804, 799, 737, 761, 686 cm^−1^. ^1^H NMR (600 MHz, CDCl_3_): δ 2.45 (s, 3H, *CH_3_*), 6.75 (dd, *J* = 2.4, 0.6 Hz, 1H, H-3), 7.02 (dd, *J* = 8.4, 1.2 Hz, 1H, H-6), 7.28 (d, *J* = 8.4 Hz, 1H, H-7), 7.29–7.32 (m, 1H, H-4’), 7.41 (dd, *J* = 1.2, 0.6 Hz, 1H, H-4), 7.41–7.44 (m, 2H, H-3’), 7.63–7.66 (m, 2H, H-2’), 8.23 (br s, 1H, N*H*). ^13^C NMR (150 MHz, CDCl_3_): δ 21.5 (*CH_3_*), 99.5 (C-3), 110.5 (C-7), 120.3 (C-4), 124.0 (C-6), 125.0 (C-2’), 127.6 (C-4’), 129.0 (C-3’), 129.46 (C-5), 129.54 (C-3a), 132.5 (C-1’), 135.2 (C-7a), 137.9 (C-2).

#### 3.6.8. 5-Methoxy-2-phenyl-1*H*-indole (**6h**)

Following general method B, a mixture of **2a** (0.083 g, 0.82 mmol), **1i** (0.106 g, 0.86 mmol), and Hg_2_Cl_2_ (0.019 g, 0.04 mmol) was stirred at 60 °C for 2 h. It was then reacted with DMSO (4.1 mL), Cu(OAc)_2_ (0.222 g, 1.22 mmol), and Pd(OAc)_2_ (0.036 g, 0.16 mmol) to generate **6h** (0.146 g, 80%) as a yellow solid. *Rf* 0.60 (hexane/EtOAc, 7:3).

Following general method C, a mixture of **5i** (0.060 g, 0.27 mmol), DMSO (1.33 mL), Cu(OAc)_2_ (0.073 g, 0.40 mmol), and Pd(OAc)_2_ (0.012 g, 0.054 mmol) was reacted to afford **6h** (0.037 g, 62%) as a pale brownish solid. *Rf* 0.60 (hexane/EtOAc, 7:3). mp 168–169 °C (Lit. 169–170 °C [66], 166–169 °C [101], 160–162 °C [102]). IR (KBr): ῡ 3415, 1615, 1479, 1208, 1148, 1030, 765 cm^−1^. ^1^H NMR (600 MHz, CDCl_3_): δ 3.87 (s, 3H, *CH_3_*O), 6.76 (dd, *J* = 2.4, 1.2 Hz, 1H, H-3), 6.86 (dd, *J* = 9.0, 2.4 Hz, 1H, H-6), 7.09 (d, *J* = 2.4 Hz, 1H, H-4), 7.29 (d, *J* = 9.0 Hz, 1H, H-7), 7.30–7.33 (m, 1H, H-4’), 7.41–7.46 (m, 2H, H-3’), 7.63–7.66 (m, 2H, H-2’), 8.21 (br s, 1H, N*H*). ^13^C NMR (150 MHz, CDCl_3_): δ 55.8 (*CH_3_*O), 99.9 (C-3), 102.3 (C-4), 111.6 (C-7), 112.6 (C-6), 125.1 (C-2’), 127.6 (C-4’), 129.0 (C-3’), 129.7 (C-3a), 132.0 (C-7a), 132.5 (C-1’), 138.6 (C-2), 154.5 (C-5).

#### 3.6.9. 5-Chloro-2-phenyl-1*H*-indole (**6i**)

Following general method B, a mixture of **2a** (0.085 g, 0.83 mmol), **1j** (0.111 g, 0.87 mmol), and Hg_2_Cl_2_ (0.019 g, 0.04 mmol) was stirred at 60 °C for 2 h. It was then reacted with DMSO (4.1 mL), Cu(OAc)_2_ (0.225 g, 1.24 mmol), and Pd(OAc)_2_ (0.037 g, 0.17 mmol) to give **6i** (0.134 g, 71%) as a yellow solid. *Rf* 0.60 (hexane/EtOAc, 7:3).

Following general method C, a mixture of **5j** (0.081 g, 0.35 mmol), DMSO (1.77 mL), Cu(OAc)_2_ (0.073 g, 0.53 mmol), and Pd(OAc)_2_ (0.016 g, 0.07 mmol) was reacted to provide **6i** (0.046 g, 57%) as a yellow solid. *Rf* 0.60 (hexane/EtOAc, 7:3); mp 195–196 °C (Lit. 196–197 °C [66], 198–200 °C [103]). IR (ATR): ῡ 3429, 1452, 1313, 1065, 876, 803, 754, 736, 687 cm^−1^. ^1^H NMR (600 MHz, CDCl_3_): δ 6.76 (d, *J* = 1.8 Hz, 1H, H-3), 7.14 (dd, *J* = 8.4, 1.8 Hz, 1H, H-6), 7.31 (d, *J* = 8.4 Hz, 1H, H-7), 7.35 (t, *J* = 7.5 Hz, 1H, H-4’), 7.45 (t, *J* = 7.5 Hz, 2H, H-3’), 7.59 (d, *J* = 1.8 Hz, 1H, H-4), 7.65 (d, *J* = 7.5 Hz, 2H, H-2’), 8.35 (br s, 1H, N*H*). ^13^C NMR (150 MHz, CDCl_3_): δ 99.6 (C-3), 111.8 (C-7), 120.0 (C-4), 122.6 (C-6), 125.2 (C-2’), 125.9 (C-5), 128.1 (C-4’), 129.1 (C-3’), 130.3 (C-3a), 131.9 (C-1’), 135.1 (C-7a), 139.3 (C-2).

#### 3.6.10. 7-Bromo-5-methyl-2-phenyl-1*H*-indole (**6j**) 

Following general method B, a mixture of **2a** (0.085 g, 0.83 mmol), **1k** (0.163 g, 0.88 mmol), and Hg_2_Cl_2_ (0.020 g, 0.04 mmol) was stirred at 60 °C for 8 h. It was then reacted with DMSO (4.2 mL), Cu(OAc)_2_ (0.227 g, 1.25 mmol), and Pd(OAc)_2_ (0.037 g, 0.17 mmol) to obtain **6j** (0.085 g, 36%) as a brown solid. *Rf* 0.73 (hexane/EtOAc, 7:3); mp 75–77 °C (Lit. 130–131 °C [106]). IR (film): ῡ 3446, 3036, 2918, 1605, 1568, 1477, 1449, 1395, 1310, 1214, 856, 840, 759, 734, 690 cm^−1^. ^1^H NMR (500 MHz, CDCl_3_): δ 2.42 (s, 3H, *CH_3_*), 6.78 (dd, *J* = 2.5, 0.6 Hz, 1H, H-3), 7.18 (br s, 1H, H-6), 7.32–7.36 (m, 1H, H-4, H-4’), 7.42–7.47 (m, 2H, H-3’), 7.65–7.68 (m, 2H, H-2’), 8.32 (br s, 1H, N*H*). ^13^C NMR (125 MHz, CDCl_3_): δ 21.2 (*CH_3_*), 100.5 (C-3), 103.9 (C-7), 119.5 (C-4), 125.3 (C-2’), 125.9 (C-6), 128.0 (C-4’), 129.0 (C-3’), 130.4 (C-3a), 131.1 (C-5), 131.9 (C-1’), 133.8 (C-7a), 138.6 (C-2). HRMS (EI): *m/z* [M^+^] calcd for C_15_H_12_NBr: 285.0153; found: 285.0160.

#### 3.6.11. 5-Methoxy-2-(p-tolyl)-1*H*-indole (**6k**)

Following general method B, a mixture of **2b** (0.094 g, 0.81 mmol), **1i** (0.105 g, 0.85 mmol), and Hg_2_Cl_2_ (0.019 g, 0.04 mmol) was stirred at 60 °C for 2 h. It was then reacted with DMSO (4.1 mL), Cu(OAc)_2_ (0.222 g, 1.22 mmol), and Pd(OAc)_2_ (0.036 g, 0.16 mmol) to give **6k** (0.122 g, 64%) as a yellow solid. *Rf* 0.70 (hexane/EtOAc, 7:3); mp 184–186 °C (Lit. 186–187 °C [66]). IR (ATR): ῡ 3424, 1621, 1543, 1479, 1452, 1301, 1217, 1153, 1115, 1029, 823, 789 cm^−1^. ^1^H NMR (500 MHz, CDCl_3_): δ 2.39 (s, 3H, *CH_3_*Ar), 3.86 (s, 3H, *CH_3_*O), 6.71 (dd, *J* = 2.5, 1.0 Hz, 1H, H-3), 6.84 (dd, *J* = 8.5, 2.5 Hz, 1H, H-6), 7.08 (d, *J* = 2.5 Hz, 1H, H-4), 7.21–7.25 (m, 2H, H-3’), 7.26 (d, *J* = 8.5 Hz, 1H, H-7), 7.51–7.55 (m, 2H, H-2’), 8.17 (br s, 1H, N*H*). ^13^C NMR (125 MHz, CDCl_3_): δ 21.2 (*CH_3_*Ar), 55.8 (*CH_3_*O), 99.3 (C-3), 102.2 (C-4), 111.5 (C-7), 112.3 (C-6), 125.0 (C-2’), 129.6 (C-1’ or C-3a), 129.7 (C-3’), 129.8 (C-3a or C-1’), 131.9 (C-7a), 137.6 (C-4’), 138.8 (C-2), 154.5 (C-5).

#### 3.6.12. 2-(4-Cyanophenyl)-5-methoxy-1*H*-indole (**6l**) 

Following general method B, a mixture of **2c** (0.078 g, 0.61 mmol), **1i** (0.079 g, 0.64 mmol), and Hg_2_Cl_2_ (0.014 g, 0.03 mmol) was stirred at 60 °C for 2 h. It was then reacted with DMSO (3.1 mL), Cu(OAc)_2_ (0.167 g, 0.92 mmol), and Pd(OAc)_2_ (0.027 g, 0.12 mmol) to produce **6l** (0.050 g, 33%) as yellow crystals. *Rf* 0.47 (hexane/EtOAc, 7:3); mp 191–192 °C (Lit. 195–196 °C [66]). IR (KBr): ῡ 3377, 2219, 1606, 1445, 1209, 1030, 836, 789 cm^−1^. ^1^H NMR (500 MHz, CDCl_3_): δ 3.87 (s, 3H, *CH_3_*O), 6.88 (dd, *J* = 1.5, 0.5 Hz, 1H, H-3), 6.92 (dd, *J* = 8.7, 2.5 Hz, 1H, H-6), 7.09 (d, *J* = 2.5 Hz, 1H, H-4), 7.31 (dd, *J* = 8.7, 0.5 Hz, 1H, H-7), 7.69–7.73 (m, 4H, H-2’, H-3’), 8.30 (br s, 1H, N*H*). ^13^C NMR (125 MHz, CDCl_3_): δ 55.8 (*CH_3_*O) 102.3 (C-4), 102.4 (C-3), 110.5 (C-4’), 112.0 (C-7), 114.3 (C-6), 118.8 (CN), 125.1 (C-2’), 129.4 (C-3a), 132.6 (C-7a), 132.8 (C-3’), 136.1 (C-2), 136.6 (C-1’), 154.8 (C-5). HRMS (EI): *m/z* [M^+^] calcd for C_16_H_12_N_2_O: 248.0950; found: 248.0943. 

#### 3.6.13. 5-Methoxy-2-(4-methoxyphenyl)-1*H*-indole (**6m**)

Following general method B, a mixture of **2d** (0.075 g, 0.57 mmol), **1i** (0.074 g, 0.60 mmol), and Hg_2_Cl_2_ (0.014 g, 0.03 mmol) was stirred at 60 °C for 2 h. It was then reacted with DMSO (3.1 mL), Cu(OAc)_2_ (0.156 g, 0.86 mmol), and Pd(OAc)_2_ (0.025 g, 0.11 mmol) to generate **6m** (0.122 g, 84%) as a colorless crystalline solid. *Rf* 0.53 (hexane/EtOAc, 7:3); mp 218–219 °C (Lit. 218–219 °C [66]). IR (ATR): ῡ 3430, 1543, 1480, 1456, 1254, 1217, 1155, 1018, 829, 777 cm^−1^. ^1^H NMR (600 MHz, CDCl_3_): δ 3.85 (s, 3H, *CH_3_*O-C4’), 3.86 (s, 3H, *CH_3_*O-C5), 6.64 (d, *J* = 1.2 Hz, 1H, H-3), 6.83 (dd, *J* = 8.4, 2.4 Hz, 1H, H-6), 6.96–6.99 (m, 2H, H-3’), 7.10 (d, *J* = 2.4 Hz, 1H, H-4), 7.27 (d, *J* = 8.4 Hz, 1H, H-7), 7.56–7.59 (m, 2H, H-2’), 8.13 (br s, 1H, N*H*). ^13^C NMR (150 MHz, CDCl_3_): δ 55.4 (*CH_3_*O), 55.9 (*CH_3_*O), 98.7 (C-3), 102.2 (C-4), 111.4 (C-7), 112.0 (C-6), 114.5 (C-3’), 125.3 (C-1’), 126.4 (C-2’), 129.9 (C-3a), 131.8 (C-7a), 138.7 (C-2), 154.5 (C-5), 159.3 (C-4’).

#### 3.6.14. 5-Methoxy-2-propyl-1*H*-indole (**6n**) 

Following general method B, a mixture of **2e** (0.088 g, 1.29 mmol), **1i** (0.166 g, 1.35 mmol), and Hg_2_Cl_2_ (0.028 g, 0.06 mmol) was stirred at 60 °C for 2 h. It was then reacted with DMSO (6.5 mL), Cu(OAc)_2_ (0.352 g, 1.94 mmol), and Pd(OAc)_2_ (0.058 g, 0.26 mmol) to form **6n** (0.093 g, 38%) as an amber solid. *Rf* 0.67 (hexane/EtOAc, 7:3); p.f. 55–56 °C (Lit. 64–65 °C [107]). IR (ATR): ῡ 3381, 2961, 1620, 1582, 1478, 1450, 1195, 1166, 1034, 831 cm^−1^. ^1^H NMR (500 MHz, CDCl_3_): δ 1.00 (t, *J* = 7.5 Hz, 3H, H-3’), 1.73 (hex, *J* = 7.5 Hz, 2H, H-2’), 2.70 (t, *J* = 7.5 Hz, 2H, H-1’), 3.84 (s, 3H, *CH_3_*O), 6.17 (dd, *J* = 2.0, 1.0 Hz, 1H, H-3), 6.77 (dd, *J* = 8.5, 2.5 Hz, 1H, H-6), 7.00 (d, *J* = 2.5 Hz, H-4), 7.18 (d, *J* = 8.5 Hz, 1H, H-7), 7.74 (br s, 1H, N*H*). ^13^C NMR (125 MHz, CDCl_3_): δ 13.9 (C-3’), 22.5 (C-2’), 30.4 (C-1’), 55.9 (*CH_3_*O), 99.5 (C-3), 102.0 (C-4), 110.7 (C-6), 110.9 (C-7), 129.3 (C-3a), 130.9 (C-7a), 140.7 (C-2), 154.1 (C-5). HRMS (EI): *m/z* [M^+^] calcd for C_12_H_15_NO: 189.1154; found: 189.1153.

#### 3.6.15. 2-Cyclopropyl-5-methoxy-1*H*-indole (**6o**)

Following general method B, a mixture of **2f** (0.097 g, 1.47 mmol), **1i** (0.189 g, 1.54 mmol), and Hg_2_Cl_2_ (0.033 g, 0.07 mmol) was stirred at 40 °C for 2 h. It was then reacted with DMSO (7.3 mL), Cu(OAc)_2_ (0.401 g, 2.21 mmol), and Pd(OAc)_2_ (0.065 g, 0.29 mmol) to provide **6o** (0.107 g, 39%) as a yellow solid. *Rf* 0.67 (hexane/EtOAc, 7:3); mp 57–58 °C. IR (ATR): ῡ 3382, 2914, 1621, 1583, 1482, 1451, 1332, 1214, 1163, 1030, 837, 799, 770, 676 cm^−1^. ^1^H NMR (600 MHz, CDCl_3_): δ 0.73–0.76 (m, 2H, C*H*_2_), 0.91–0.95 (m, 2H, C*H*_2_), 1.87–1.93 (m, 1H, H-1’), 6.07 (dd, *J* = 1.2, 0.6 Hz, 1H, H-3), 6.76 (dd, *J* = 9.0, 2.4 Hz, 1H, H-6), 6.98 (d, *J* = 2.4 Hz, 1H, H-4), 7.13 (d, *J* = 9.0 Hz, 1H, H-7), 7.83 (br s, 1H, N*H*). ^13^C NMR (150 MHz, CDCl_3_): δ 7.3 (2CH_2_), 8.9 (C-1’), 55.8 (*CH_3_*O), 97.5 (C-3), 101.9 (C-4), 110.7 (C-6), 110.8 (C-7), 129.1 (C-3a), 130.8 (C-7a), 142.6 (C-2), 154.1 (C-5). HRMS (EI): *m/z* [M^+^] calcd for C_12_H_13_NO: 187.0997; found: 187.0997 [66].

#### 3.6.16. 2-(Cyclohex-1-en-1-yl)-5-methoxy-1*H*-indole (**6p**) 

Following general method B, a mixture of **2g** (0.096 g, 0.91 mmol), **1i** (0.117 g, 0.95 mmol), and Hg_2_Cl_2_ (0.024 g, 0.05 mmol) was stirred at 40 °C for 2 h. It was then reacted with DMSO (4.5 mL), Cu(OAc)_2_ (0.247 g, 1.36 mmol), and Pd(OAc)_2_ (0.040 g, 0.18 mmol) to yield **6p** (0.037 g 18%) as a brown solid. *Rf* 0.70 (hexane/EtOAc, 7:3); mp 108–110 °C. IR (KBr): ῡ 3429, 2929, 1620, 1586, 1483, 1452, 1414, 1210, 1147, 1033, 837, 802, 782 cm^−1^. ^1^H NMR (600 MHz, CDCl_3_): δ 1.66–1.71 (m, 2H, H-4’), 1.76–1.81 (m, 2H, H-5’), 2.21–2.25 (m, 2H, H-3’), 2.42–2.46 (m, 2H, H-6’), 6.09–6.11 (m, 1H, H-2’), 6.37 (d, *J* = 1.8 Hz, H-3), 6.80 (dd, *J* = 8.6, 2.4 Hz, 1H, H-6), 7.02 (d, *J* = 2.4 Hz, 1H, H-4), 7.18 (d, *J* = 8.6 Hz, 1H, H-7), 7.99 (br s, 1H, N*H*). ^13^C NMR (150 MHz, CDCl_3_): δ 22.2 (C-4’), 22.5 (C-5’), 25.5 (C-3’), 26.0 (C-6’), 55.8 (*CH_3_*O), 98.5 (C-3), 102.2 (C-4), 111.0 (C-7), 111.9 (C-6), 122.5 (C-2’), 129.1 (C-1’), 129.3 (C-3a), 131.3 (C-7a), 140.3 (C-2), 154.1 (C-5). HRMS (EI): *m/z* [M^+^] calcd for C_15_H_17_NO: 227.1310; found: 227.1306.

### 3.7. Single-Crystal X-ray Crystallography

Compounds **5i** (yellow crystals, hexane/EtOAc, 98:2), **6e** (colorless crystals, hexane/EtOAc, 98:2), and **6l** (pale reddish crystals, hexane/EtOAc, 94:6) were prepared and mounted on glass fibers. Crystallographic measurements were performed with an area-detector with Mo Kα diffraction radiation (λ = 71,073 Å; graphite monochromator) at room temperature (Table 4). Unit cell parameters were obtained from a least-squares refinement. Intensities were corrected for Lorentz and polarization effects. Multi-scan absorption correction was applied. Anisotropic temperature factors were introduced for all non-hydrogen atoms. Hydrogen atoms were placed in accordance with electron density maps and idealized positions, and their atomic coordinates were refined by utilizing unit weights. After the structure was solved using SHELXT [108,109], it was implemented in WinGX [110], refined with SHELXL [111], and then visualized and plotted with the MERCURY program package [112].

## 4. Conclusions

A new Hg(I)-catalyzed hydroamination of terminal acetylenes **2a**–**d** in the presence of anilines **1a**–**k** generated a series of ketimines **5a**–**n**. An efficient consecutive Hg(I)/Pd(II)-catalyzed one-pot process led to the direct conversion of **1** and **2** into a series of 2-substituted indoles **6a**–**p**. Although good yields were found for most of the 2-arylindoles, the yields were fairly modest for halogenated, 2-alkyl, and 2-vinyl indoles. The latter limitation was possibly due to the low stability of the imine intermediate under these reaction conditions. Likewise, the direct Hg(I)-catalyzed preparation of the imines either provided low yields or made it impossible to avoid decomposition or hydrolysis to their corresponding acetophenones upon isolation from the reaction mixture. The reaction mechanism of this transformation was explored, finding that an Hg(I)–Pd(II) transmetalation step plausibly connects the consecutive cycles of Hg(I)-catalyzed enamine formation and Pd(II)-catalyzed oxidative cyclization.

## Figures and Tables

**Table 1:**
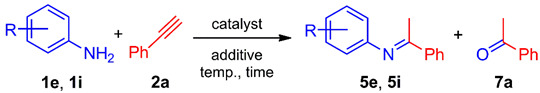
Optimization of the preparation of *N*-arylimines **5e** and **5i** ^a^.

Entry	1 (Ar)	Catalyst	Additive (mol equiv.)	T (°C)	T (h)	5 (Yield, %) ^b^
1	**1e** (C_6_H_4_-3-OMe)	Hg_2_Cl_2_	––	20	24	**5e** (99) ^c^
2	**1i** (C_6_H_4_-4-OMe)	Hg_2_Cl_2_	––	20	24	**5i** (61) ^d^
3	**1i** (C_6_H_4_-4-OMe)	Hg_2_Cl_2_	––	20	48	**5i** (82) ^d^
4	**1i** (C_6_H_4_-4-OMe)	Hg_2_Cl_2_	––	45	4	**5i** (87) ^d^
5	**1e** (C_6_H_4_-3-OMe)	Ag_2_CO_3_	––	20	72	**5e** (0) ^d^
6	**1i** (C_6_H_4_-4-OMe)	Hg_2_Cl_2_	––	60	2	**5i** (72)
7 ^e^	**1i** (C_6_H_4_-4-OMe)	Hg_2_Cl_2_	Li_2_CO_3_ (0.2)	60	2	**5i** (91)
8 ^e^	**1i** (C_6_H_4_-4-OMe)	Hg_2_Cl_2_	(^f^)	60	2	**5i** (70)
9 ^e^	**1i** (C_6_H_4_-4-OMe)	HgCl_2_	Li_2_CO_3_ (0.2)	60	2	**5i** (78)
10 ^e^	**1i** (C_6_H_4_-4-OMe)	Hg^0^	Li_2_CO_3_ (0.2)	60	2	**5i** (0)

^a^**2a** (1.0 mol equiv.), ArNH_2_ (1.0–1.1 mol equiv.), catalyst (10 mol%), and no solvent. ^b^ After purification by column chromatography. ^c^ [85]. ^d^ Calculated by ^1^H NMR of the reaction crude mixture. ^e^ Catalyst (5.0 mol%). ^f^ Hydroquinone (0.5 mol equiv.).

**Table 2:**
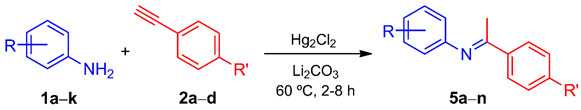
Preparation of the series of *N*-arylimines **5a**–**n** through the Hg(I)-catalyzed hydroamination of **2a**–**d** with anilines **1a**–**k** ^a^.

Entry	1	2 (R’)	R	T (h)	5 (Yield, %) ^b^
1	1a	**2a** (H)	H	4	**5a** (88)
2	1b	**2a** (H)	2-Me	4	**5b** (85)
3	1c	**2a** (H)	2-OMe	4	**5c** (87)
4	1d	**2a** (H)	3-Me	4	**5d** (83)
5	1e	**2a** (H)	3-OMe	4	**5e** (84)
6	1f	**2a** (H)	3-Cl	4	**5f** (53)
7	1g	**2a** (H)	4-Me	2	**5g** (91)
8	1h	**2a** (H)	4-NMe_2_	2	**5h** (68)
9	1i	**2a** (H)	4-OMe	2	**5i** (91)
10	1j	**2a** (H)	4-Cl	2	**5j** (78)
11	1k	**2a** (H)	2-Br-4-Me	8	5k (42)
12	1i	**2b** (Me)	4-OMe	2	5l (91)
13	1i	**2c** (CN)	4-OMe	2	**5m** (90) ^c^
14	1i	**2d** (OMe)	4-OMe	2	5n (83)

^a^ 2 (1.0 mol equiv.), ArNH_2_ (1.0–1.1 mol equiv.), Hg_2_Cl_2_ (5 mol%), Li_2_CO_3_ (0.2 mol equiv.), and no solvent. ^b^ After purification by column chromatography. ^c^ The yield was estimated from the ^1^H NMR spectrum of the crude reaction mixture, resulting in the ratio products, which were then confirmed by the ratio and molecular ions by MS/GC analysis. The product was not isolated and used without purification in the next step.

**Table 3:**
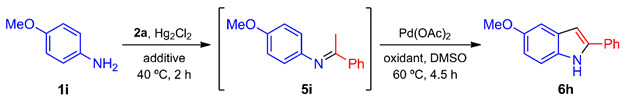
Selection of the oxidizing agent and optimization of the reaction conditions for the one-pot conversion of **1i** and **2a** into indole **6h** ^a^.

Entry	Oxidant (mol equiv.) ^b^	Additive (mol equiv.)	5i/6h (Ratio) ^c^ (Yield, %) ^d^
1	Cu(OAc)_2_ (3.0)	Li_2_CO_3_ (0.2)	78:22
2	O_2_	Li_2_CO_3_ (0.2)	85:15
3	Cu(OAc)_2_/O_2_ (3.0)	Li_2_CO_3_ (0.2)	46:54
4	KMnO_4_/O_2_ (3.0)	Li_2_CO_3_ (0.2)	56:44
5	NMMO/O_2_ (3.0)	Li_2_CO_3_ (0.2)	67:33
6	DTBP/O_2_ (3.0)	Li_2_CO_3_ (0.2)	65:35
7	Cu(OTf)_2_/O_2_ (3.0)	Li_2_CO_3_ (0.2)	100:0
8	Cu(OAc)_2_/O_2_ (1.0)	Li_2_CO_3_ (0.2)	53:47
9 ^e^	Cu(OAc)_2_/O_2_ (3.0)	Li_2_CO_3_ (0.2)	13:87
10 ^e^	Cu(OAc)_2_/O_2_ (3.0)	Li_2_CO_3_ (0.2)	**6h** (45)
11 ^e^	Cu(OAc)_2_/O_2_ (3.0)	––	**6h** (50)
12 ^e,f^	Cu(OAc)_2_/O_2_ (3.0)	EtCO_2_H (0.2)	**6h** (46)
13 ^e,f^	Cu(OAc)_2_ (3.0)	Li_2_CO_3_ (0.2)	**6h** (52)
14 ^e^	Cu(OAc)_2_ (3.0)	––	**6h** (80)

^a^**1i** (1.05 mol equiv.), **2a** (1.0 mol equiv.), Li_2_CO_3_ (0.2 mol equiv.), Hg_2_Cl_2_ (0.05 mol equiv.), Pd(OAc)_2_ (0.1 mol equiv.), and DMSO (0.2 M). ^b^ Oxygen gas was used. ^c^ Calculated by ^1^H NMR of the reaction crude mixture. ^d^ After purification by column chromatography. ^e^ Pd(OAc)_2_ (0.2 mol equiv.) was employed. ^f^ The additive was added at the second step. NMMO = *N-*methylmorpholine *N*-oxide, DTBP = di-*tert*-butyl peroxide.

**Table 4:** Crystal data and structure refinement for **5i, 6e,** and **6l**.

Structure	5i	6e	6l
CCDC number	2082632	2082633	2082634
Empirical formula	C_15_H_15_NO	C_15_H_13_NO	C_16_H_12_N_2_O
Molecular weight	225.28	223.26	248.28
Temperature	292(2) K	292(2) K	292(2) K
Crystal size	0.79 × 0.47 × 0.27 mm^3^	0.50 × 0.30 × 0.21 mm^3^	0.52 × 0.30 × 0.27 mm^3^
Crystal system	monoclinic	orthorhombic	monoclinic
Space group	P2_1_/n	P2_1_2_1_2_1_	*P*2_1_/c
Unit cell parameters	*a* = 10.0110(6) Å, *α* = 90º *b* = 8.7430(5) Å, *β* = 104.172(7) º*c* = 14.6687(11) Å, *γ* = 90º	*a* = 5.6782(3) Å, *α* = 90º *b* = 8.0462(3) Å, *β* = 90º *c* = 25.5690(16) Å, *γ* = 90º	*a* = 7.0104(3) Å, *α* = 90º *b* = 14.5242(8) Å, *β* = 98.202(4)º *c* = 12.5032(7) Å, *γ* = 90º
Volume	1244.82(14) Å^3^	1168.19(11) Å^3^	1260.06(11) Å^3^
*Z*	4	4	4
Density	1.202 mg/m^3^	1.269 mg/m^3^	1.309 mg/m^3^
Absorption coefficient	0.075 mm^−1^	0.080 mm^–1^	0.084 mm^−1^
Theta range	3.655–29.183º	3.187–29.557º	2.936–29.453º
Reflections collected	5094	3635	5405
Independent reflections	2839	2352	2909
Observed reflections	1999	2017	2171
Final *R* indices	*R*_1_ = 0.0505; *wR*2 = 0.1110	*R*_1_ = 0.0387; *wR*2 = 0.0841	*R*_1_ = 0.0450; *wR*2 = 0.1001
Goodness-of-fit on *F*^2^	1.042	1.056	1.022

## Data Availability

Not applicable.

## Supplementary Materials

The following are available online. ^1^H- and ^13^C-NMR spectra are available online. The CIF files of crystal structures have been deposited with the CCDC, and the crystal data and torsion angles for **5i**, **6e**, and **6l** are also provided.

## Sample Availability

Not available.
