# Supplementary material for: Regioselective Mercury(I)/Palladium(II)-Catalyzed Single-Step Approach for the Synthesis of Imines and 2-Substituted Indoles
Source: Molecules. 2021 Jul 5;26(13):4092. doi: 10.3390/molecules26134092 (PMC8271454; doi:10.3390/molecules26134092)
Supplement: Supplementary file 1 [file molecules-26-04092-s001.zip › molecules-1268032-supplementary.pdf]

SUPPLEMENTARY INFORMATION

Regioselective Mercury(I)/Palladium(II)-catalyzed Single-Step Approach for the Synthesis of Imines and 2-Substituted Indoles

Rsuini U. Gutiérrez[1], Mayra Hernández-Montes[1], Aarón Mendieta-Moctezuma[2], Francisco Delgado[1] and Joaquín Tamariz[1,]*

[1] Departamento de Química Orgánica, Escuela Nacional de Ciencias Biológicas, Instituto Politécnico Nacional, Prolongación de Carpio y Plan de Ayala S/N, 11340 Mexico City, Mexico

[2] Centro de Investigación en Biotecnología Aplicada, Instituto Politécnico Nacional, Carretera Estatal Santa Inés Tecuexcomac-Tepetitla, Km 1.5, 90700 Tlaxcala, Mexico

* Author to whom correspondence should be addressed; E-Mail: jtamarizm@gmail.com; jtamarizm@ipn.mx

1. Appendix 1. ^{1}H and ^{13}C NMR spectra of all compounds. S2-S30

2. Appendix 2. X-Ray crystallographic structures of **5i**, **6e** and **6l** S31-S36

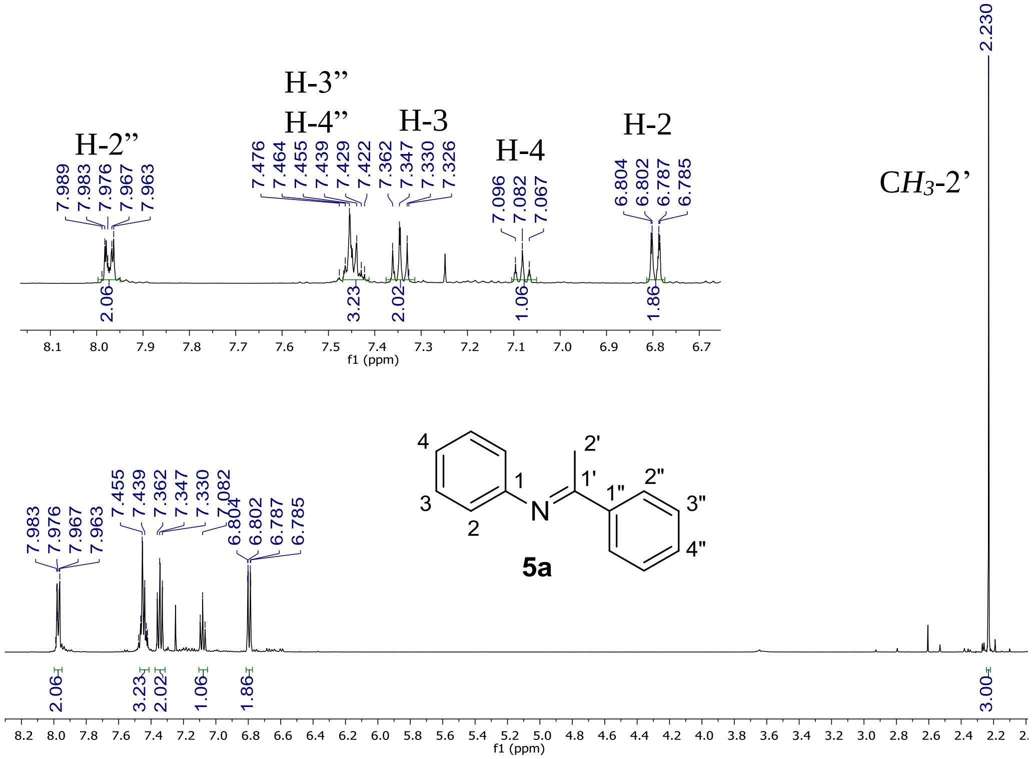

Figure S1. Spectrum of ^{1}H NMR (500 MHz, $CDCl_3$) of **5a**.

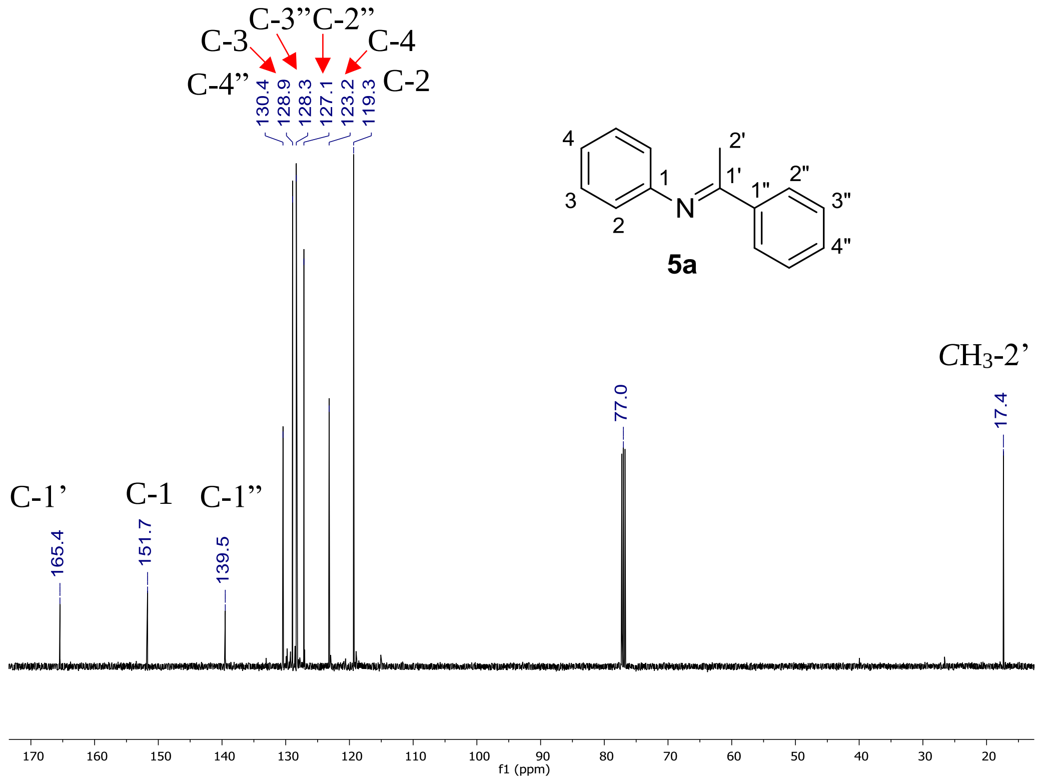

Figure S2. Spectrum of ^{13}C NMR (125 MHz, $CDCl_3$) of **5a**.

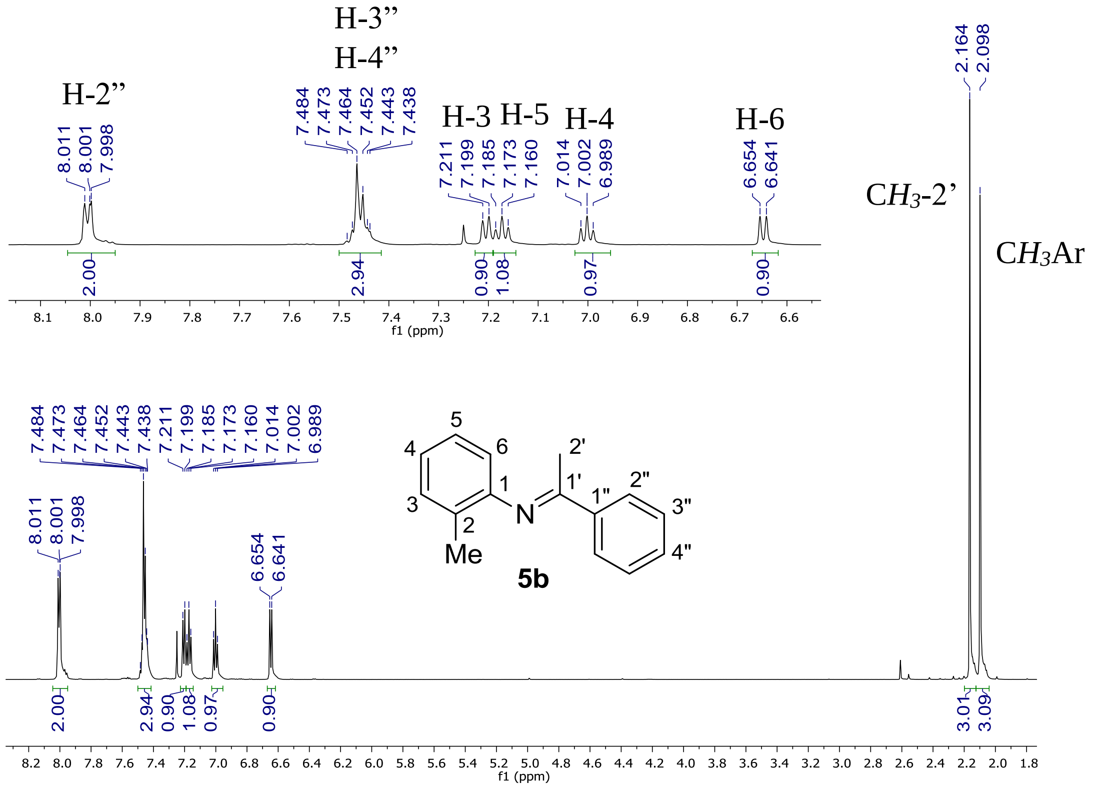

Figure S3. Spectrum of ^{1}H NMR (600 MHz, $CDCl_3$) of **5b**.

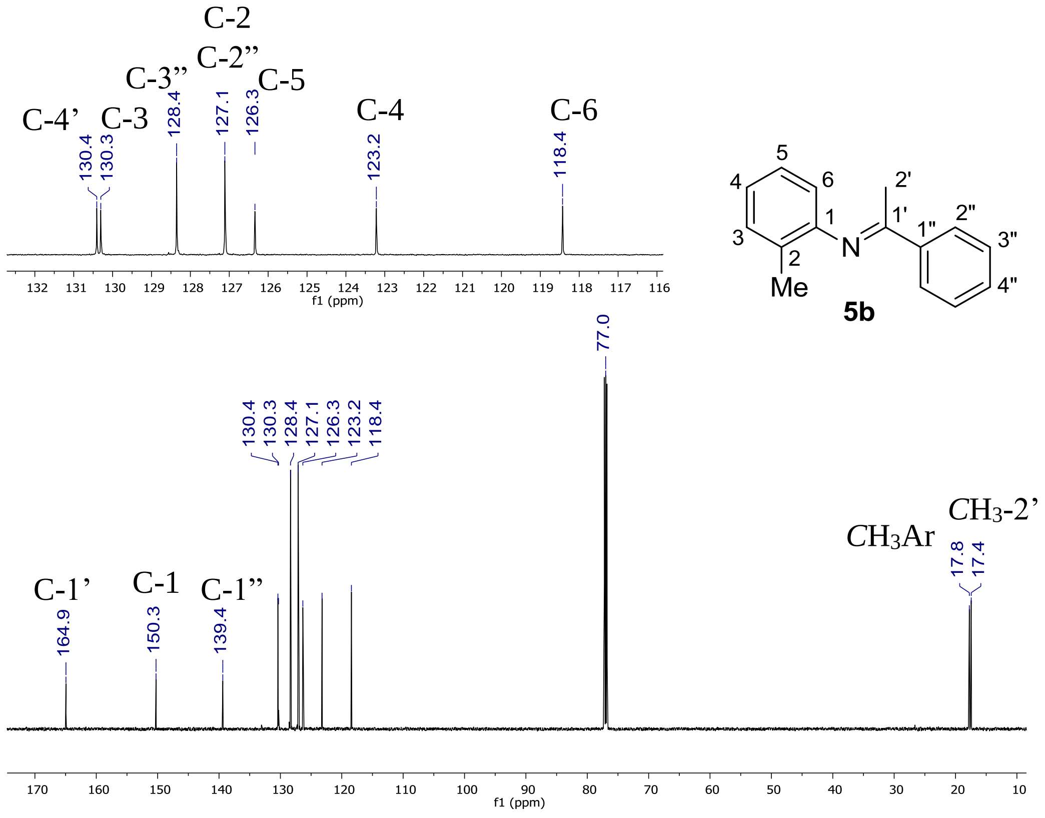

Figure S4. Spectrum of ^{13}C NMR (150 MHz, $CDCl_3$) of **5b**.

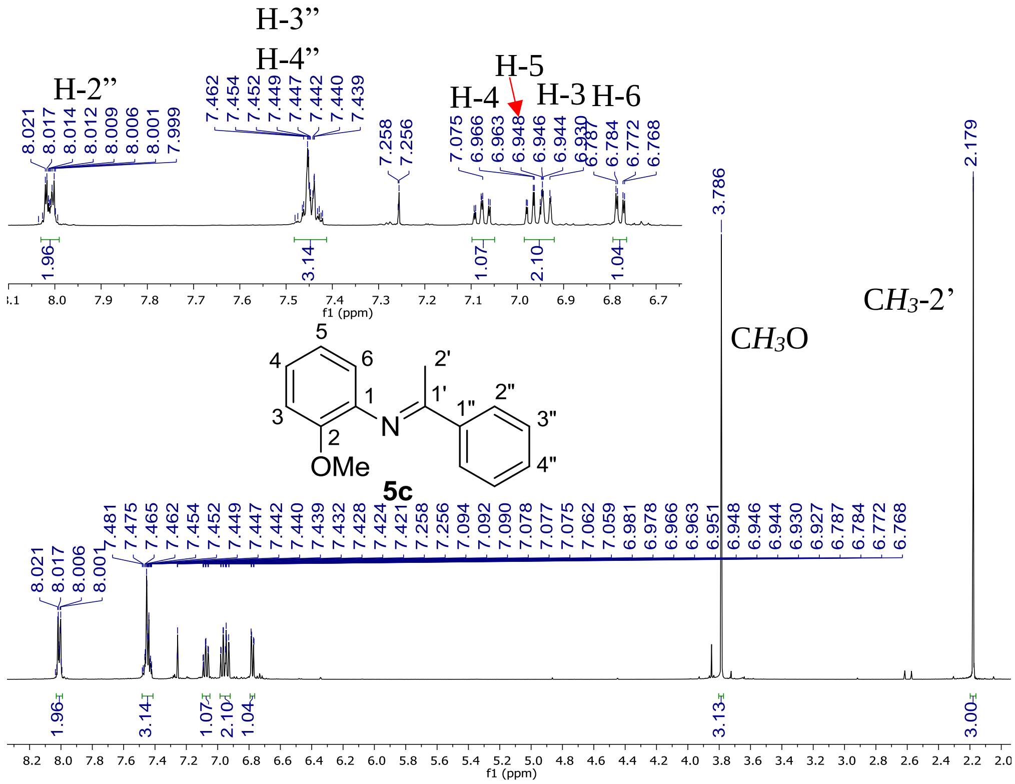

Figure S5. Spectrum of ^{1}H NMR (500 MHz, $CDCl_3$) of **5c**.

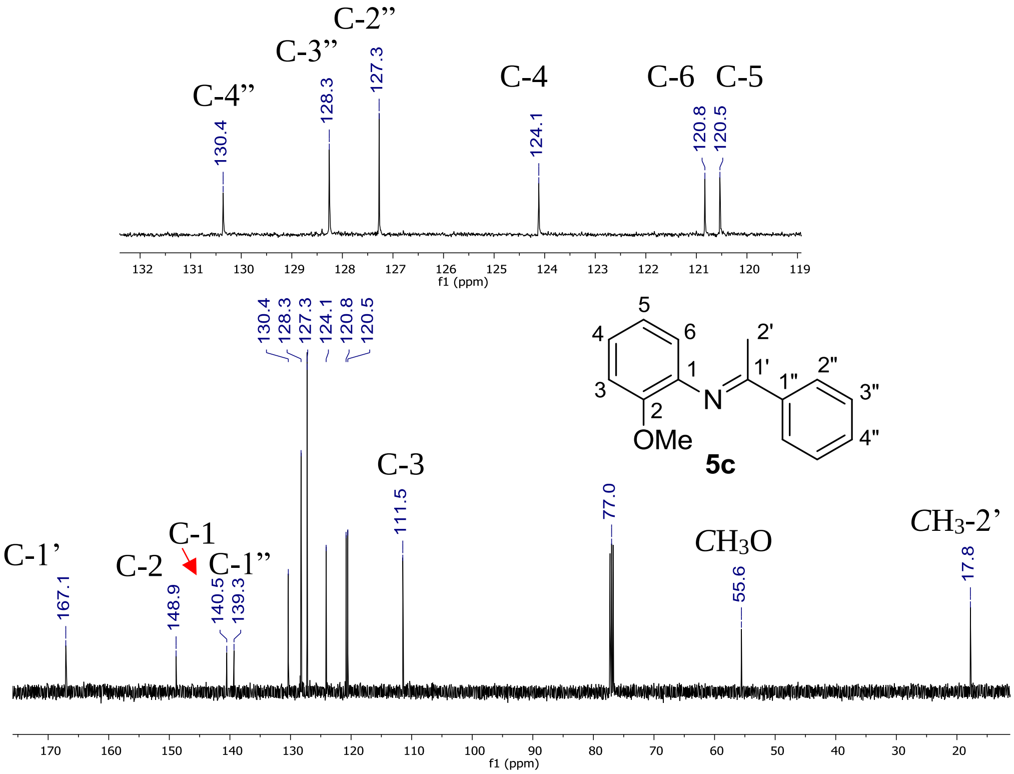

Figure S6. Spectrum of ^{13}C NMR (125 MHz, $CDCl_3$) of **5c**.

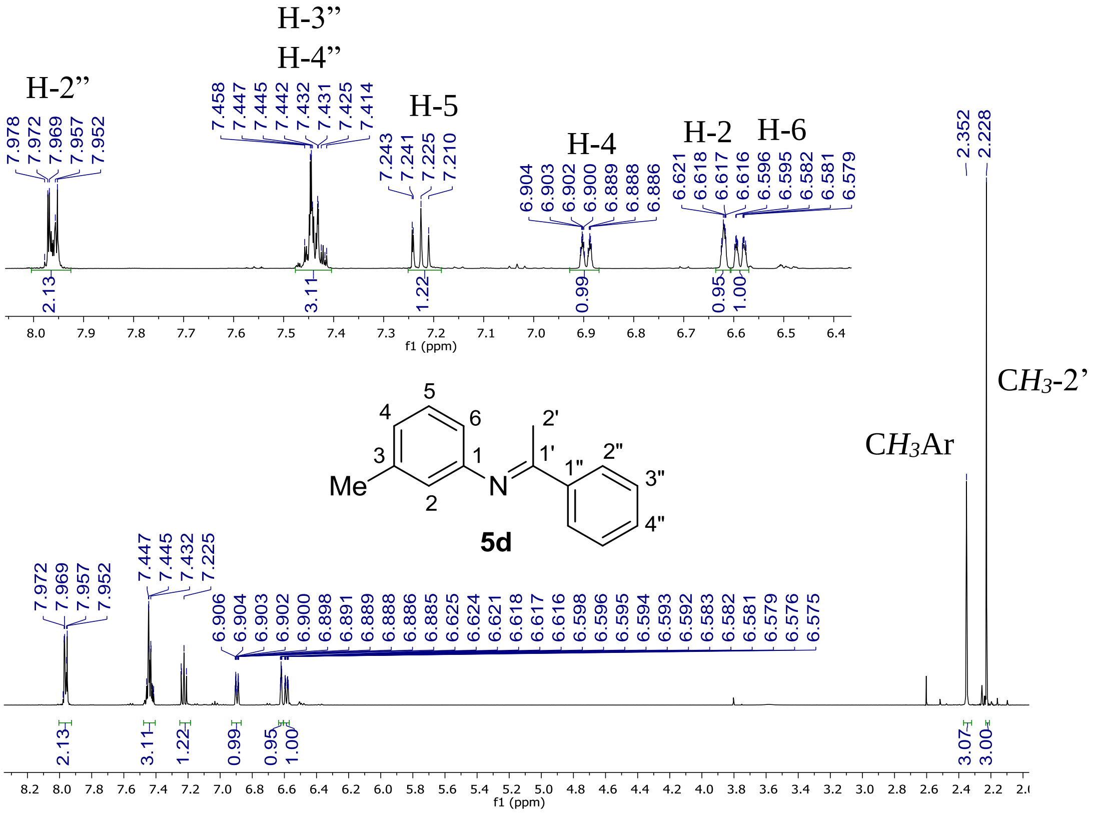

Figure S7. Spectrum of ^{1}H NMR (500 MHz, $CDCl_3$) of **5d**.

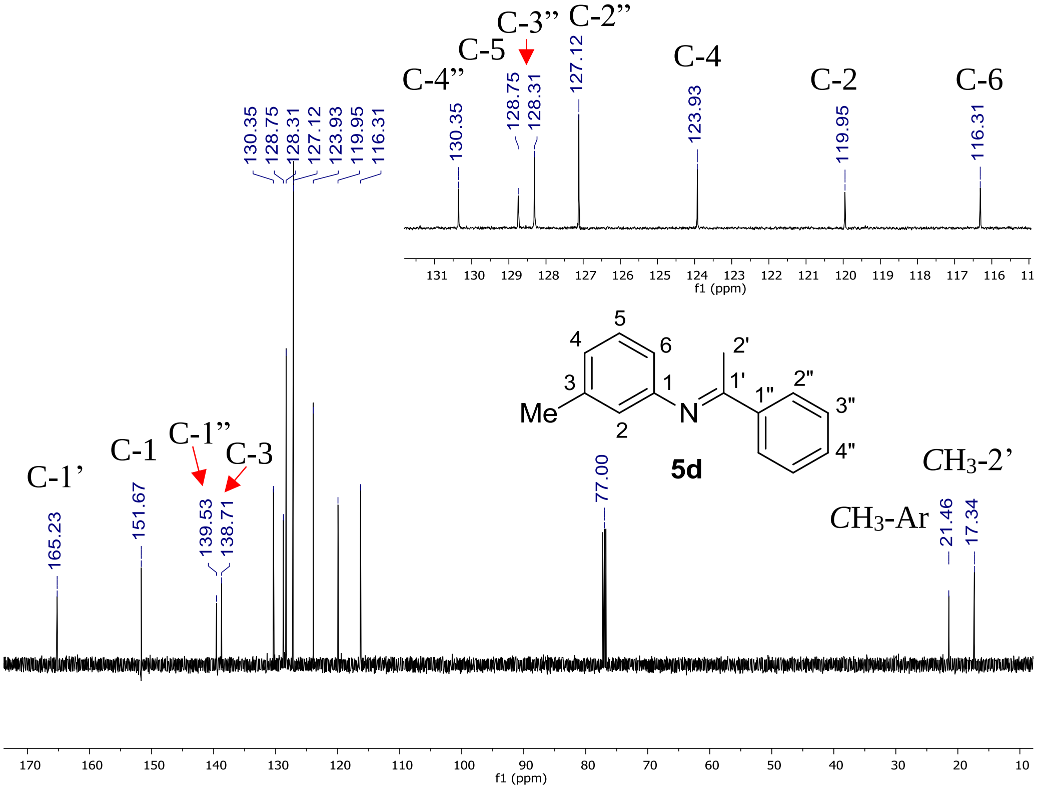

Figure S8. Spectrum of ^{13}C (125 MHz, $CDCl_3$) of **5d**.

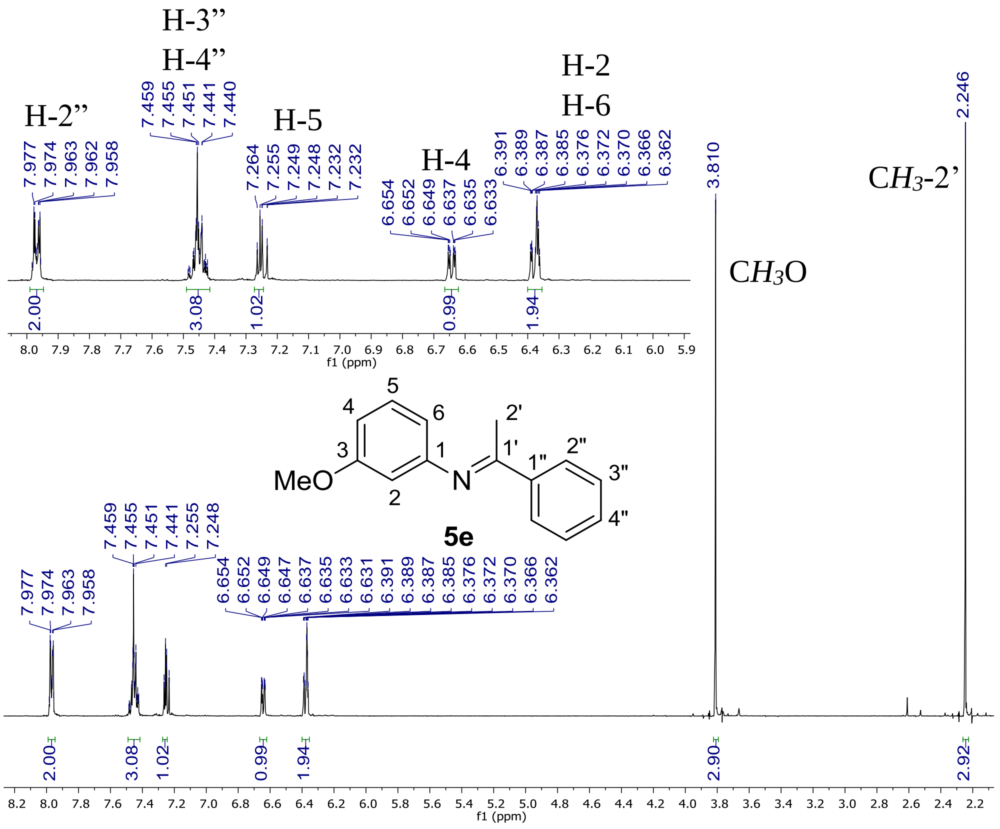

Figure S9. Spectrum of ^{1}H NMR (500 MHz, $CDCl_3$) of **5e**.

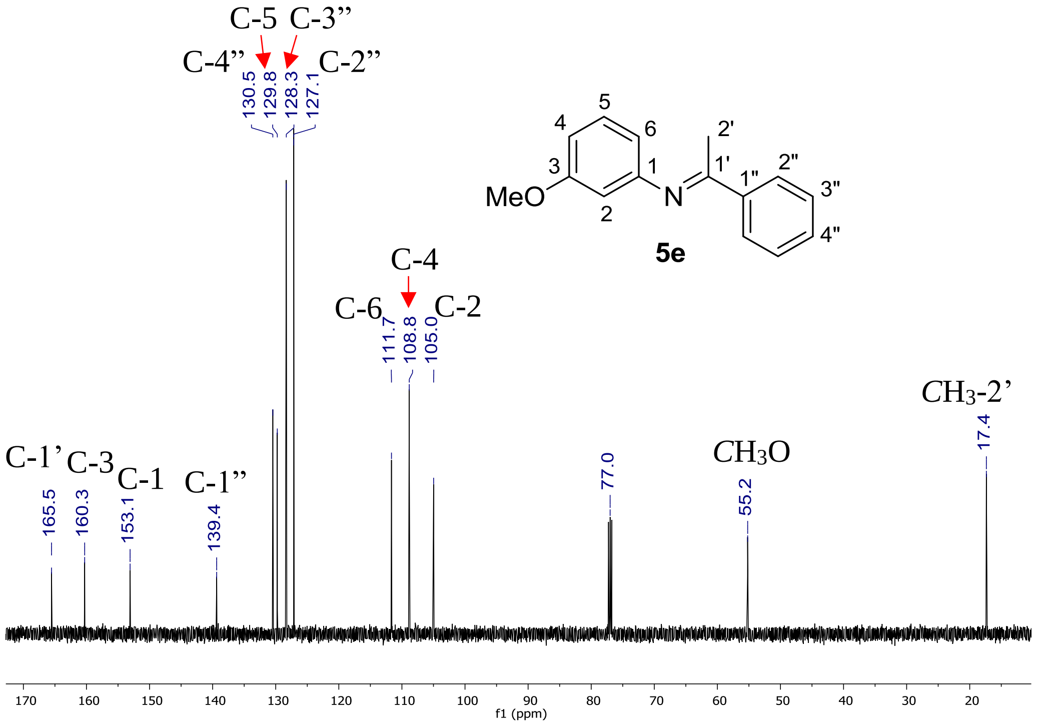

Figure S10. Spectrum of ^{13}C NMR (125 MHz, $CDCl_3$) of **5e**.

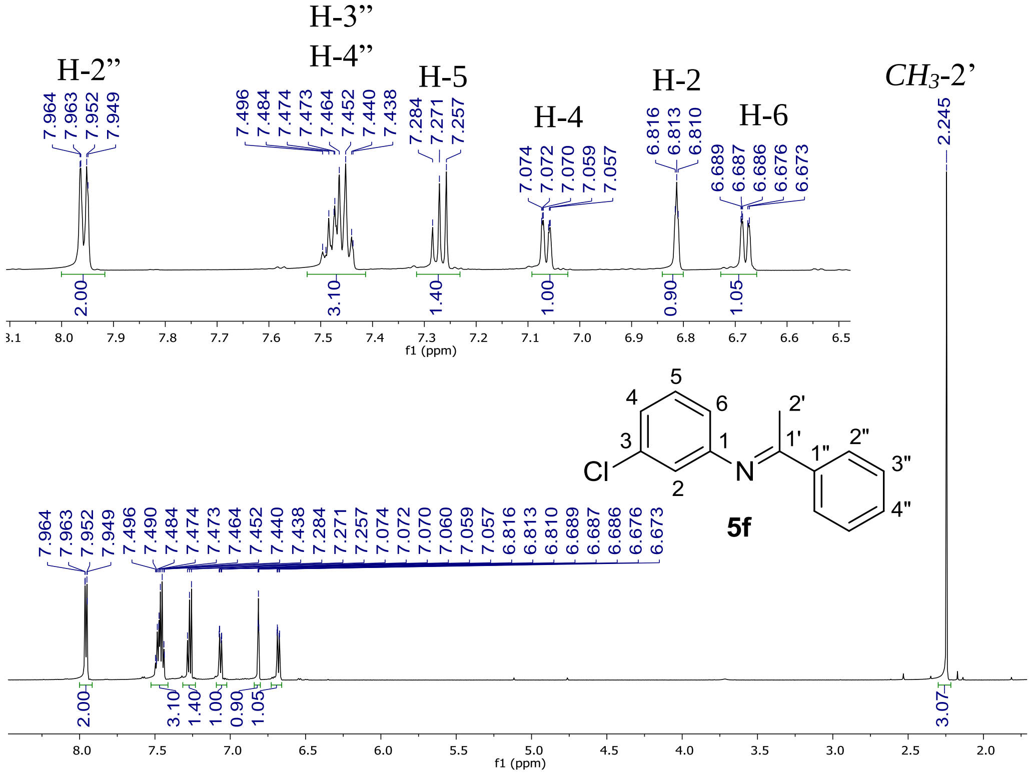

Figure S11. Spectrum of ^{1}H NMR (600 MHz, $CDCl_3$) of **5f**.

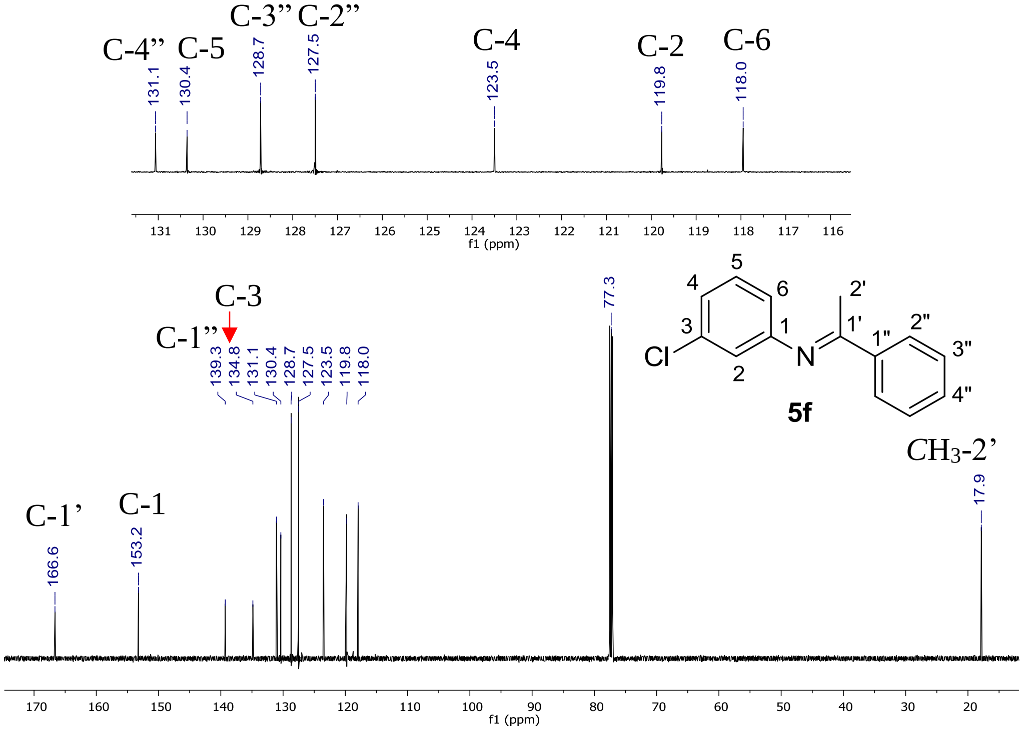

Figure S12. Spectrum of ^{13}C NMR (150 MHz, $CDCl_3$) of **5f**.

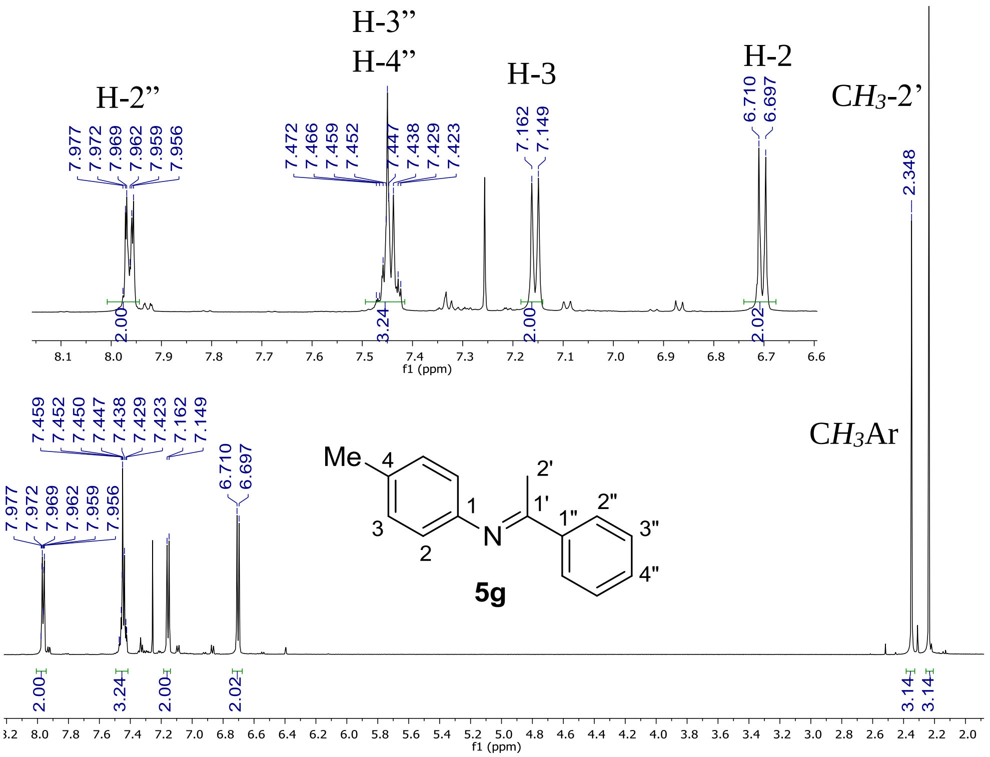

Figure S13. Spectrum of ^{1}H NMR (600 MHz, $CDCl_3$) of **5g**.

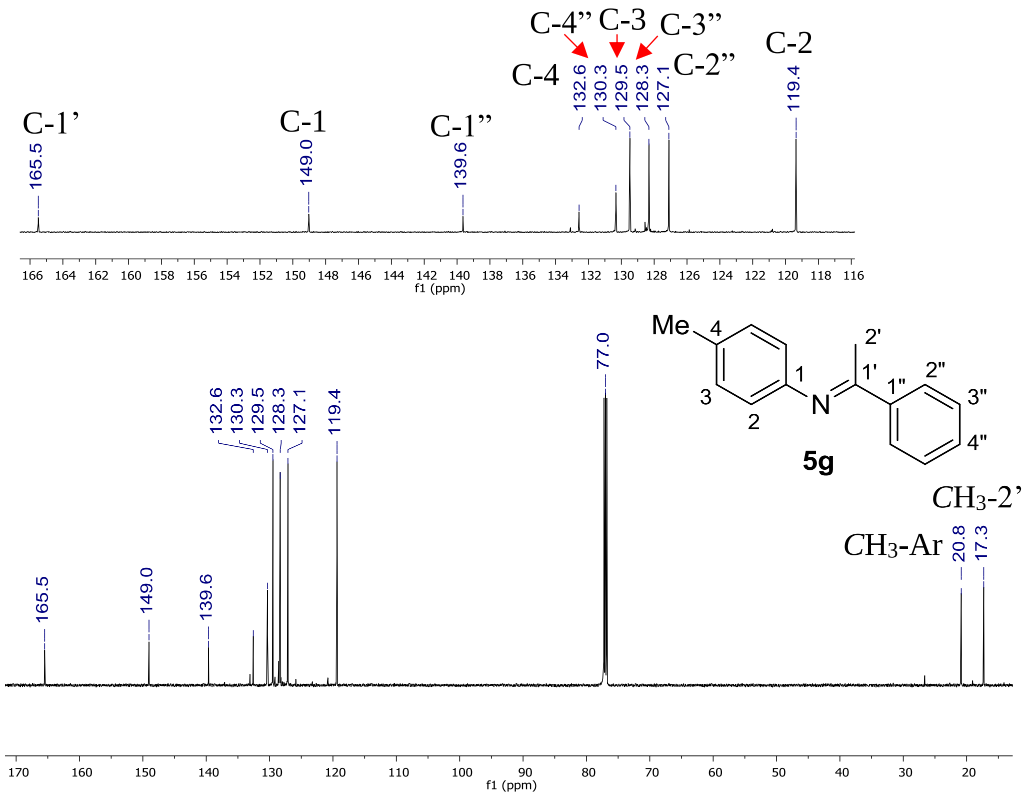

Figure S14. Spectrum of ^{13}C NMR (150 MHz, $CDCl_3$) of **5g**.

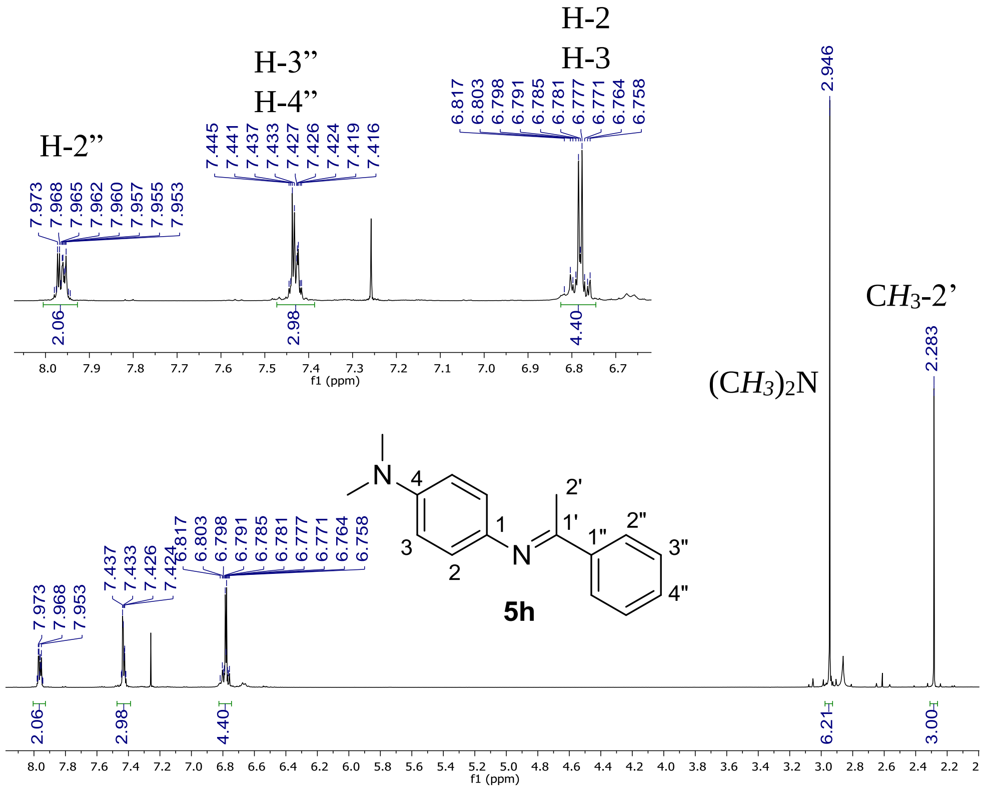

Figure S15. Spectrum of ^{1}H NMR (500 MHz, $CDCl_3$) of **5h**.

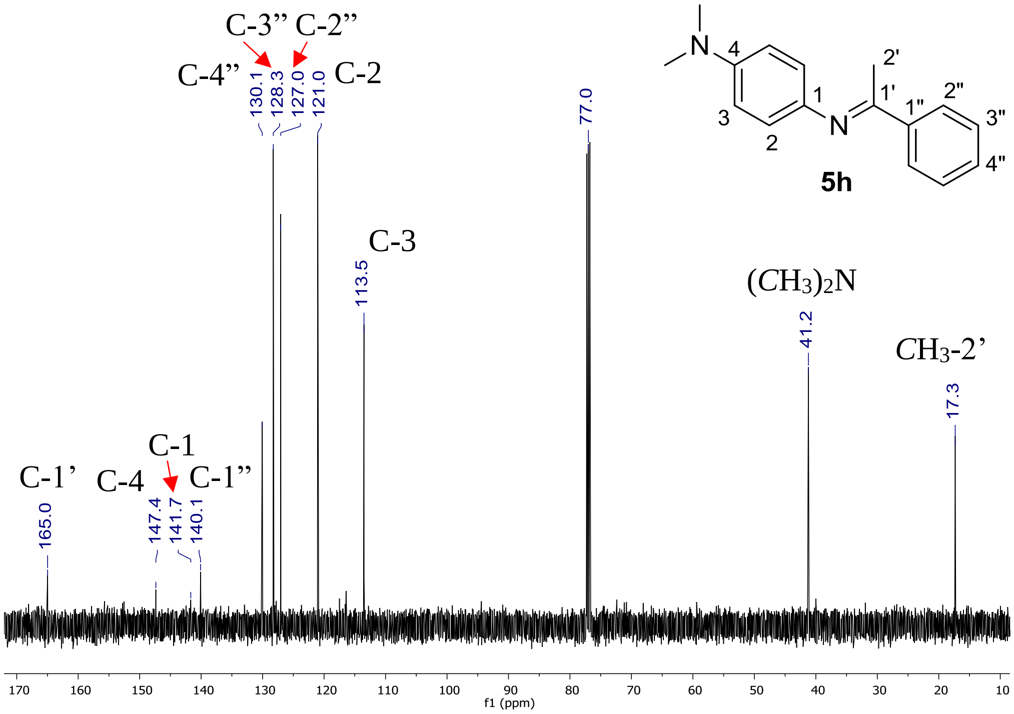

Figure S16. Spectrum of ^{13}C (125 MHz, $CDCl_3$) of **5h**.

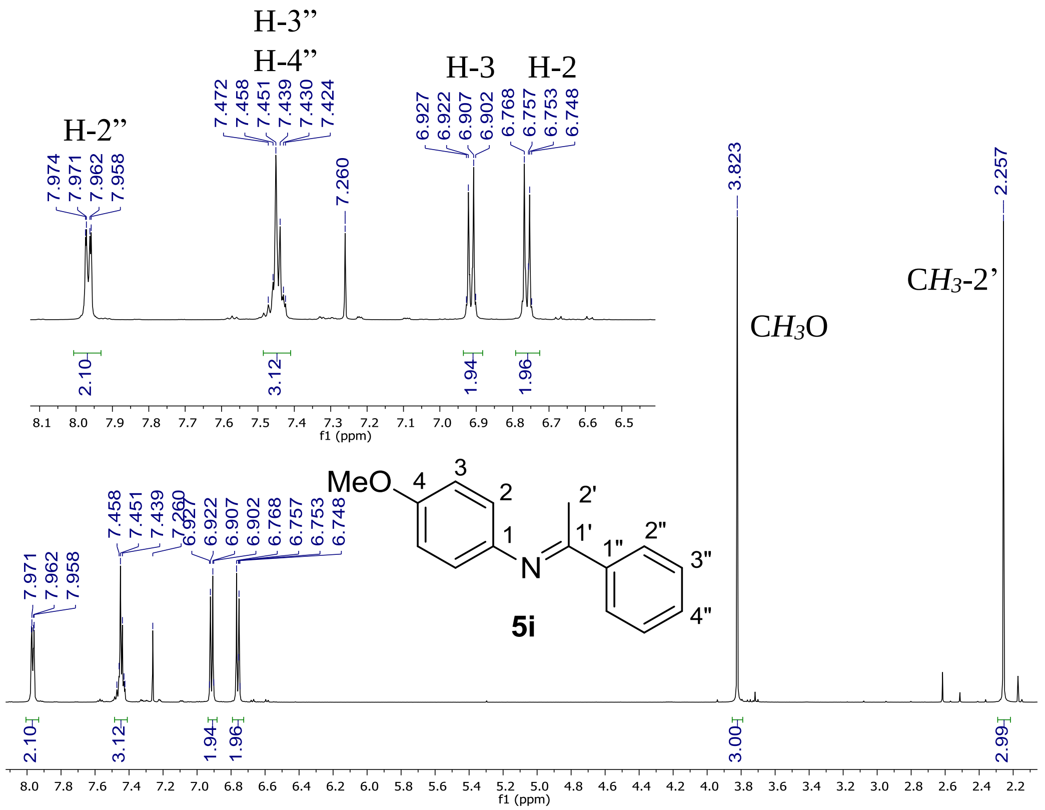

Figure S17. Spectrum of ^{1}H NMR (600 MHz, $CDCl_3$) of **5i**.

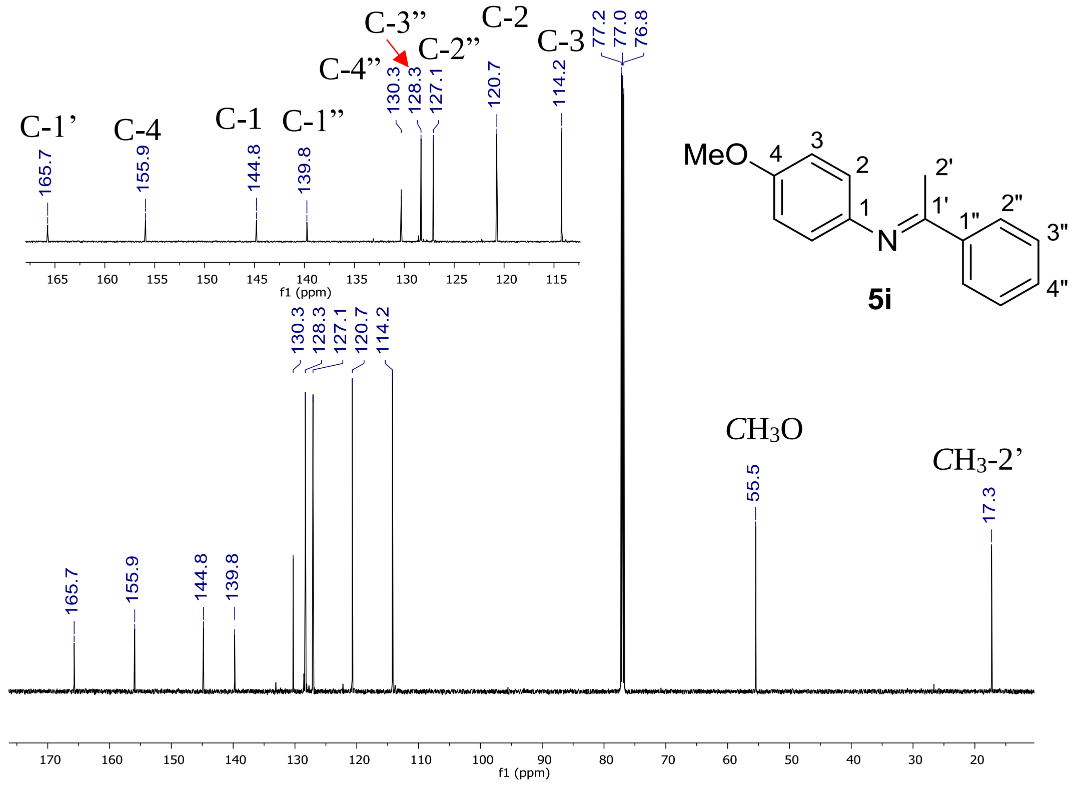

Figure S18. Spectrum of ^{13}C NMR (150 MHz, $CDCl_3$) of **5i**.

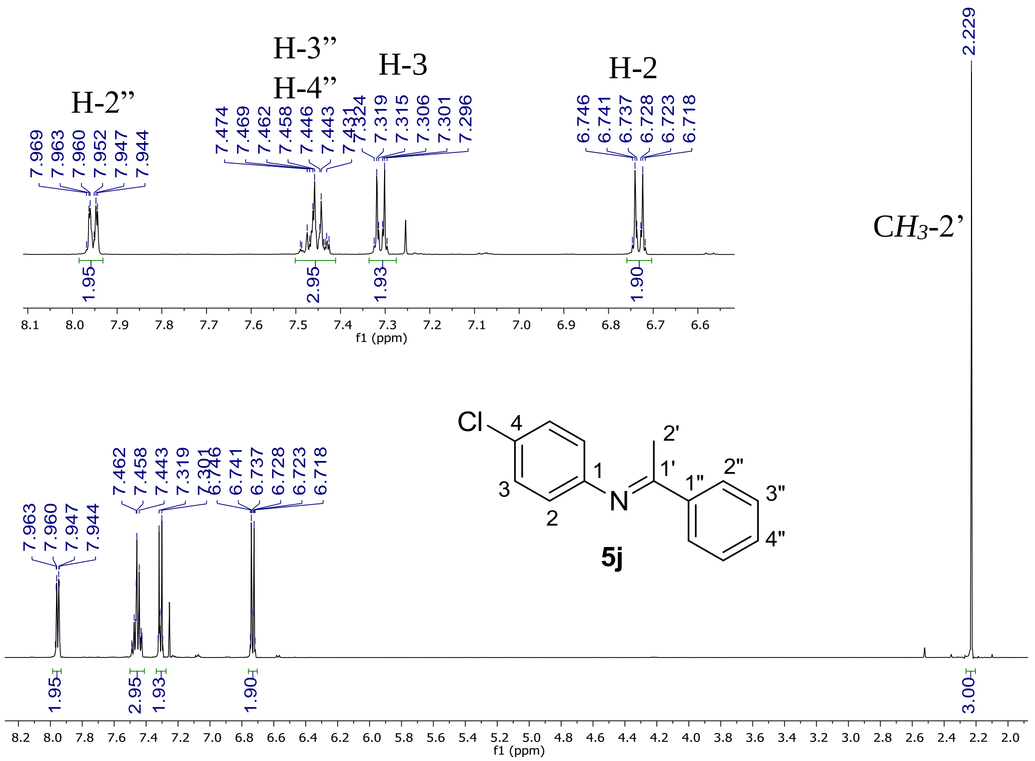

Figure S19. Spectrum of ^{1}H NMR (500 MHz, $CDCl_3$) of **5j**.

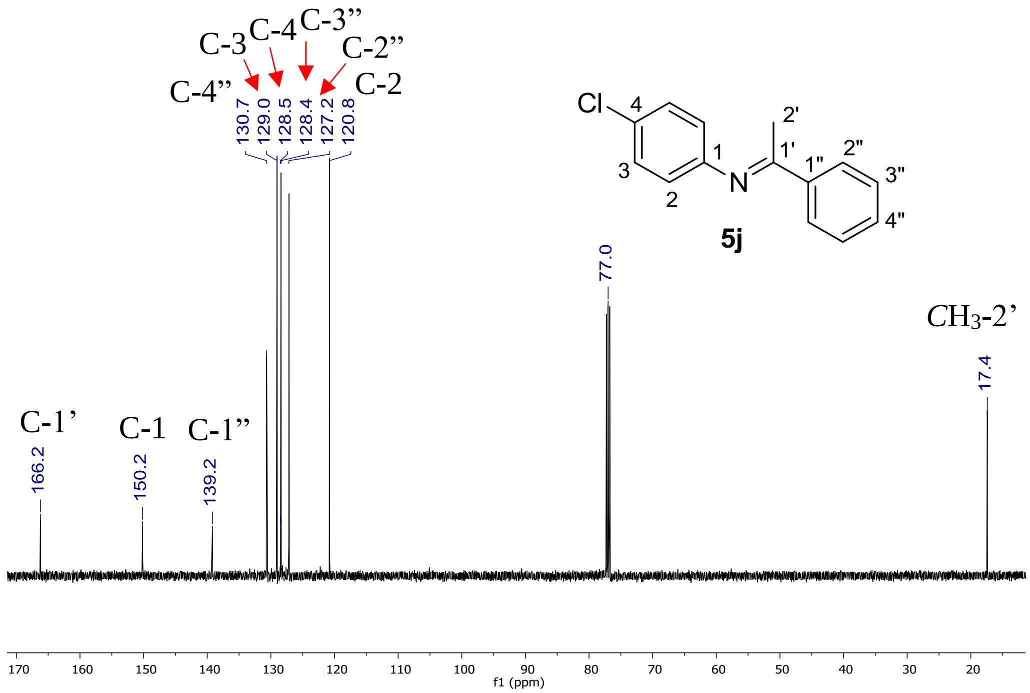

Figure S20. Spectrum of ^{13}C NMR (125 MHz, $CDCl_3$) of **5j**.

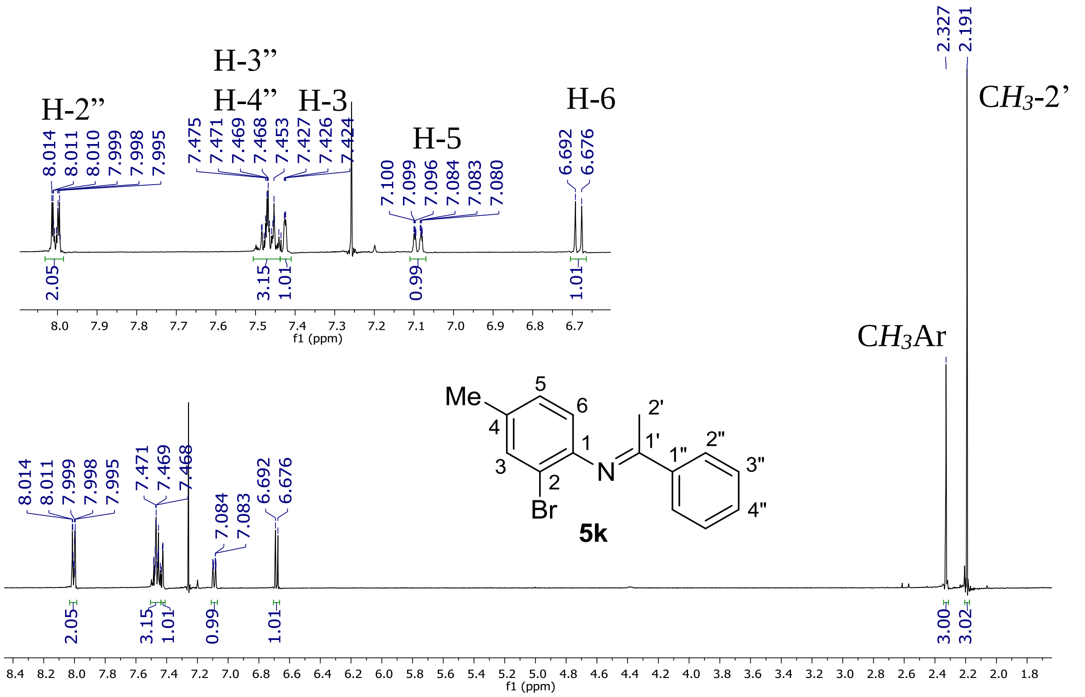

Figure S21. Spectrum of ^{1}H NMR (500 MHz, $CDCl_3$) of **5k**.

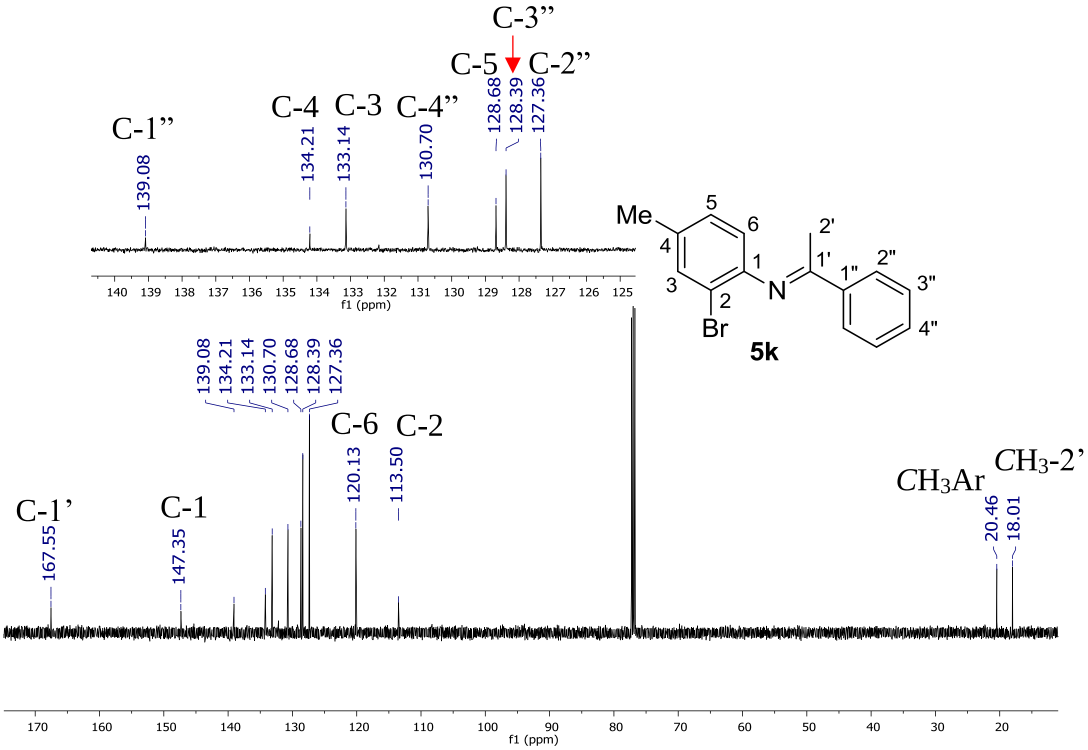

Figure S22. Spectrum of ^{13}C NMR (125 MHz, $CDCl_3$) of **5k**.

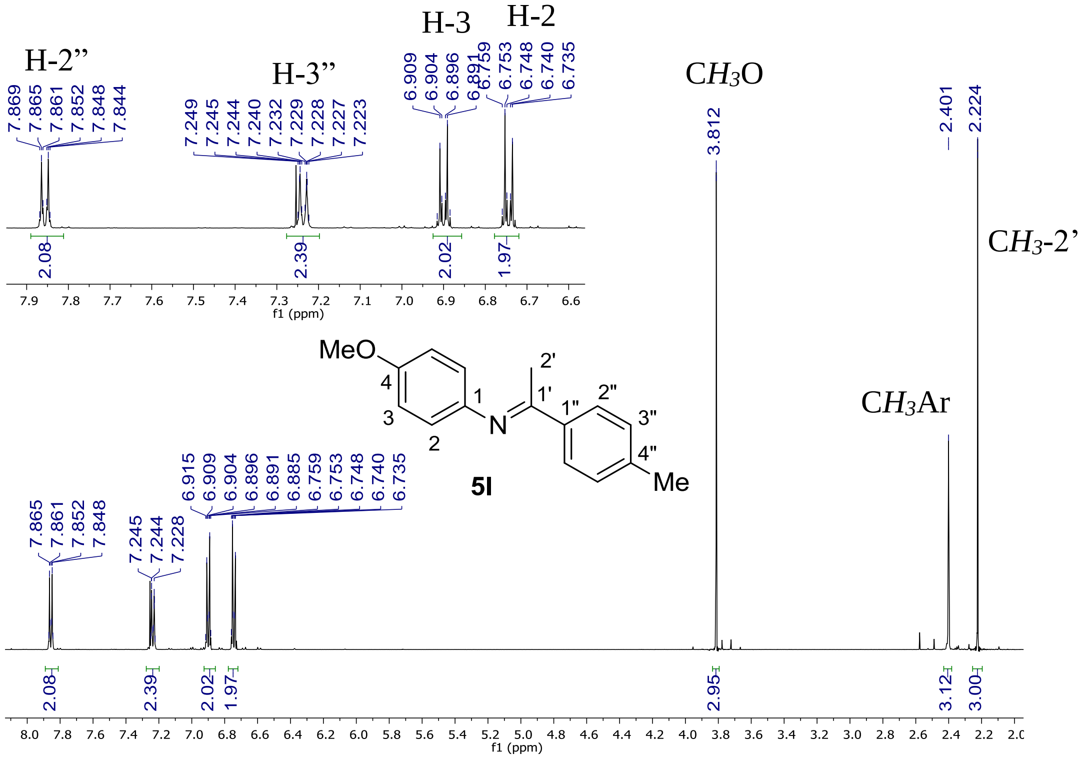

Figure S23. Spectrum of ^{1}H NMR (500 MHz, $CDCl_3$) of **5l**.

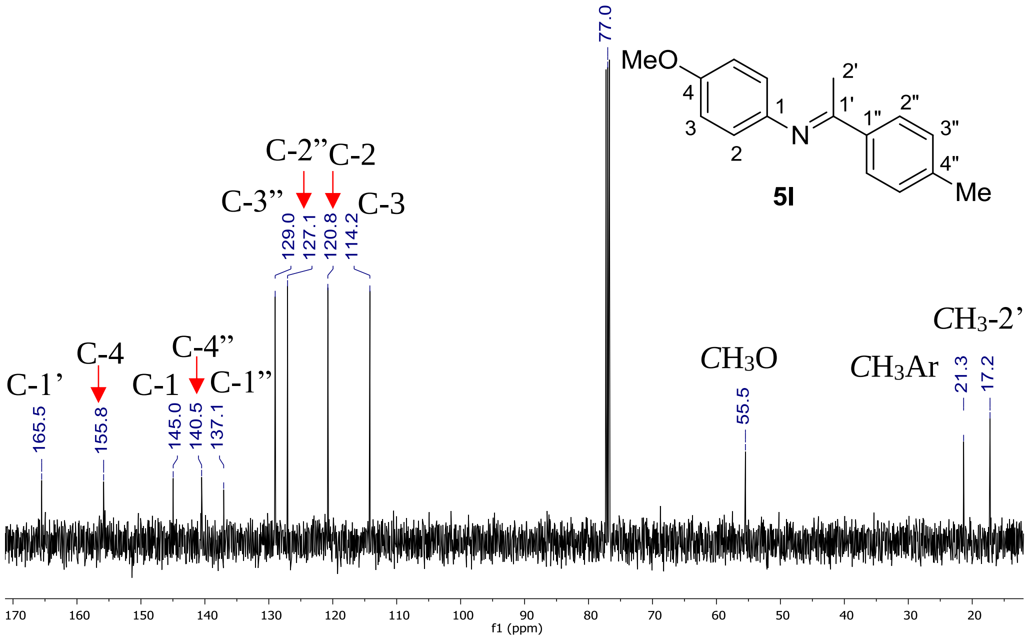

Figure S24. Spectrum of ^{13}C NMR (125 MHz, $CDCl_3$) of **5l**.

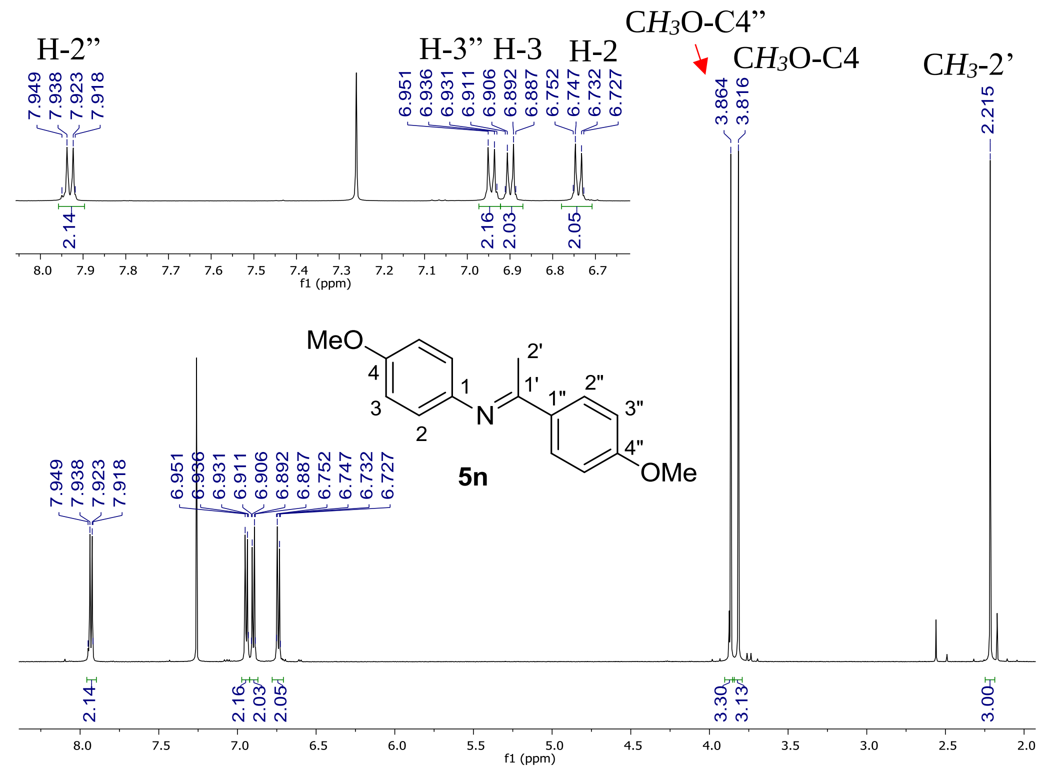

Figure S25. Spectrum of ^{1}H NMR (600 MHz, $CDCl_3$) of **5n**.

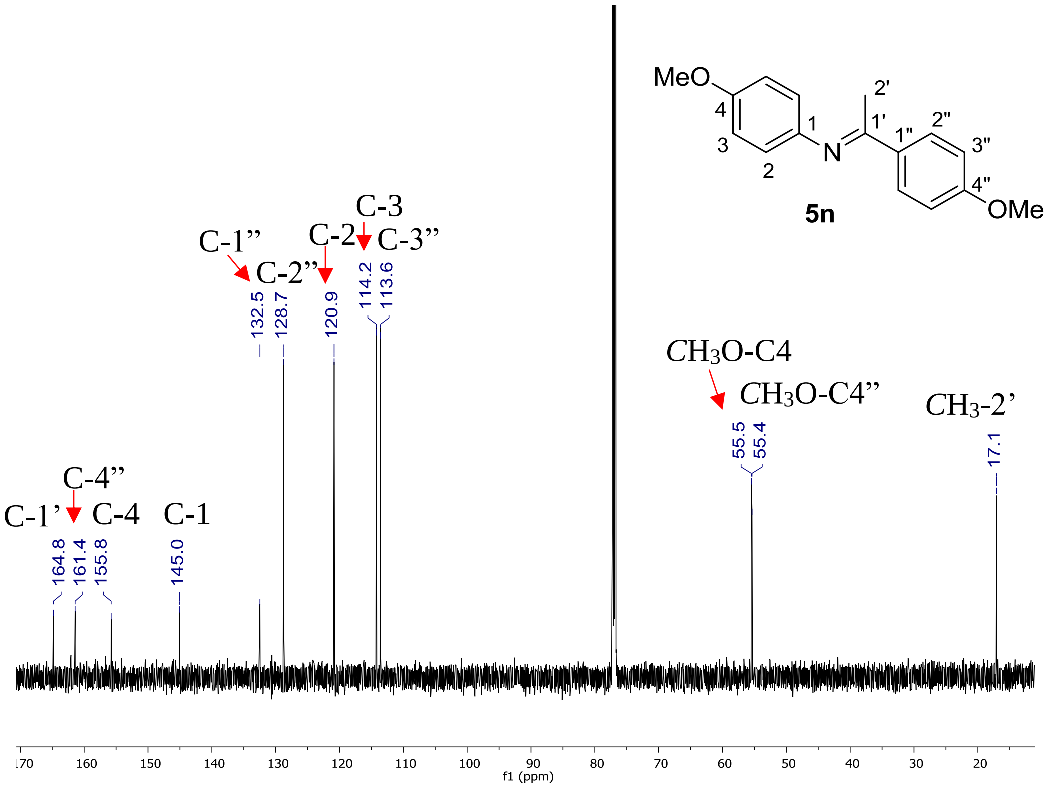

Figure S26. Spectrum of ^{13}C NMR (150 MHz, $CDCl_3$) of **5n**.

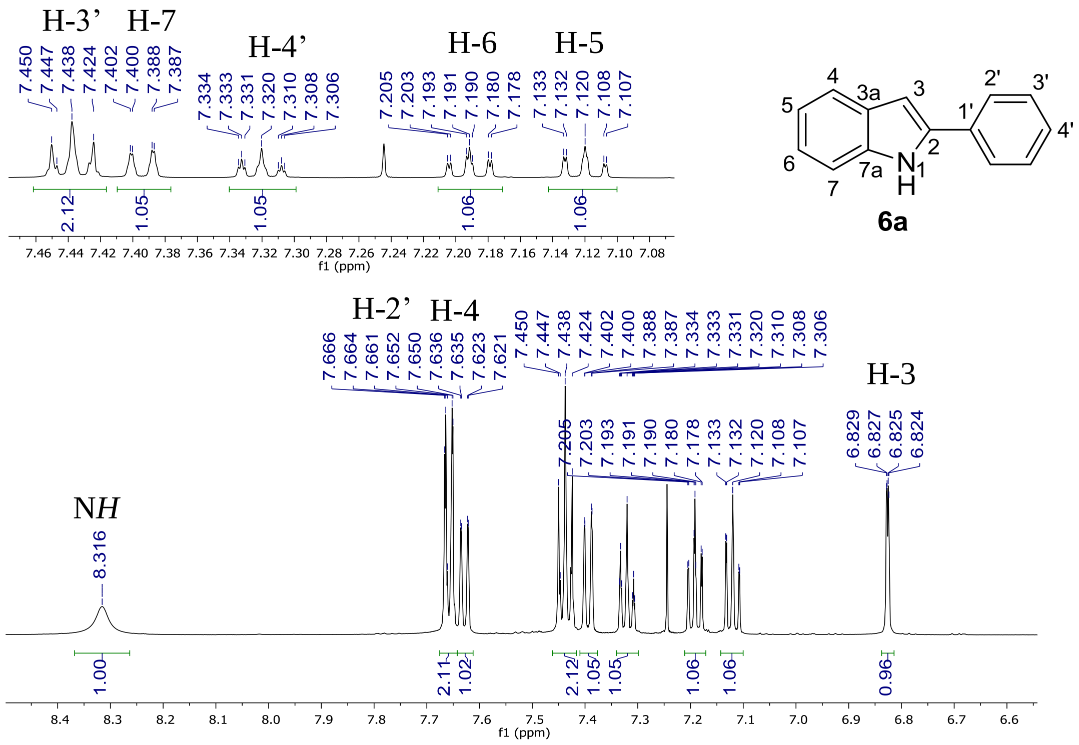

Figure S27. Spectrum of ^{1}H NMR (600 MHz, $CDCl_3$) of **6a**.

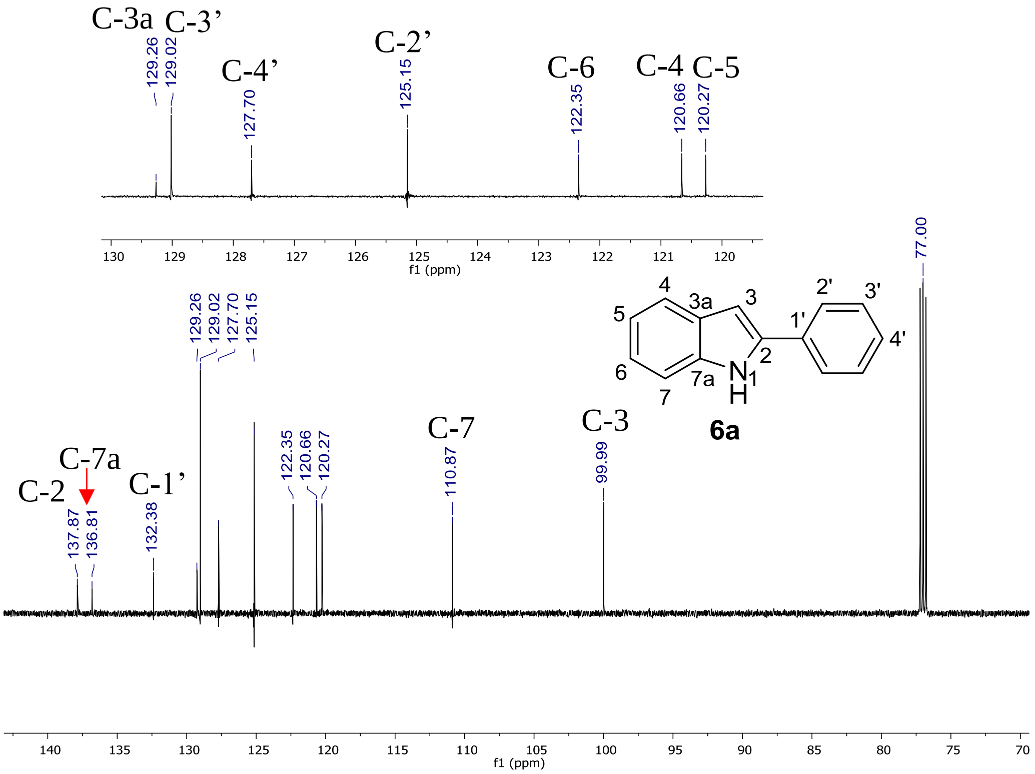

Figure S28. Spectrum of ^{13}C NMR (150 MHz, $CDCl_3$) of **6a**.

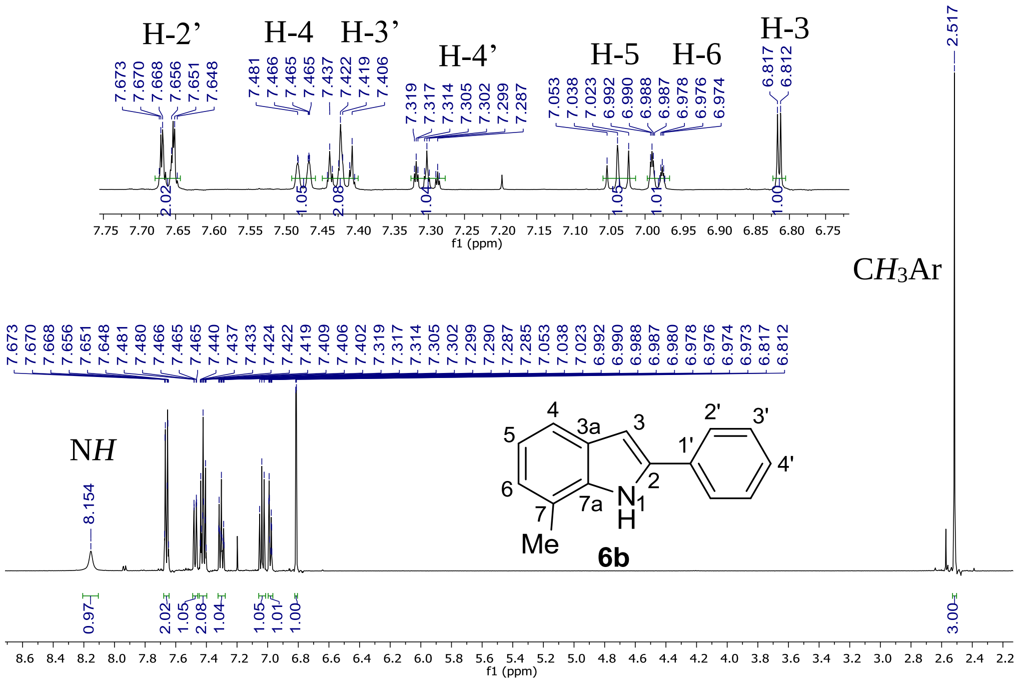

Figure S29. Spectrum of ^{1}H NMR (500 MHz, $CDCl_3$) of **6b**.

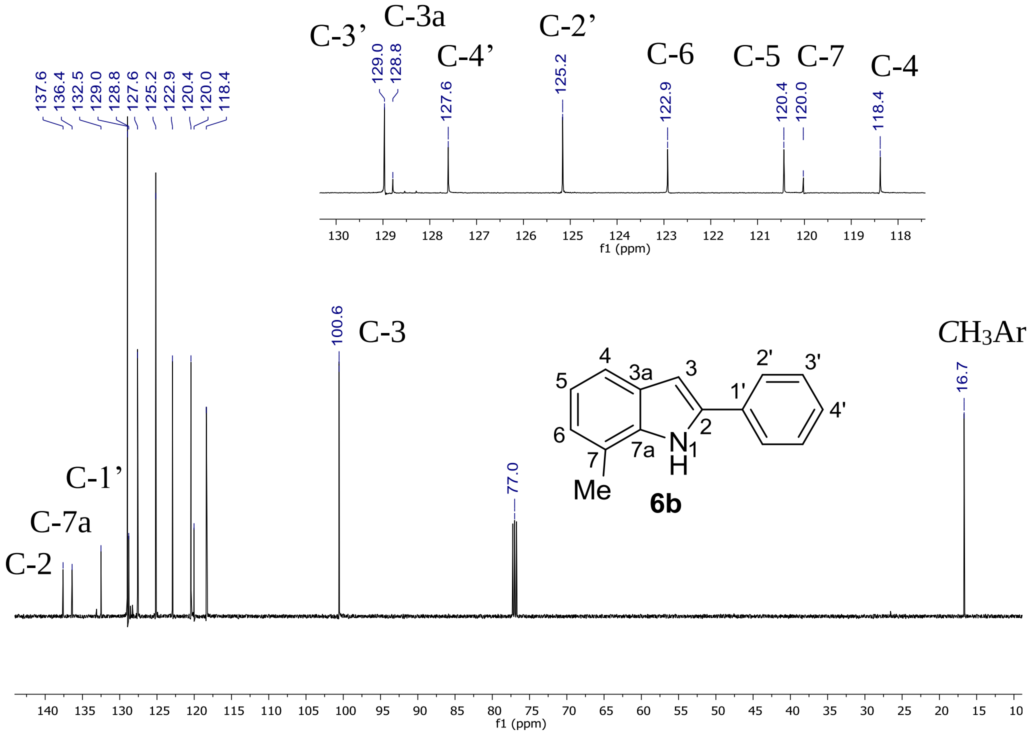

Figure S30. Spectrum of ^{13}C NMR (125 MHz, $CDCl_3$) of **6b**.

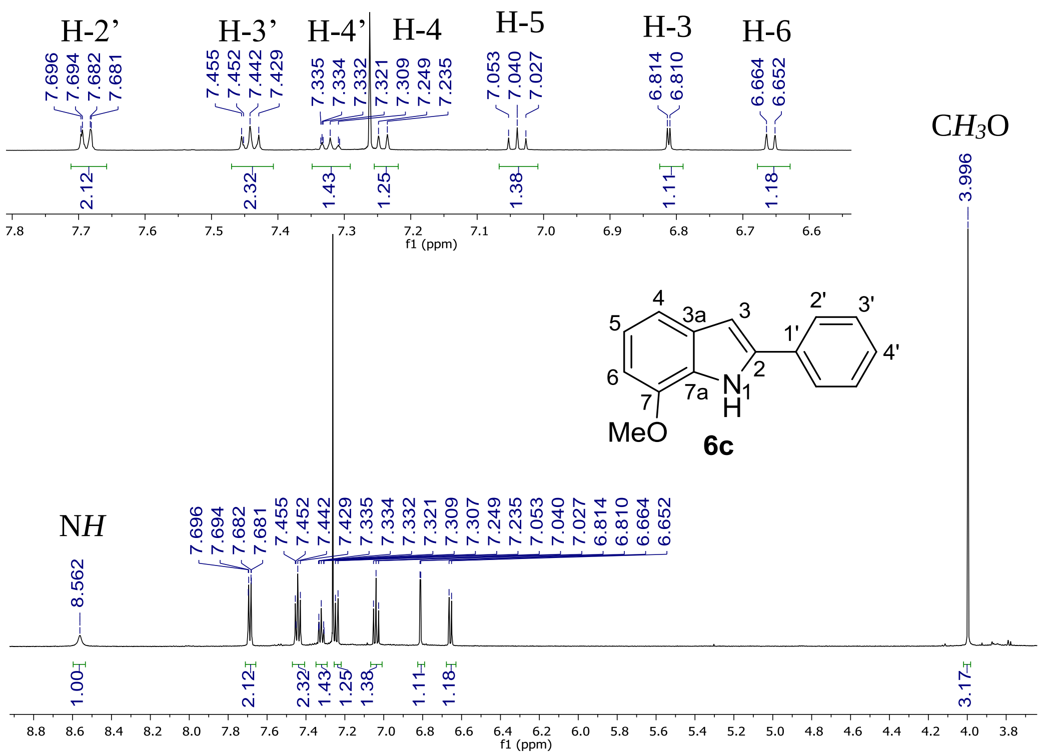

Figure S31. Spectrum of ^{1}H NMR (600 MHz, $CDCl_3$) of **6c**.

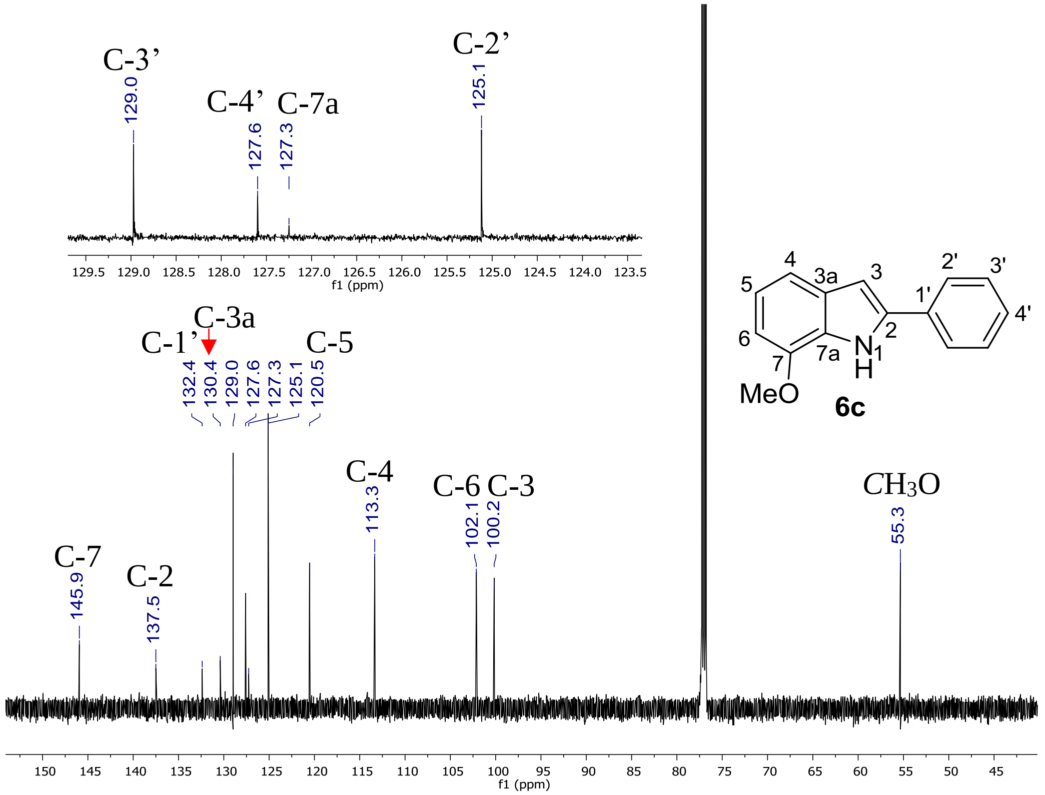

Figure S32. Spectrum of ^{13}C NMR (150 MHz, $CDCl_3$) of **6c**.

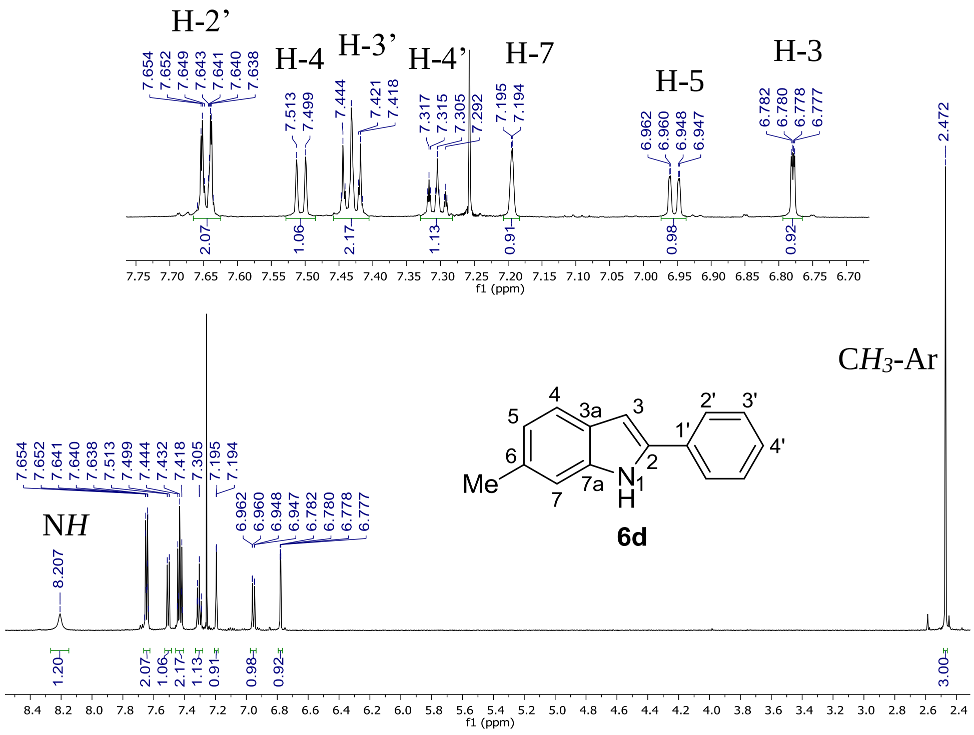

Figure S33. Spectrum of ^{1}H NMR (600 MHz, $CDCl_3$) of **6d**.

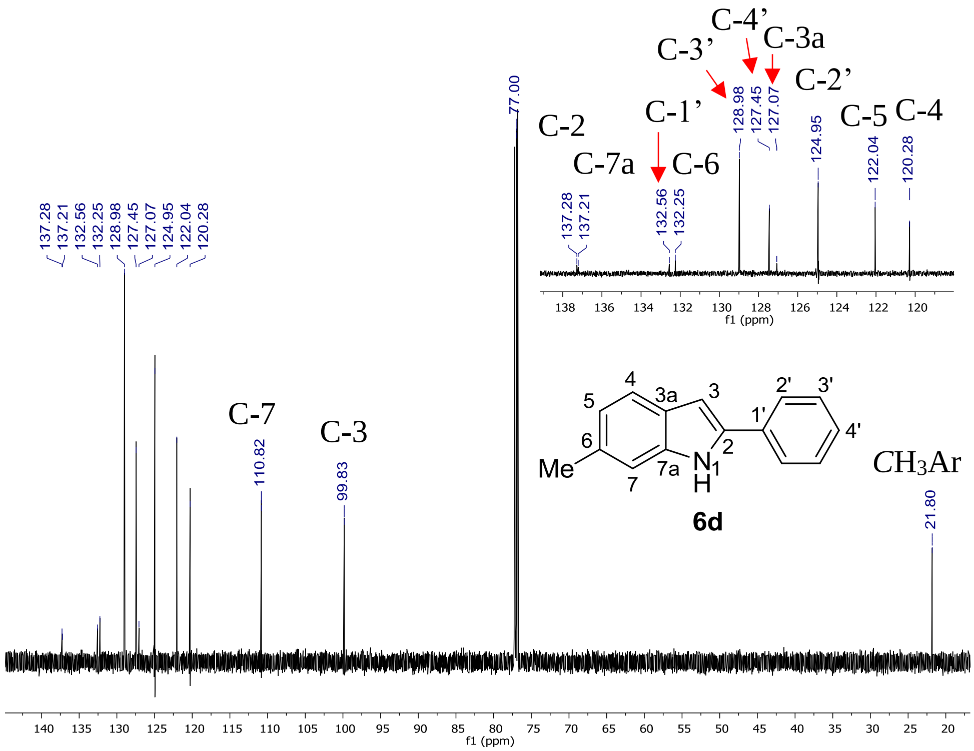

Figure S34. Spectrum of ^{13}C NMR (150 MHz, $CDCl_3$) of **6d**.

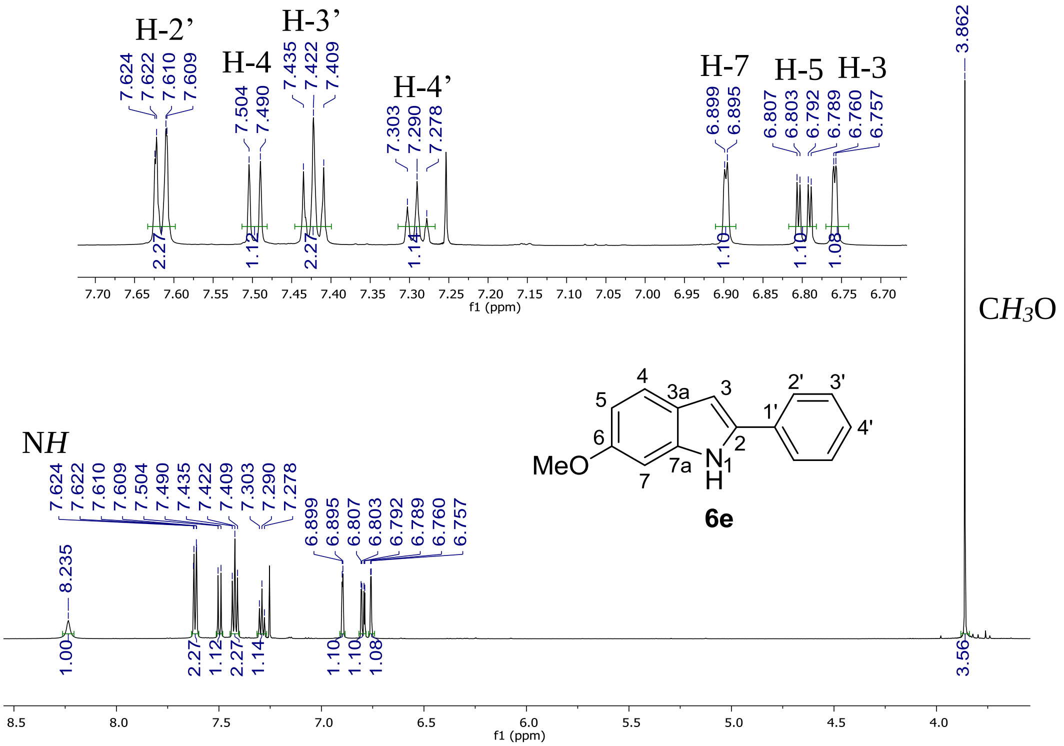

Figure S35. Spectrum of ^{1}H NMR (600 MHz, $CDCl_3$) of **6e**.

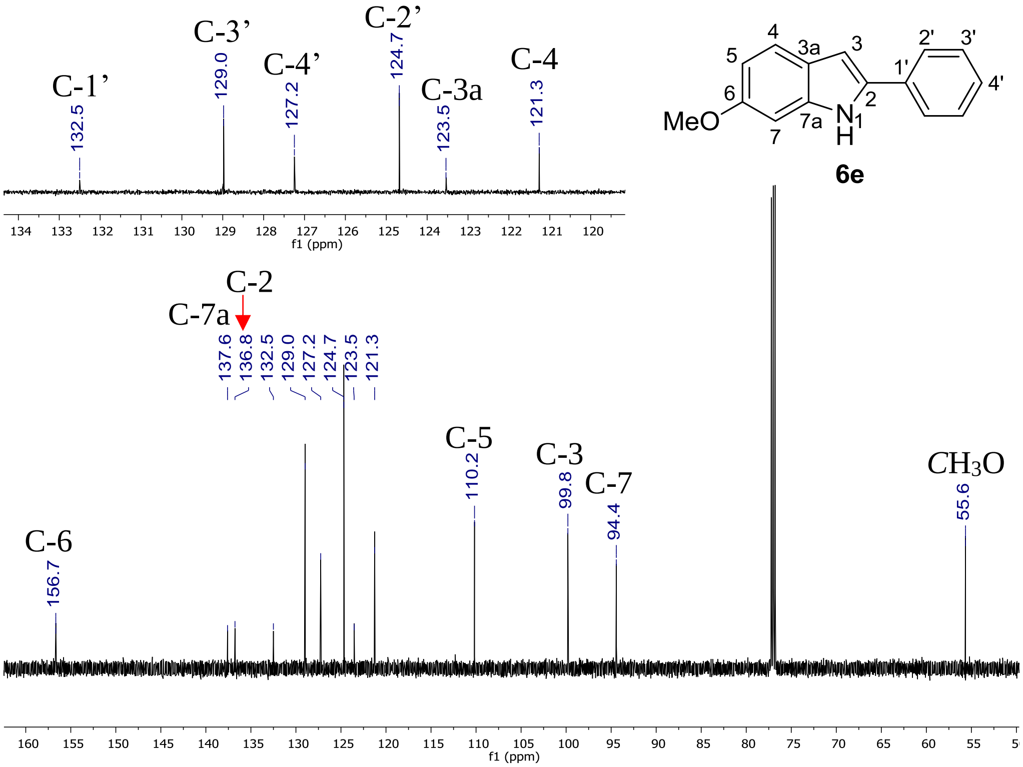

Figure S36. Spectrum of ^{13}C NMR (150 MHz, $CDCl_3$) de **6e**.

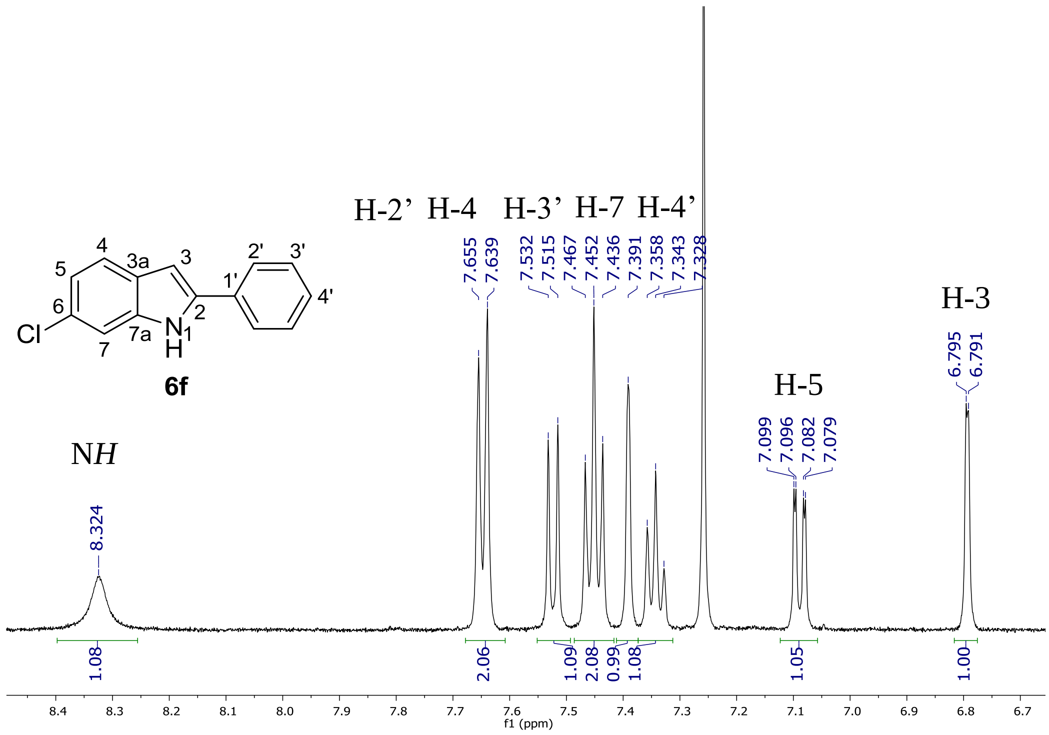

Figure S37. Spectrum of ^{1}H (500 MHz, $CDCl_3$) of **6f**.

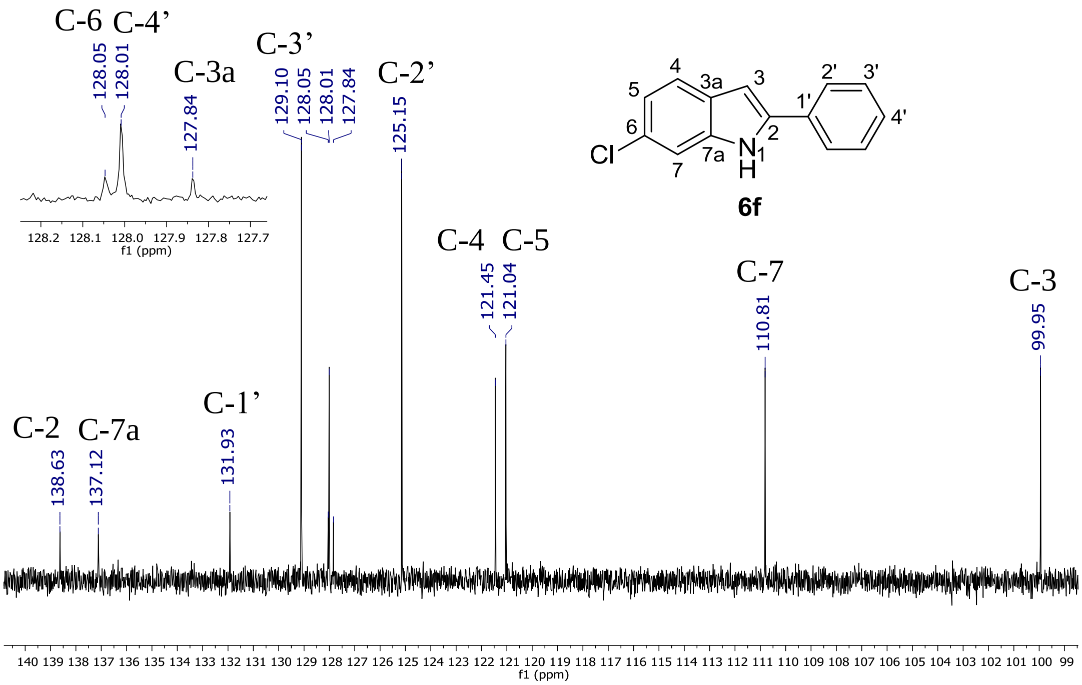

Figure S38. Spectrum of ^{13}C NMR (125 MHz, $CDCl_3$) of **6f**.

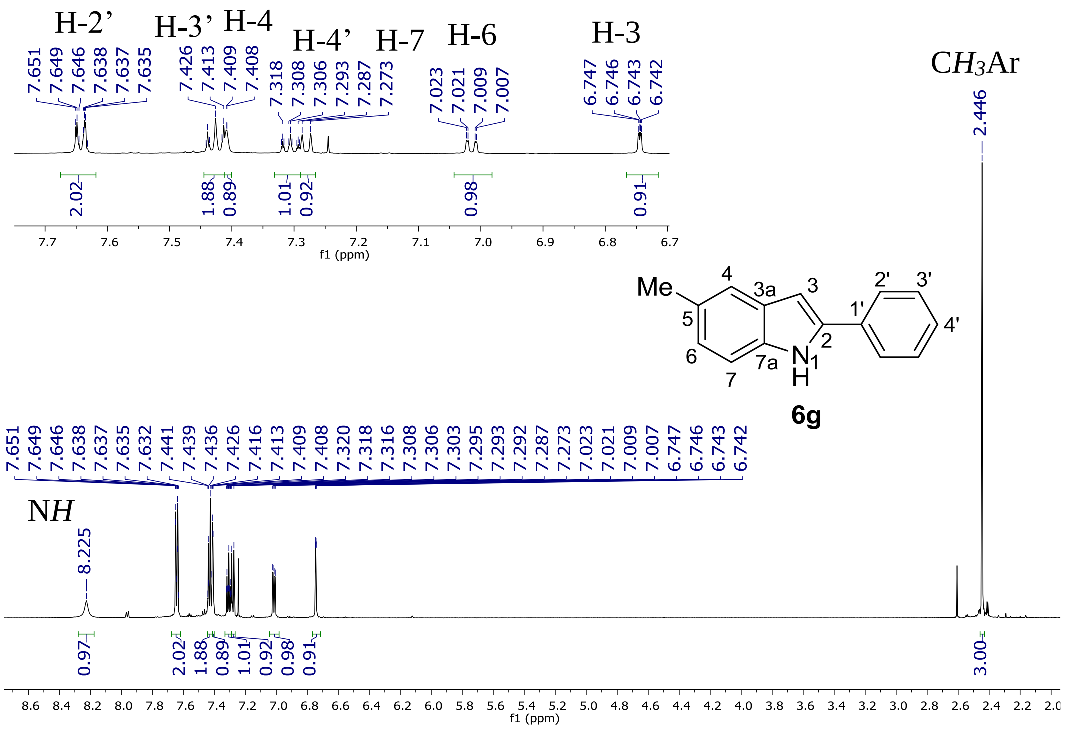

Figure S39. Spectrum of ^{1}H NMR (600 MHz, $CDCl_3$) of **6g**.

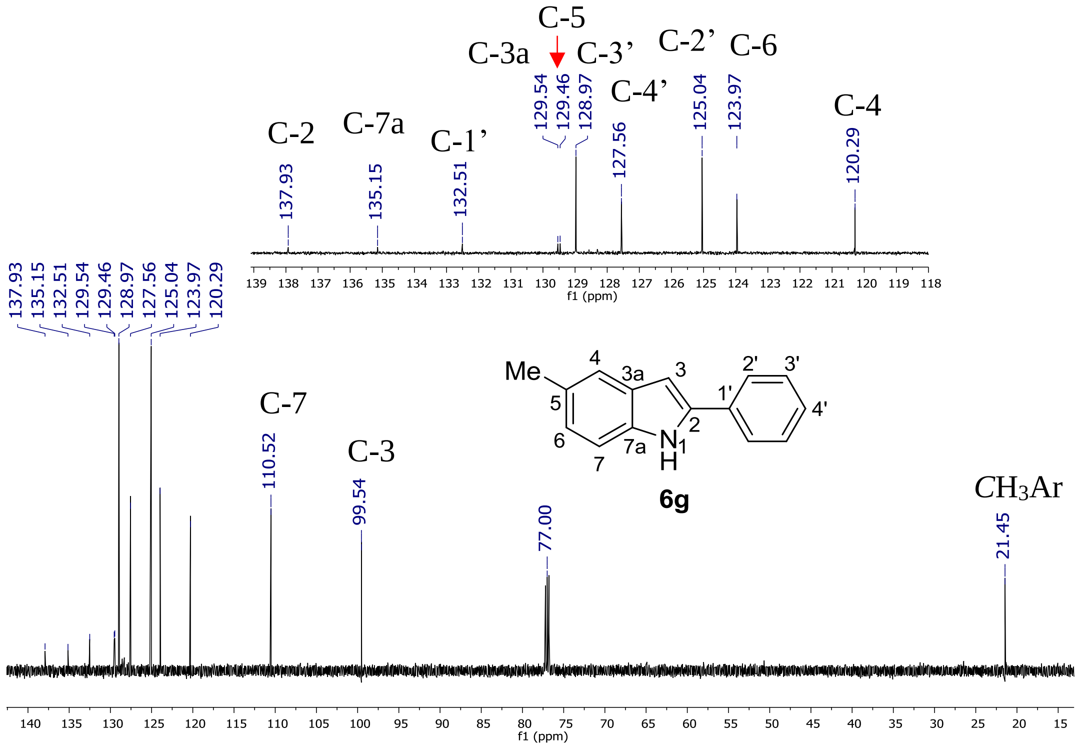

Figure S40. Spectrum of ^{13}C NMR (150 MHz, $CDCl_3$) of **6g**.

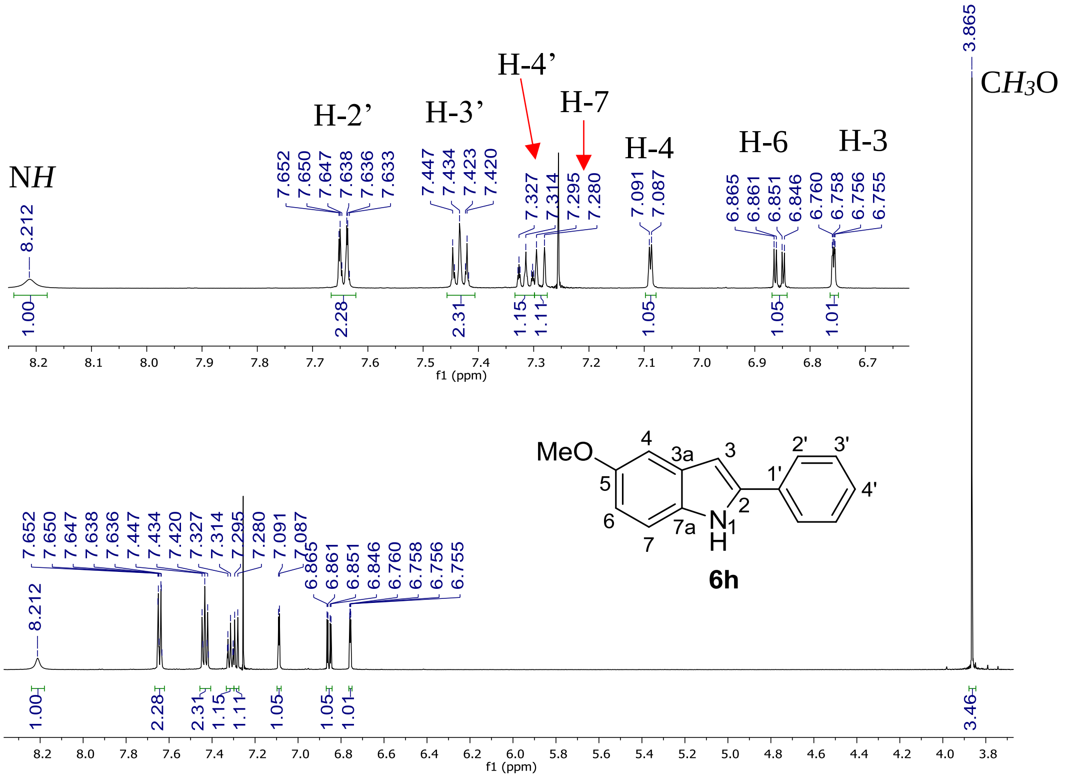

Figure S41. Spectrum of ^{1}H NMR (600 MHz, $CDCl_3$) of **6h**.

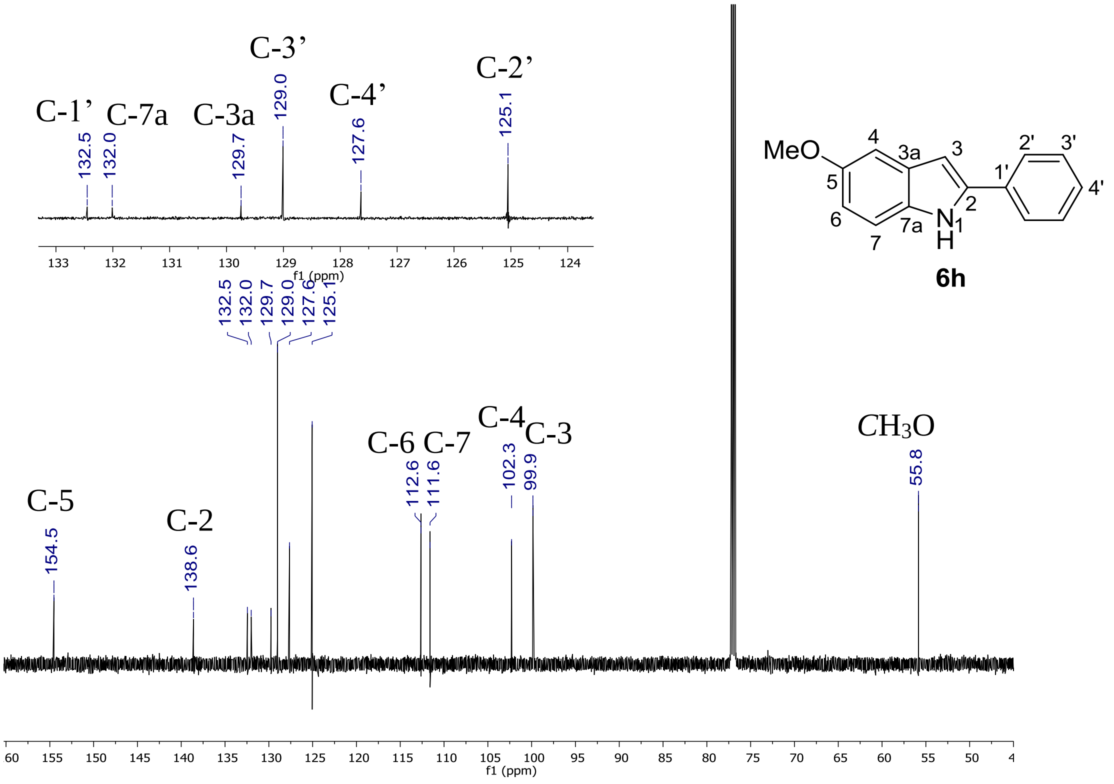

Figure S42. Spectrum of ^{13}C NMR (150 MHz, $CDCl_3$) of **6h**.

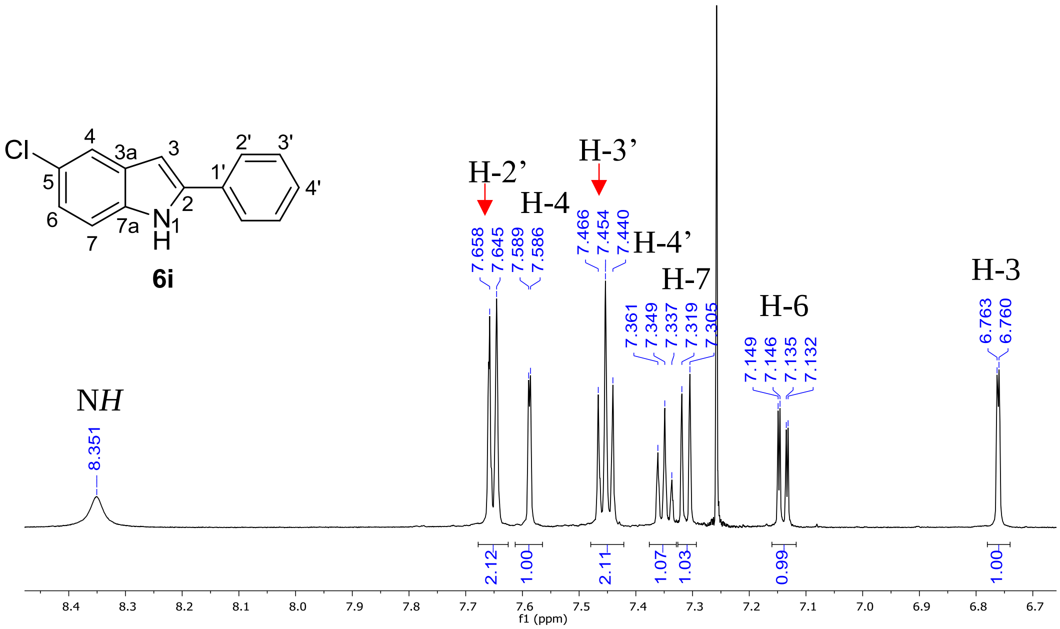

Figure S43. Spectrum of ^{1}H NMR (600 MHz, $CDCl_3$) of **6i**.

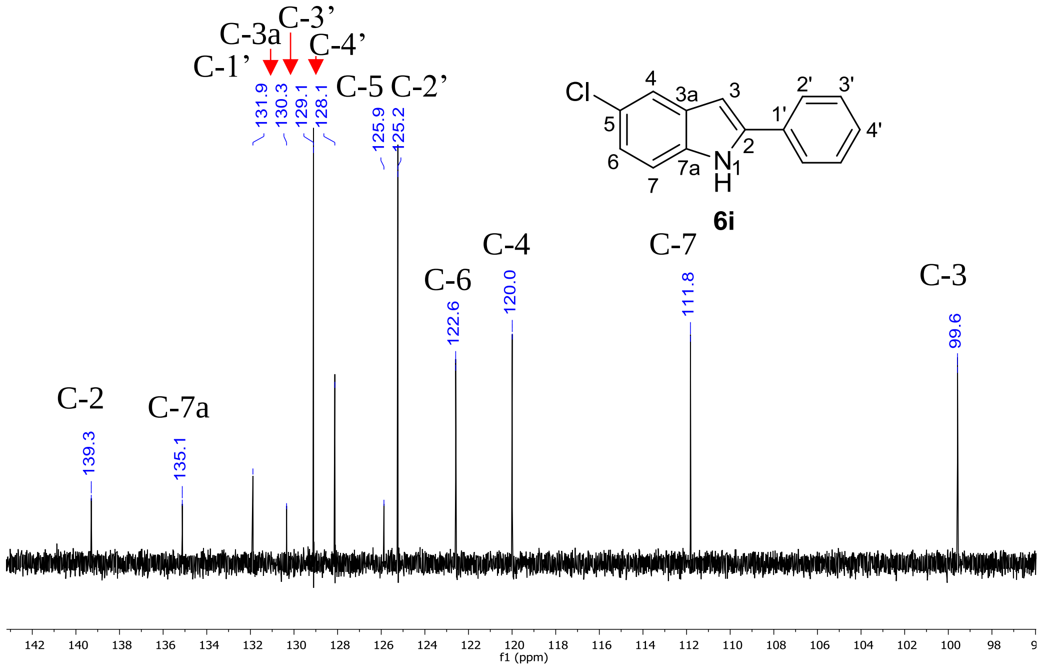

Figure S44. Spectrum of ^{13}C NMR (150 MHz, $CDCl_3$) of **6i**.

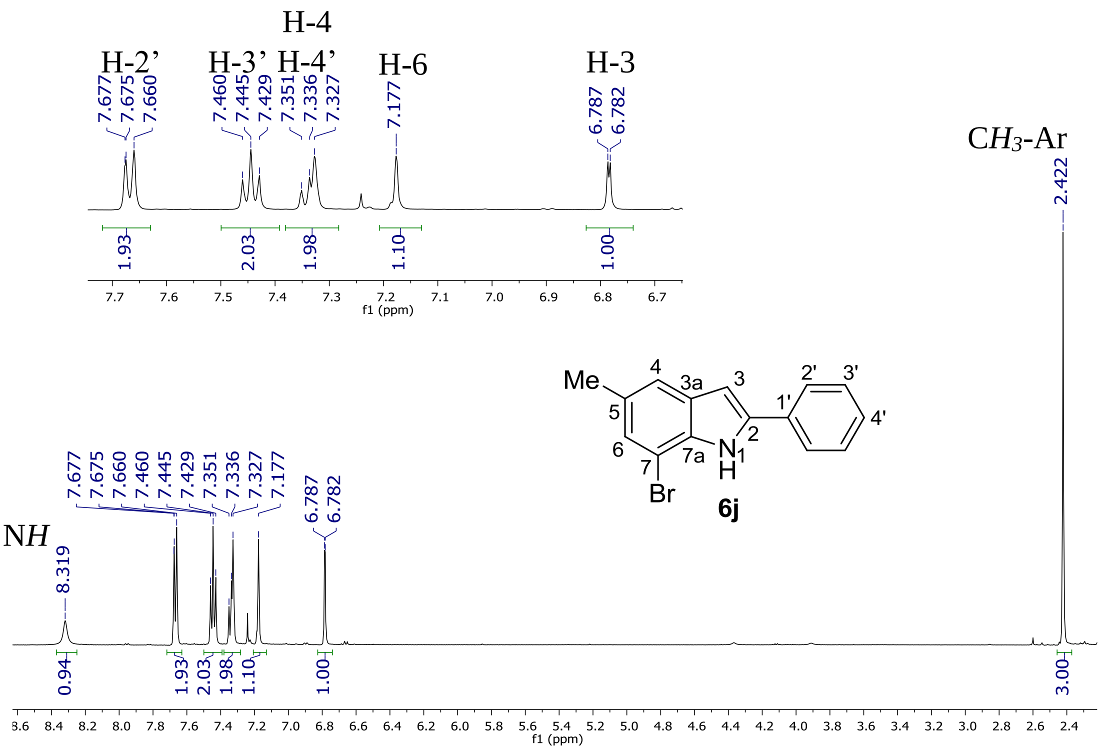

Figure S45. Spectrum of ^{1}H NMR (500 MHz, $CDCl_3$) of **6j**.

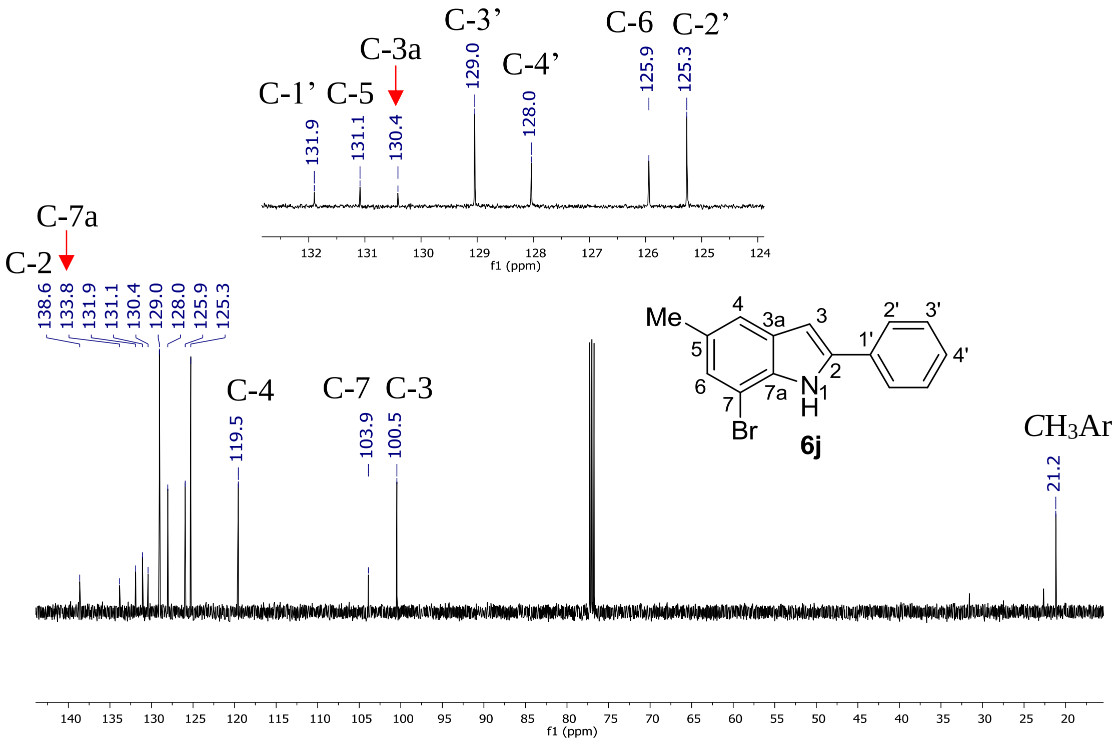

Figure S46. Spectrum of ^{13}C NMR (125 MHz, $CDCl_3$) of **6j**.

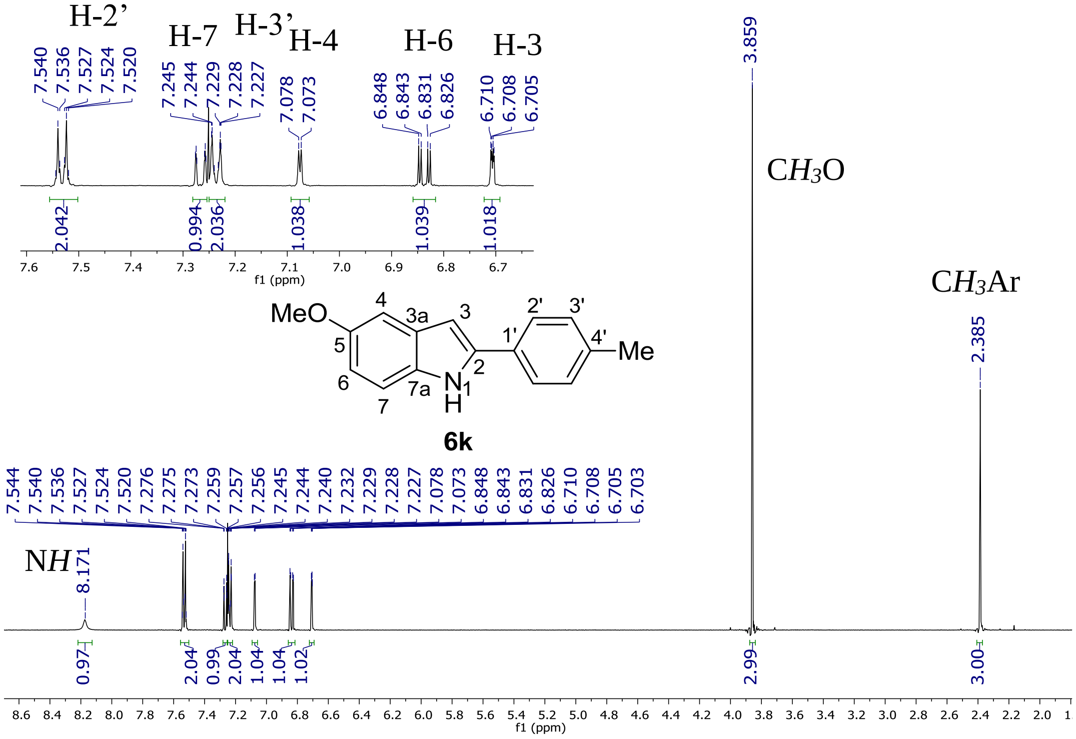

Figure S47. Spectrum of ^{1}H NMR (500 MHz, $CDCl_3$) of **6k**.

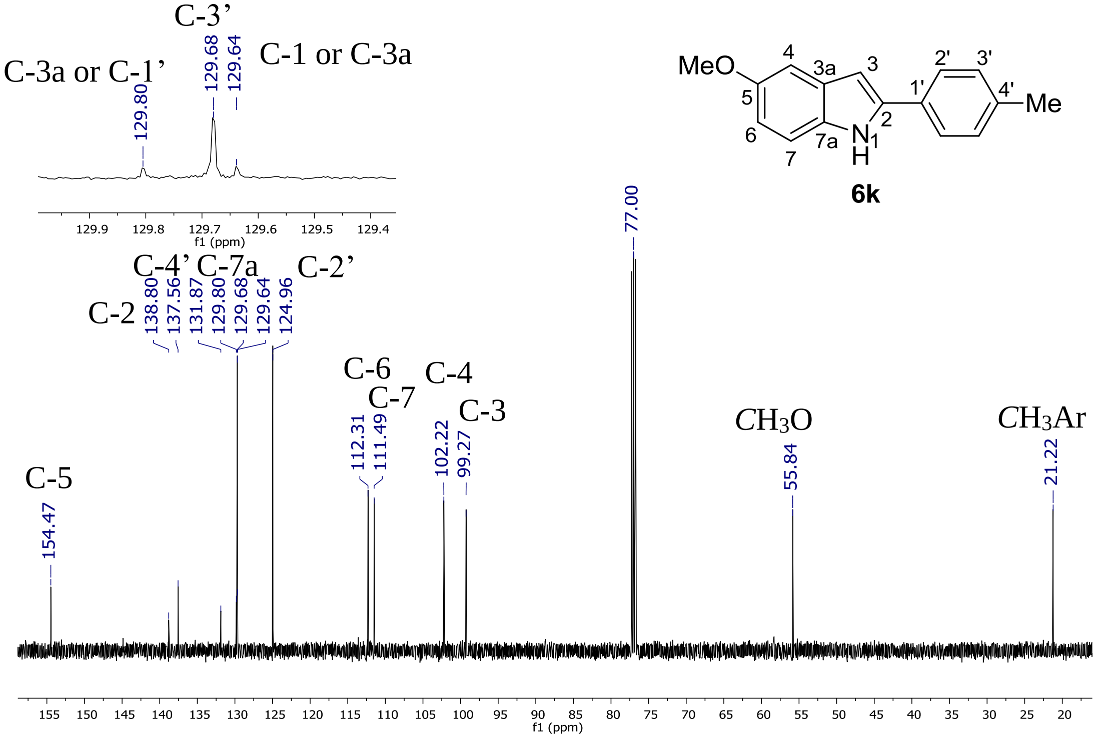

Figure S48. Spectrum of ^{13}C NMR (125 MHz, $CDCl_3$) of **6k**.

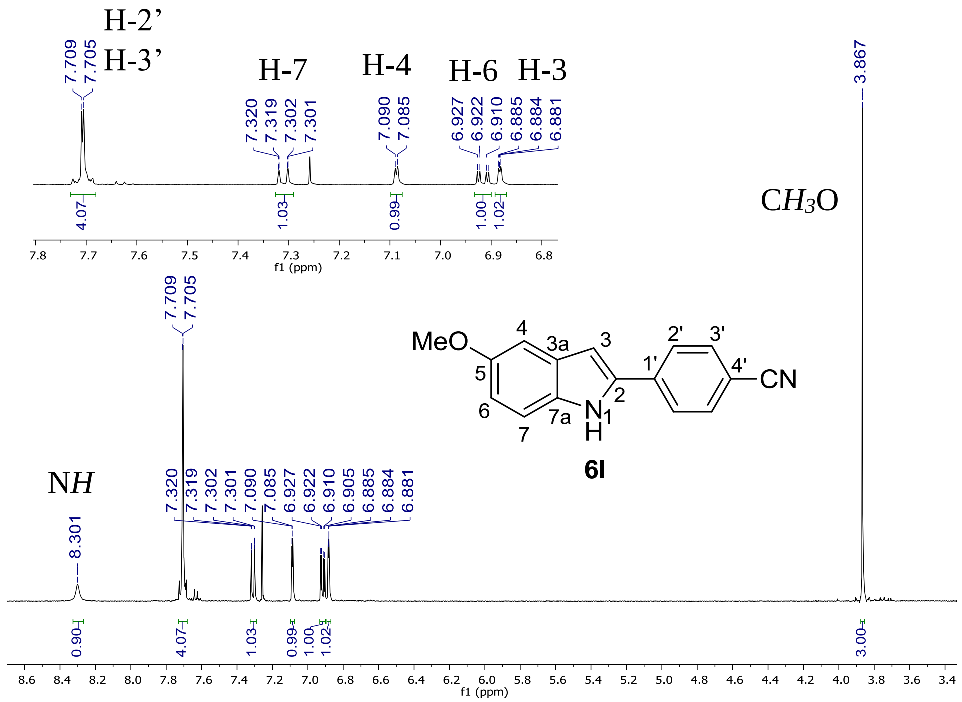

Figure S49. Spectrum of ^{1}H NMR (500 MHz, $CDCl_3$) of **6l**.

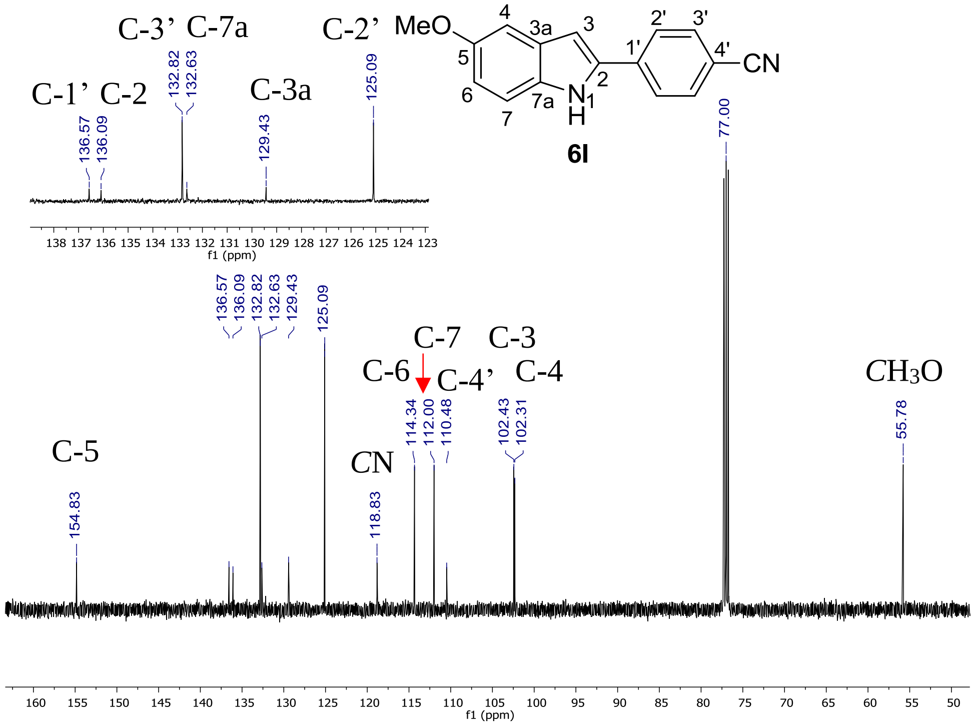

Figure S50. Spectrum of ^{13}C NMR (125 MHz, $CDCl_3$) of **6l**.

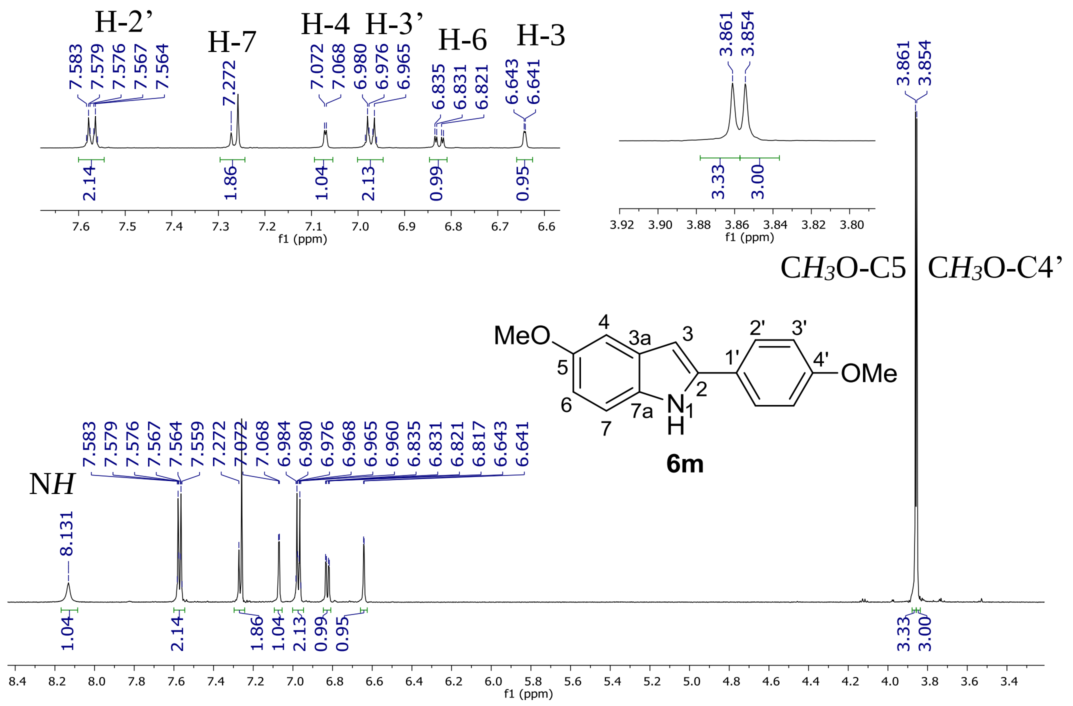

Figure S51. Spectrum of ^{1}H NMR (600 MHz, $CDCl_3$) of **6m**.

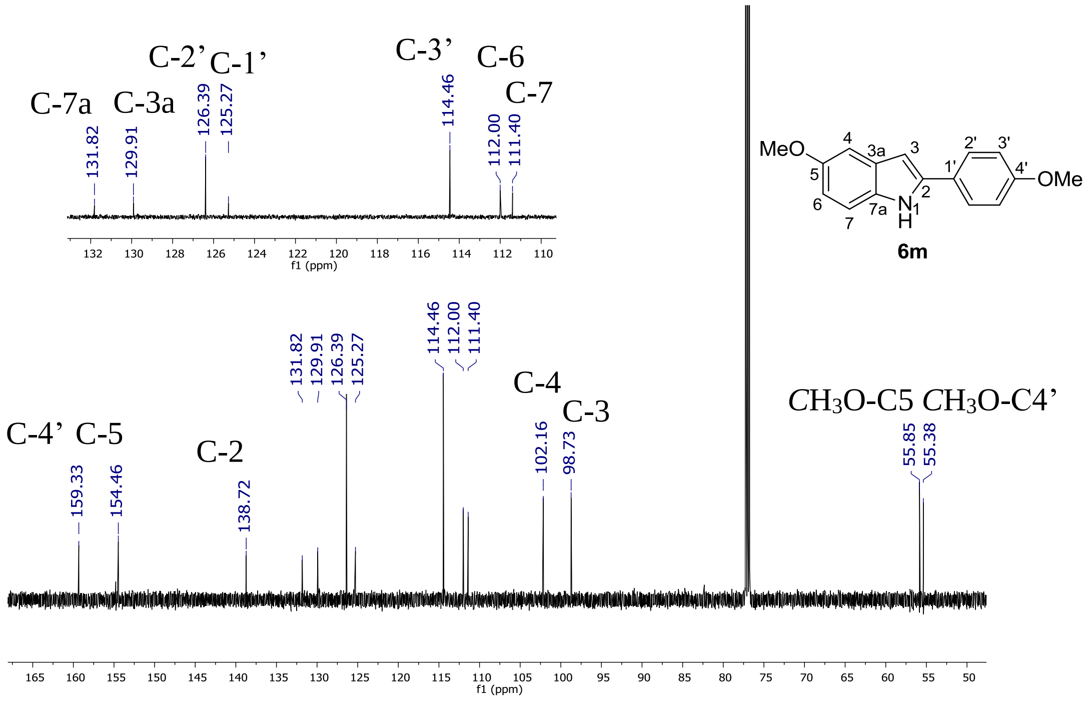

Figure S52. Spectrum of ^{13}C NMR (150 MHz, $CDCl_3$) of **6m**.

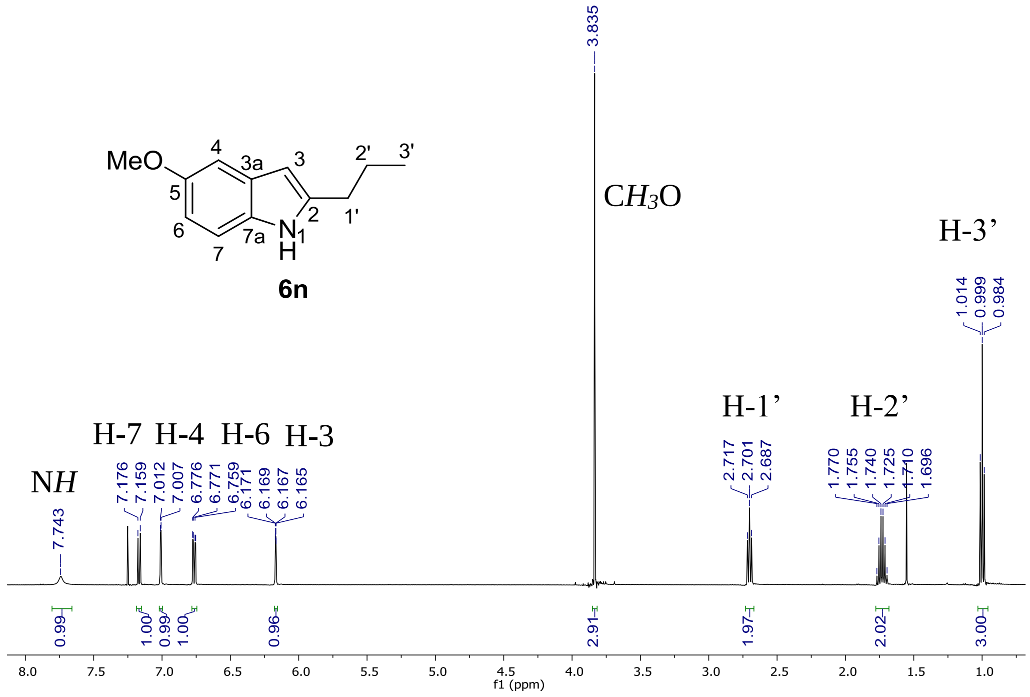

Figure S53. Spectrum of ^{1}H NMR (500 MHz, $CDCl_3$) of **6n**.

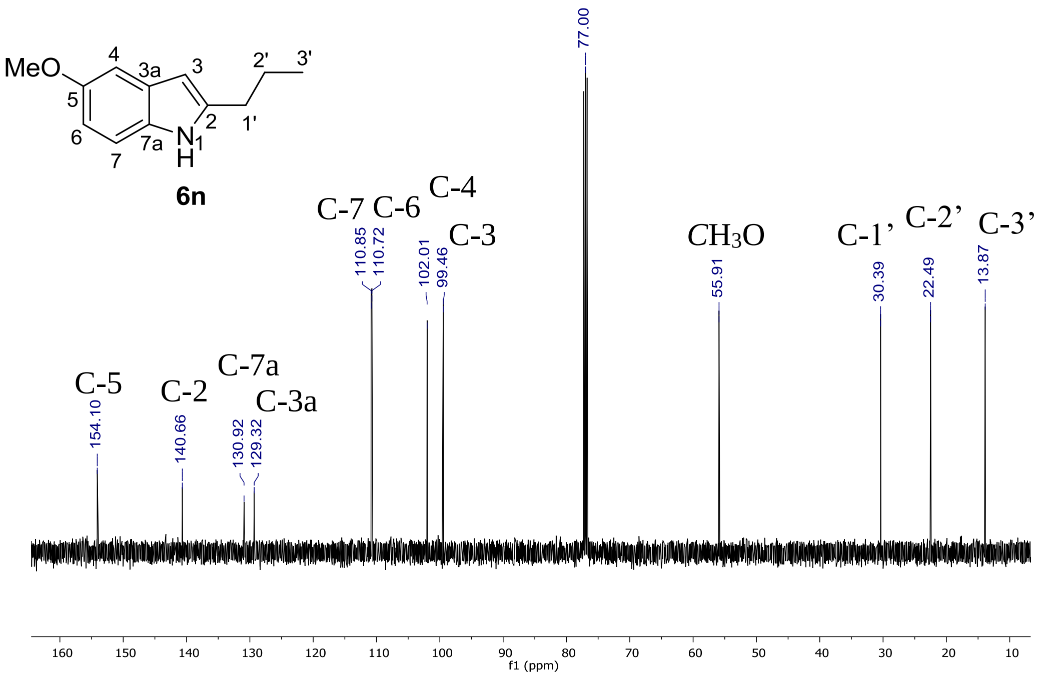

Figure S54. Spectrum of ^{13}C NMR (125 MHz, $CDCl_3$) of **6n**.

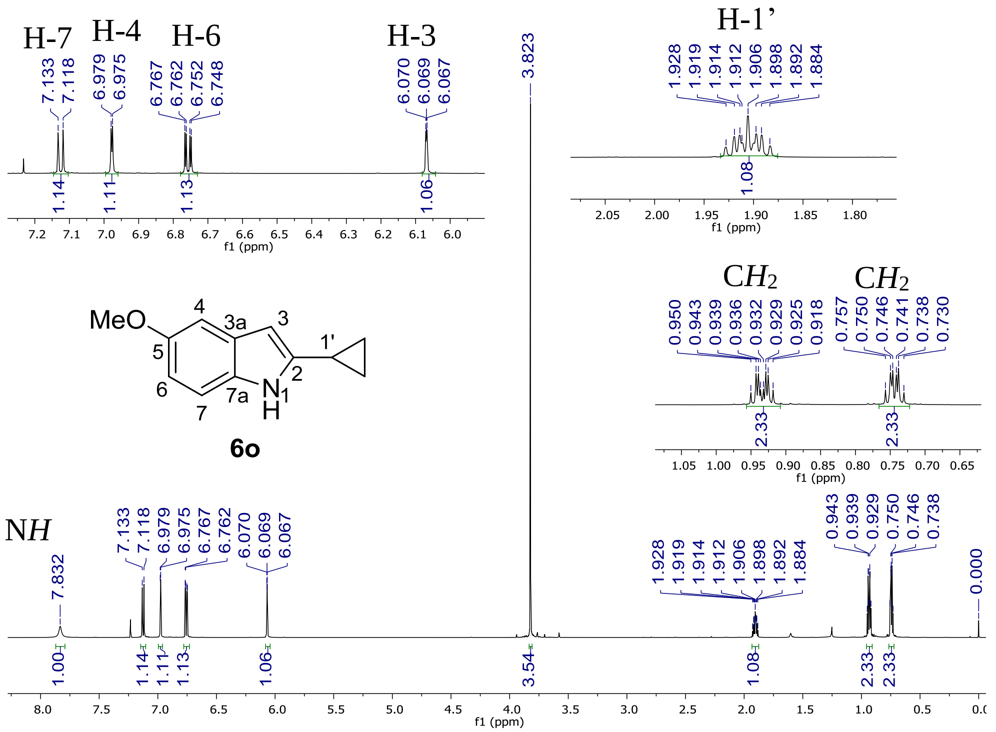

Figure S55. Spectrum of ^{1}H NMR (600 MHz, $CDCl_3$) of **6o**.

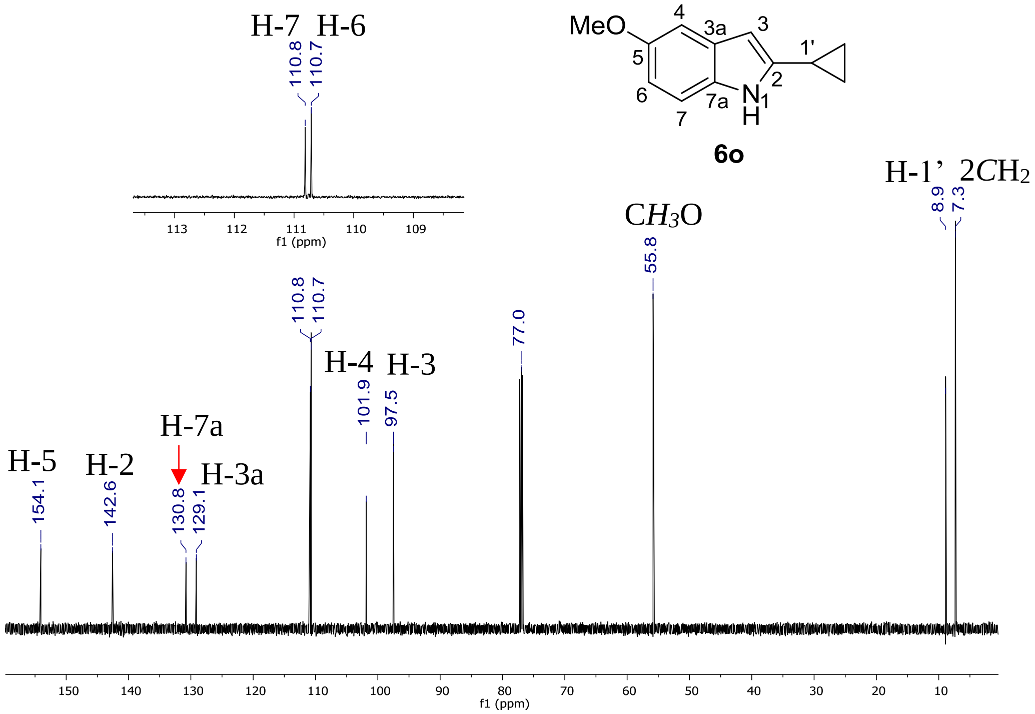

Figure S56. Spectrum of ^{13}C NMR (150 MHz, $CDCl_3$) of **6o**.

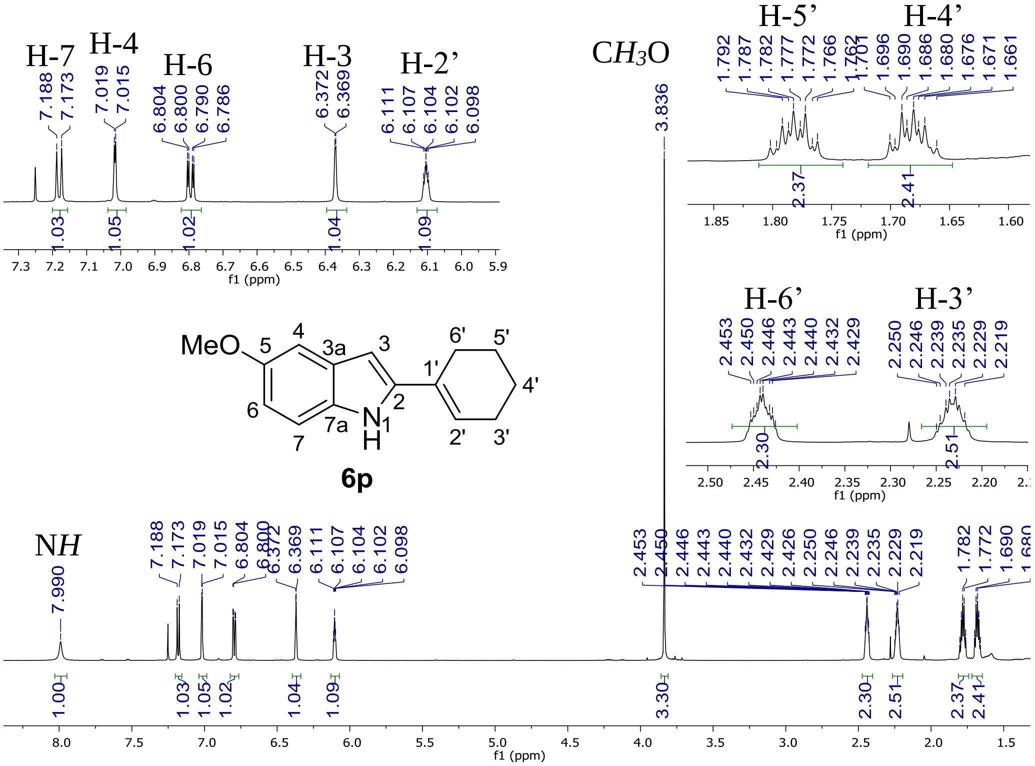

Figure S57. Spectrum of ^{1}H NMR (600 MHz, $CDCl_3$) of **6p**.

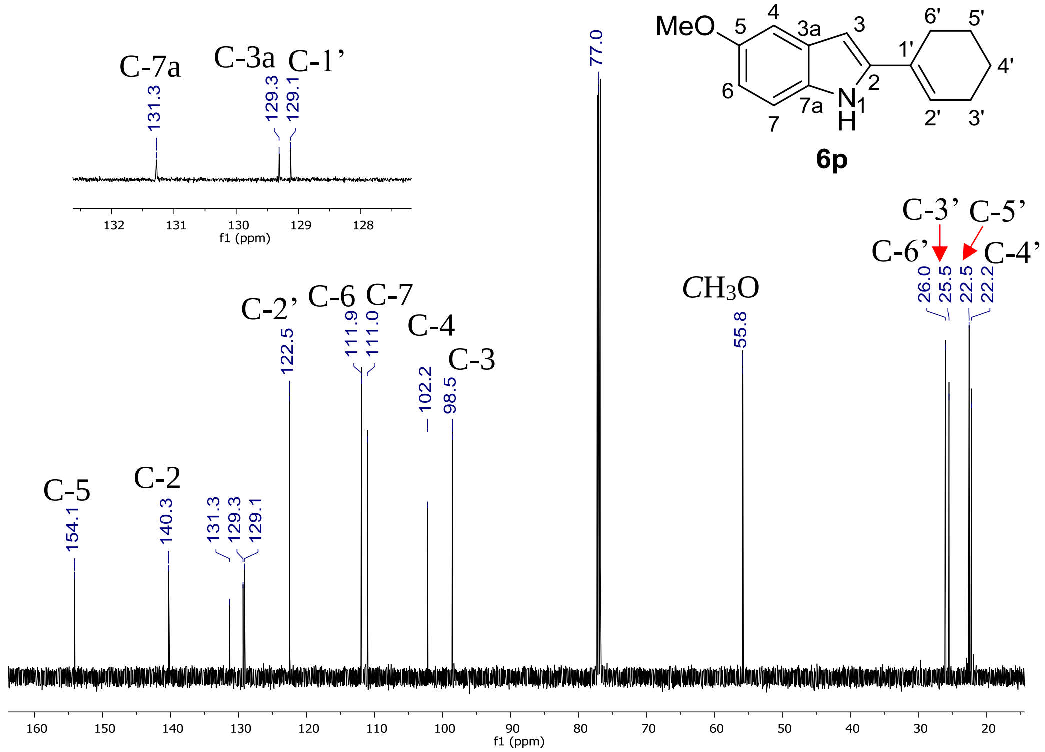

Figure S58. Spectrum of ^{13}C NMR (150 MHz, $CDCl_3$) of **6p**.

Table S1. Crystal data and structure refinement for **5i**.

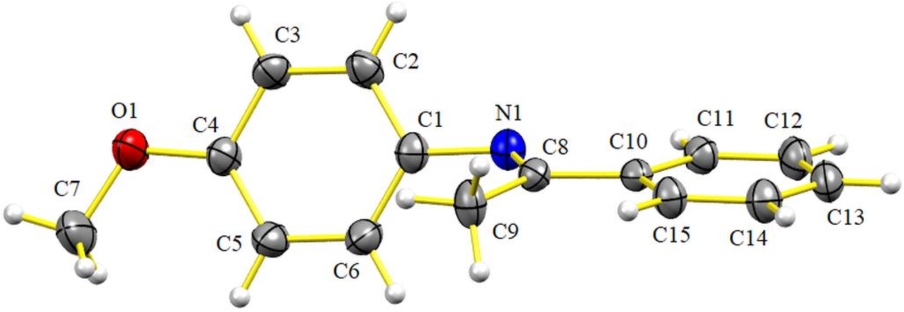

Identification code (CCDC deposition number)	2082632	
Empirical formula	C15 H15 N O	
Formula weight	225.28	
Temperature	292(2) K	
Wavelength	0.71073 Å	
Crystal system	Monoclinic	
Space group	P 21/n	
Unit cell dimensions	a = 10.0110(6) Å	α= 90°.
	b = 8.7430(5) Å	β= 104.172(7)°.
	c = 14.6687(11) Å	γ = 90°.
Volume	1244.82(14) $Å^3$	
Z	4	
Density (calculated)	1.202 Mg/m^3	
Absorption coefficient	0.075 mm^{-1}	
F(000)	480	
Crystal size	0.790 x 0.470 x 0.270 mm^3	
Theta range for data collection	3.655 to 29.183°.	
Index ranges	-9<=h<=13, -5<=k<=11, -19<=l<=12	
Reflections collected	5094	
Independent reflections	2839 [R(int) = 0.0177]	
Completeness to theta = 27.500°	95.5 %	
Refinement method	Full-matrix least-squares on F^2	
Data / restraints / parameters	2839 / 0 / 178	
Goodness-of-fit on F^2	1.042	
Final R indices [I>2sigma(I)]	R1 = 0.0505, wR2 = 0.1110	
R indices (all data)	R1 = 0.0777, wR2 = 0.1253	
Extinction coefficient	n/a	
Largest diff. peak and hole	0.130 and -0.154 e.$Å^{-3}$	

Table S2. Torsion angles [°] for **5i**.

C(1)-N(1)-C(8)-C(10)	176.72(13)
C(1)-N(1)-C(8)-C(9)	-3.7(2)
C(11)-C(10)-C(8)-N(1)	-4.7(2)
C(15)-C(10)-C(8)-N(1)	176.97(14)
C(11)-C(10)-C(8)-C(9)	175.72(15)
C(15)-C(10)-C(8)-C(9)	-2.6(2)
C(7)-O(1)-C(4)-C(5)	3.0(3)
C(7)-O(1)-C(4)-C(3)	-176.0(2)
C(11)-C(10)-C(15)-C(14)	-0.6(2)
C(8)-C(10)-C(15)-C(14)	177.78(14)
C(8)-N(1)-C(1)-C(6)	-66.8(2)
C(8)-N(1)-C(1)-C(2)	115.31(17)
C(15)-C(10)-C(11)-C(12)	0.4(2)
C(8)-C(10)-C(11)-C(12)	-178.07(14)
C(6)-C(1)-C(2)-C(3)	3.0(2)
N(1)-C(1)-C(2)-C(3)	-178.95(14)
C(1)-C(2)-C(3)-C(4)	-1.2(2)
O(1)-C(4)-C(3)-C(2)	177.55(15)
C(5)-C(4)-C(3)-C(2)	-1.5(2)
O(1)-C(4)-C(5)-C(6)	-176.73(15)
C(3)-C(4)-C(5)-C(6)	2.2(3)
C(12)-C(13)-C(14)-C(15)	0.0(3)
C(10)-C(15)-C(14)-C(13)	0.5(3)
C(14)-C(13)-C(12)-C(11)	-0.3(3)
C(10)-C(11)-C(12)-C(13)	0.1(2)
C(4)-C(5)-C(6)-C(1)	-0.3(3)
C(2)-C(1)-C(6)-C(5)	-2.3(3)
N(1)-C(1)-C(6)-C(5)	179.71(15)

Symmetry transformations used to generate equivalent atoms:

Table S3. Crystal data and structure refinement for **6e**.

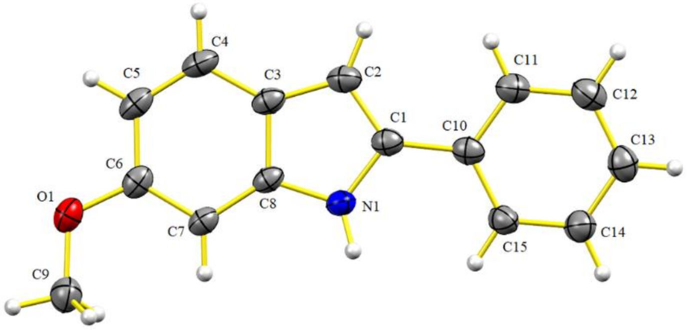

Identification code (CCDC deposition number)	2082633	
Empirical formula	C15 H13 N O	
Formula weight	223.26	
Temperature	292(2) K	
Wavelength	0.71073 Å	
Crystal system	Orthorhombic	
Space group	P 21 21 21	
Unit cell dimensions	a = 5.6782(3) Å	α= 90°.
	b = 8.0462(3) Å	β= 90°.
	c = 25.5690(16) Å	γ = 90°.
Volume	1168.19(11) $Å^3$	
Z	4	
Density (calculated)	1.269 Mg/m^3	
Absorption coefficient	0.080 mm^{-1}	
F(000)	472	
Crystal size	0.500 x 0.300 x 0.210 mm^3	
Theta range for data collection	3.187 to 29.557°.	
Index ranges	-7<=h<=6, -10<=k<=9, -19<=l<=34	
Reflections collected	3635	
Independent reflections	2352 [R(int) = 0.0132]	
Completeness to theta = 25.000°	99.8 %	
Refinement method	Full-matrix least-squares on F^2	
Data / restraints / parameters	2352 / 0 / 174	
Goodness-of-fit on F^2	1.056	
Final R indices [I>2sigma(I)]	R1 = 0.0387, wR2 = 0.0841	
R indices (all data)	R1 = 0.0468, wR2 = 0.0899	
Absolute structure parameter	0.3(9)	
Extinction coefficient	n/a	
Largest diff. peak and hole	0.095 and -0.147 e.$Å^{-3}$	

Table S4. Torsion angles [°] for **6e**.

C(1)-N(1)-C(8)-C(7)	-178.35(19)
C(1)-N(1)-C(8)-C(3)	1.4(2)
C(8)-N(1)-C(1)-C(2)	-1.4(2)
C(8)-N(1)-C(1)-C(10)	179.71(17)
C(9)-O(1)-C(6)-C(7)	2.8(3)
C(9)-O(1)-C(6)-C(5)	-177.33(19)
O(1)-C(6)-C(7)-C(8)	-179.65(17)
C(5)-C(6)-C(7)-C(8)	0.5(3)
N(1)-C(8)-C(7)-C(6)	-179.65(18)
C(3)-C(8)-C(7)-C(6)	0.6(3)
C(2)-C(1)-C(10)-C(11)	2.7(3)
N(1)-C(1)-C(10)-C(11)	-178.78(19)
C(2)-C(1)-C(10)-C(15)	-176.8(2)
N(1)-C(1)-C(10)-C(15)	1.8(3)
N(1)-C(8)-C(3)-C(4)	178.94(17)
C(7)-C(8)-C(3)-C(4)	-1.3(3)
N(1)-C(8)-C(3)-C(2)	-0.9(2)
C(7)-C(8)-C(3)-C(2)	178.94(18)
C(7)-C(6)-C(5)-C(4)	-1.0(3)
O(1)-C(6)-C(5)-C(4)	179.18(18)
C(15)-C(10)-C(11)-C(12)	-0.4(3)
C(1)-C(10)-C(11)-C(12)	-179.9(2)
C(11)-C(10)-C(15)-C(14)	0.5(3)
C(1)-C(10)-C(15)-C(14)	179.9(2)
N(1)-C(1)-C(2)-C(3)	0.9(2)
C(10)-C(1)-C(2)-C(3)	179.6(2)
C(4)-C(3)-C(2)-C(1)	-179.8(2)
C(8)-C(3)-C(2)-C(1)	0.0(2)
C(10)-C(15)-C(14)-C(13)	-0.2(3)
C(6)-C(5)-C(4)-C(3)	0.3(3)
C(8)-C(3)-C(4)-C(5)	0.8(3)
C(2)-C(3)-C(4)-C(5)	-179.5(2)
C(15)-C(14)-C(13)-C(12)	-0.2(3)
C(10)-C(11)-C(12)-C(13)	0.1(4)
C(14)-C(13)-C(12)-C(11)	0.2(3)

Symmetry transformations used to generate equivalent atoms:

Table S5. Crystal data and structure refinement for **6l**.

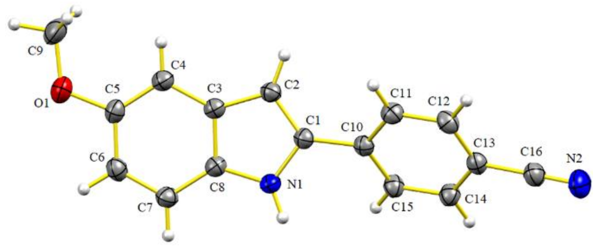

Identification code (CCDC deposition number)	2082634	
Empirical formula	C16 H12 N2 O	
Formula weight	248.28	
Temperature	292(2) K	
Wavelength	0.71073 Å	
Crystal system	Monoclinic	
Space group	P 21/c	
Unit cell dimensions	a = 7.0104(3) Å	α= 90°.
	b = 14.5242(8) Å	β= 98.202(4)°.
	c = 12.5032(7) Å	γ = 90°.
Volume	1260.06(11) $Å^3$	
Z	4	
Density (calculated)	1.309 Mg/m^3	
Absorption coefficient	0.084 mm^{-1}	
F(000)	520	
Crystal size	0.520 x 0.300 x 0.270 mm^3	
Theta range for data collection	2.936 to 29.453°.	
Index ranges	-9<=h<=9, -19<=k<=10, -15<=l<=11	
Reflections collected	5405	
Independent reflections	2909 [R(int) = 0.0154]	
Completeness to theta = 25.000°	99.8 %	
Refinement method	Full-matrix least-squares on F^2	
Data / restraints / parameters	2909 / 0 / 192	
Goodness-of-fit on F^2	1.022	
Final R indices [I>2sigma(I)]	R1 = 0.0450, wR2 = 0.1001	
R indices (all data)	R1 = 0.0633, wR2 = 0.1135	
Extinction coefficient	n/a	
Largest diff. peak and hole	0.138 and -0.163 e.$Å^{-3}$	

Table S6. Torsion angles [°] for **6l**.

C(1)-N(1)-C(8)-C(7)	-174.64(13)
C(1)-N(1)-C(8)-C(3)	2.11(15)
C(4)-C(3)-C(8)-N(1)	-179.11(11)
C(2)-C(3)-C(8)-N(1)	-1.53(14)
C(4)-C(3)-C(8)-C(7)	-2.03(18)
C(2)-C(3)-C(8)-C(7)	175.55(11)
C(8)-N(1)-C(1)-C(2)	-1.87(15)
C(8)-N(1)-C(1)-C(10)	176.22(11)
C(15)-C(10)-C(1)-C(2)	-149.99(14)
C(11)-C(10)-C(1)-C(2)	32.1(2)
C(15)-C(10)-C(1)-N(1)	32.41(18)
C(11)-C(10)-C(1)-N(1)	-145.55(13)
N(1)-C(1)-C(2)-C(3)	0.88(14)
C(10)-C(1)-C(2)-C(3)	-176.94(12)
C(4)-C(3)-C(2)-C(1)	177.45(13)
C(8)-C(3)-C(2)-C(1)	0.40(14)
C(8)-C(3)-C(4)-C(5)	1.78(18)
C(2)-C(3)-C(4)-C(5)	-174.98(13)
C(11)-C(10)-C(15)-C(14)	1.20(19)
C(1)-C(10)-C(15)-C(14)	-176.80(12)
C(3)-C(4)-C(5)-O(1)	-179.76(12)
C(3)-C(4)-C(5)-C(6)	-0.15(19)
C(9)-O(1)-C(5)-C(4)	1.8(2)
C(9)-O(1)-C(5)-C(6)	-177.87(14)
N(1)-C(8)-C(7)-C(6)	176.93(13)
C(3)-C(8)-C(7)-C(6)	0.56(19)
C(10)-C(15)-C(14)-C(13)	0.8(2)
C(12)-C(13)-C(14)-C(15)	-2.0(2)
C(16)-C(13)-C(14)-C(15)	176.11(12)
C(8)-C(7)-C(6)-C(5)	1.1(2)
C(4)-C(5)-C(6)-C(7)	-1.4(2)
O(1)-C(5)-C(6)-C(7)	178.30(12)
C(15)-C(10)-C(11)-C(12)	-1.95(19)
C(1)-C(10)-C(11)-C(12)	176.06(12)
C(10)-C(11)-C(12)-C(13)	0.7(2)
C(14)-C(13)-C(12)-C(11)	1.3(2)
C(16)-C(13)-C(12)-C(11)	-176.86(13)

Symmetry transformations used to generate equivalent atoms:
